# Immobilized Copper Complexes on Coal-Bearing Kaolin for Catalyzing Allylic Ester Synthesis via C(*sp*^3^)–H Bond Activation

**DOI:** 10.3390/molecules30102232

**Published:** 2025-05-21

**Authors:** Chun-Ling Zhang, Dao Su, Habuer Wang, Tegshi Muschin, Yun Wu, Yong-Sheng Bao, Huai-Yong Zhu

**Affiliations:** 1 Inner Mongolia Key Laboratory of Green Catalysis, College of Chemistry and Environmental Science, Inner Mongolia Normal University, Hohhot 010022, China; zwl15648207306@163.com (C.-L.Z.); 18404714387@163.com (D.S.); habur2004@163.com (H.W.); wy@imnu.edu.cn (Y.W.);; 2 School of Chemistry, Physics and Mechanical Engineering, Queensland University of Technology, 2 George Street, Brisbane 4001, Australia

**Keywords:** coal-bearing kaolin, immobilized copper catalyst, C(*sp*^3^)-H bond activation, cross-dehydrogenation coupling, synthesis of allylic ester

## Abstract

Copper complexes have attracted significant interest for catalyzing oxidative dehydrogenative carboxylation of alkanes to form esters. Here, we report a heterogeneous catalyst, in which copper complexes are immobilized on coal-bearing kaolin for the synthesis of allylic esters via C(*sp*^3^)-H bond activation through cross-dehydrogenation coupling reactions between cyclic alkanes and aromatic carboxylic acids. Systematic optimization of reaction conditions—including catalyst loading, copper content, oxidant, temperature, and reaction time—resulted in a high yield of 71% of allylic ester, comparable to homogeneous transition metal catalysts. The catalyst is easily recoverable via centrifugation and retains its activity over five consecutive reuse cycles. This system demonstrates broad substrate compatibility with various aromatic carboxylic acids and cyclic alkanes. Beyond offering an efficient and reusable catalytic route for allylic ester synthesis, this work highlights the potential of coal-bearing kaolin as a sustainable support material for transition metal catalysis and provides an environmentally benign method for activating inert C(*sp^3^*)–H bonds.

## 1. Introduction

The functionalization of C-H bonds, including the oxidation of alkanes to alcohols or ketones and the dehydrogenation of alkanes to alkenes, is a significant focus in synthetic chemistry [1]. Allylic esters, in particular, have attracted increasing attention due to their presence in natural products [2,3], biological molecules [4], pharmaceuticals [5,6], and fine chemicals [7,8,9]. Recently, direct, efficient, and atom-economical methods for synthesizing allylic esters through C-X (X = C, O, N) bond formation have been widely explored [10].

Cross-dehydrogenation coupling (CDC) is a key strategy for creating C-X bonds by removing hydrogen through the oxidative activation of inert C-H bonds [11]. CDC does not require pre-functionalizing the substrate, making it a convenient, high-atom-economy, and environmentally benign approach [12]. While alkane dehydrogenation and allylic oxidation are each well-studied, their combination into a single synthetic step is relatively uncommon [13].

**Scheme 1:**
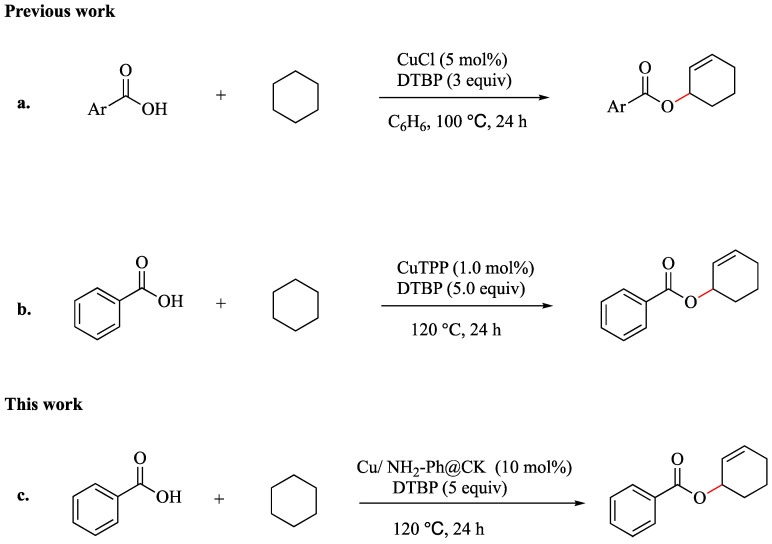
Strategies for homogeneous copper catalyzed synthesis of allylic ester via C(*sp*^3^)-H activation CDC reactions between alkanes and carboxylic acids, reported by Hartwig et al. (**a**), Liu group (**b**) and our approach (**c**).

Copper-based catalysts have shown promise in CDC reactions between alkanes and carboxylic acids. In 2014, Hartwig and coworkers developed a CuCl-catalyzed reaction that uses di-tert-butyl peroxide (DTBP) to oxidize alkanes and couple them with various benzoic acids (Scheme 1a) [13]. This work builds on the classic Kharasch–Sosnovsky reaction but substitutes alkanes for alkenes. In 2021, Liu and colleagues reported that difunctional copper porphyrins facilitate the coupling of carboxylic acids and cyclohexane to yield either allylic esters or alkyl olefins, depending on the carboxylic acid substrate (Scheme 1b) [14]. This copper porphyrin system offers short reaction times and broad functional group tolerance and eliminates the need for a base or solvent.

Here, we introduce a heterogeneous copper-loaded kaolin catalyst for the CDC reaction of cyclic alkanes with aromatic carboxylic acids, using DTBP as the oxidant (Scheme 1c). The optimized catalyst provides high yields, robust stability, and efficient recyclability.

Coal-bearing kaolin (CK), a byproduct of coal production, mainly contains kaolinite, which features a 1:1 layered structure of tetrahedral silica and octahedral alumina sheets [15]. Although kaolin is conventionally used in ceramics and coatings [16], recent studies highlight its potential in catalysis when doped with transition metals [17,18,19,20,21,22]. Our group’s previous research showed that palladium-loaded kaolin promotes directed C–H functionalization of arylpyrazoles [18], whereas iron-loaded kaolin enables direct synthesis of carboxylic esters via the C(*sp*^3^)-H activation of cyclic ethers [16]. More recently, we utilized iron-loaded kaolin to convert olefins and carboxylic acids into allylic esters by activating allylic C(*sp*^3^)-H bonds [21]. In the present work, we extend this concept to a copper-loaded kaolin catalyst designed for the direct CDC reaction of alkanes with aromatic carboxylic acids, targeting allylic ester formation.

## 2. Results and Discussion

CK was grafted with two silanes, 3-aminopropyltriethoxysilane and phenyltrimethoxysilane, followed by treatment with CuCl_2_ solution to form surface-immobilized copper complexes. Detailed synthesis procedures are available in the Supporting Information. The catalysts were characterized using X-ray diffraction (XRD), Brunauer–Emmett–Teller (BET) surface area measurements, elemental analysis, X-ray photoelectron spectroscopy (XPS), and transmission electron microscopy (TEM).

**Figure 1:**
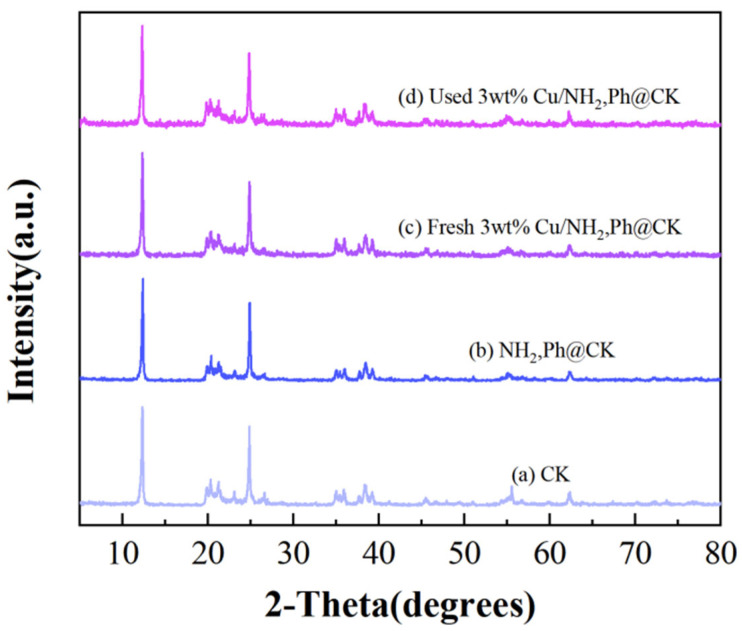
XRD patterns of (a) untreated coal-bearing kaolin (CK), (b) organically modified coal-bearing kaolin (NH_2_-Ph@CK), (c) fresh copper-loaded catalyst (fresh 3%Cu/NH_2_-Ph@CK), and (d) used copper-loaded catalyst (used 3%Cu/NH_2_-Ph@CK).

XRD patterns of unmodified CK, CK modified with both silanes (labeled as NH_2_-Ph@CK), and fresh and used copper-loaded catalyst (3%Cu/NH_2_-Ph@CK) are shown in Figure 1. No new diffraction peaks associated with copper species were detected after surface modification or metal loading, indicating that the kaolinite framework remained structurally intact.

BET surface areas and copper contents are summarized in Table 1. The raw and modified CK exhibited BET surface areas of 16 and 11 m^2^/g, respectively (entries 1–2). Copper loading had a minimal impact on surface area (15 m^2^/g, entry 3). Interestingly, the surface area increased to 20 and 30 m^2^/g after one and five reuse cycles, respectively (entries 4–5). Elemental analysis confirmed no detectable copper in unmodified or organically modified CK, while the fresh 3%Cu/NH_2_-Ph@CK contained 2.9 wt% Cu (entry 3), slightly decreasing to 2.7 wt% and 2.5 wt% after one and five reuse cycles (entries 4 and 5), respectively, indicating good copper retention during reuse.

**Figure 2:**
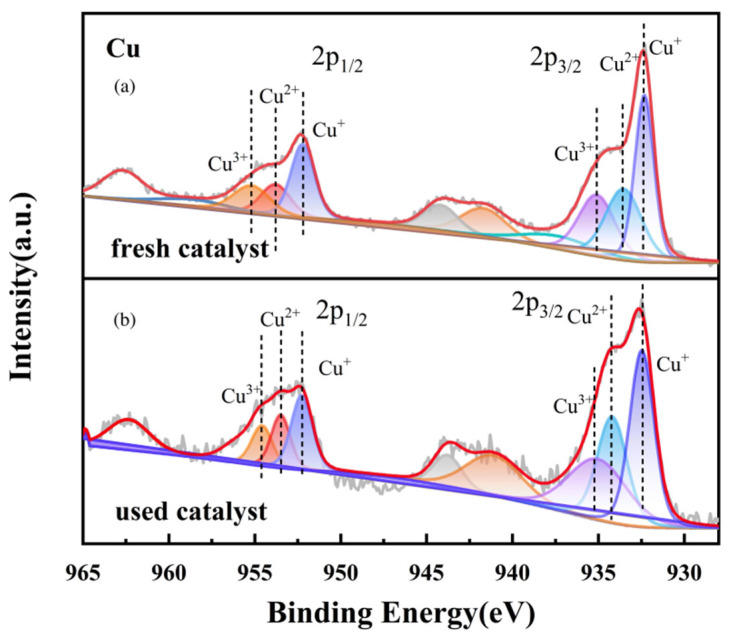
Cu 2P XPS spectra of (**a**) fresh 3%Cu/NH_2_-Ph@CK (binding energies 952.2 and 932.3 eV are assigned to Cu(I) (2p_1/2_ and 2p_3/2_); binding energies 953.8 and 933.5 eV are assigned to Cu(II) (2p_1/2_ and 2p_3/2_); binding energies 955.2 and 935.1 eV are assigned to Cu(III) (2p_1/2_ and 2p_3/2_)); (**b**) used 3%Cu/NH_2_-Ph@CK (binding energies 952.3 and 932.5 eV are assigned to Cu(I) (2p_1/2_ and 2p_3/2_); binding energies 953.5 and 934.2 eV are assigned to Cu(II) (2p_1/2_ and 2p_3/2_); binding energies 955.8 and 935.1 eV are assigned to Cu(III) (2p_1/2_ and 2p_3/2_)).

XPS analysis was used to determine copper oxidation states in the fresh and used 3%Cu/NH_2_-Ph@CK catalysts (Figure 2). Binding energies for Cu(I), Cu(II), and Cu(III) were observed at ~952/932 eV, ~953/934 eV, and ~955/935 eV (2p_1/2_ and 2p_3/2_), respectively. The fresh catalyst primarily contained Cu(II) and Cu(I), with a minor Cu(III) component. After reuse, the relative intensities of Cu(I) and Cu(III) increased, suggesting an active Cu(I)/Cu(II)/Cu(III) redox cycle during catalysis.

**Figure 3:**
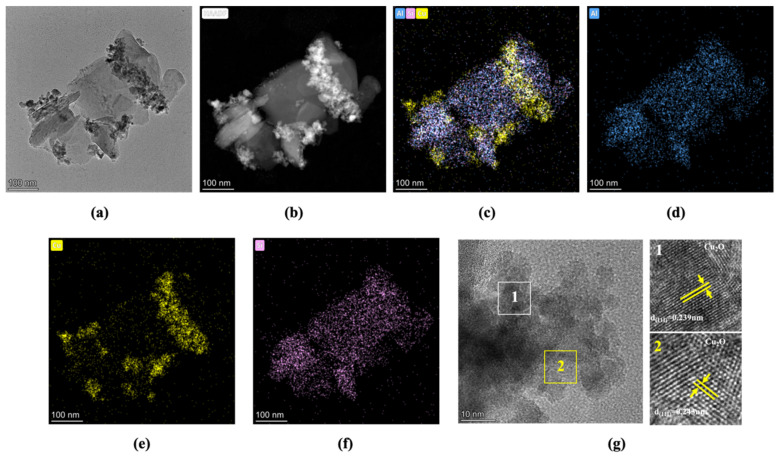
(**a**) shows 100 nm TEM images of 3%Cu/NH_2_-Ph@CK; (**b**) 100 nm HAADF-STEM images of 3%Cu/NH_2_-Ph@CK; (**c**–**e**) 100 nm TEM/EDS elemental mapping of 3%Cu/NH_2_-Ph@CK; (**c**) Al, Si and Cu element mapping; (**d**) Al element mapping; (**e**) Cu element mapping; (**f**) Si element mapping; (**g**) 10 nm HRTEM images of 3%Cu/NH_2_-Ph@CK.

TEM analysis (Figure 3) confirmed uniform copper dispersion across the CK surface. Elemental mapping and EDS spectra supported the presence and even distribution of copper. High-resolution TEM (HRTEM) images revealed lattice fringes with an interplanar spacing of approximately 0.24 nm, consistent with the (111) planes of Cu_2_O (Figure 3g), further verifying the formation of copper oxide species.

The catalytic efficiency of the catalyst for the synthesis of allylic ester via C(*sp*^3^)-H bond activation of alkane in a CDC reaction with aromatic carboxylic acids was assessed. Benzoic acid (**1**) and cyclohexane (**a**) were selected as model reactants.

Table 2 presents the catalytic performance of various kaolin-based catalysts under identical reaction conditions: 3%Cu/NH_2_-Ph@CK exhibited the highest catalytic activity, producing an allylic ester with a yield of 65% (Table 2, entry 13). Control experiments showed that without a catalyst or with raw CK and organically modified CK, only trace amounts of the product were detected (Table 2, entries 1–5). Copper loaded on kaolin catalyst with individual silane modifications—3%Cu/NH_2_@CK and 3%Cu/Ph@CK—produced lower yields (~58%, Table 2, entries 6–8) compared to 3%Cu/NH_2_-Ph@CK, indicating that dual-silane modification enhances catalytic efficiency. Notably, no significant difference was observed between copper loaded on unmodified kaolin and single-silane-modified kaolin, further confirming the synergistic effect of dual-silane grafting. All the reactions were carried out using DTBP as the oxidant and stirring at 120 °C for 24 h.

The influence of catalyst loading and copper content on reaction yield was systematically investigated. Increasing the catalyst amount from 1 mol% to 10 mol% (with respect to reactant **1**) improved the allylic ester yield from 56% to 65% (Table 2, entries 9–13). Similarly, varying the copper content (1–7 wt%) at a fixed catalyst loading (10 mol%) demonstrated that a 3 wt% Cu loading yielded the highest conversion at 3 wt% copper content (Table 2, entries 13–17), while higher copper content led to reduced yield. Comparisons with homogeneous copper catalysts (CuCl_2_, CuCl, CuO, and CuI) confirmed the superior efficiency of the heterogeneous catalyst 3%Cu/NH_2_-Ph@CK.

Oxidants play a crucial role in C-H activation. Table 3 summarizes the effect of different oxidants. Without oxidant, no product was detected (Table 3, entry 1). Common oxidants such as H_2_O_2_, K_2_S_2_O_8_, CH_3_COOOH, and 1,4-Benzoquine were ineffective. Tert-butyl hydroperoxide (TBHP) in H_2_O or nonane resulted in low yields (14% and 16%, respectively; Table 3, entries 10–11). DTBP emerged as the optimal oxidant, providing a maximum yield of 71% when used at 5 equiv. in 1 mL of cyclohexane (Table 3, entry 5).

**Figure 4:**
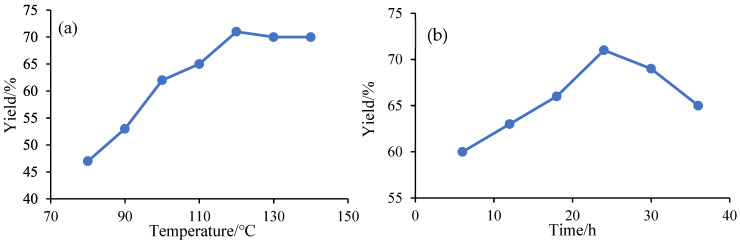
Influence of reaction temperature (**a**) and time (**b**) on the product yield.

The reaction temperature was optimized by varying it between 80 °C and 140 °C (Figure 4a), with the highest yield observed at 120 °C. Similarly, time optimization (6–48 h at 120 °C) indicated that 24 h was optimal (Figure 4b).

A thermal filtration experiment confirmed that the reaction occurred on the heterogeneous catalyst. Filtration of the reaction mixture halted further conversion, ruling out the presence of leached copper species as active catalysts (Table 4).

The scope of the catalytic reaction was explored with different benzoic acid derivatives (Table 5). Benzoic acid with electron-donating groups (Me-, MeO-, Ph-, PhO-, *t*-Bu-) yielded 47–65% of the allylic ester, with para-substituted derivatives showing higher yields than ortho- and meta-substituted counterparts, likely due to steric effects. Electron-withdrawing groups (halogens) resulted in a yield of 30–64%, whereas multiple electron-withdrawing groups led to lower yields (**23a**–**24a**). Certain substrates, including hydroxyl- (**26a**) and nitro- (**27a**) substituted benzoic acids, did not produce the desired product. However, 2-naphthoic acid (**25a**, 65%), 4-cyanobenzoic acid (**26a**, 20%), furan-2-carboxylic acid (**29a**, 20%), and thiophen-2-carboxylic acid (**30a**, 42%) successfully reacted with cyclohexane.

The scope of alkanes was also evaluated: cyclopentane (**1b**) and cyclooctane (**1c**) yielded 46% and 51% of the corresponding allylic esters, respectively.

**Figure 5:**
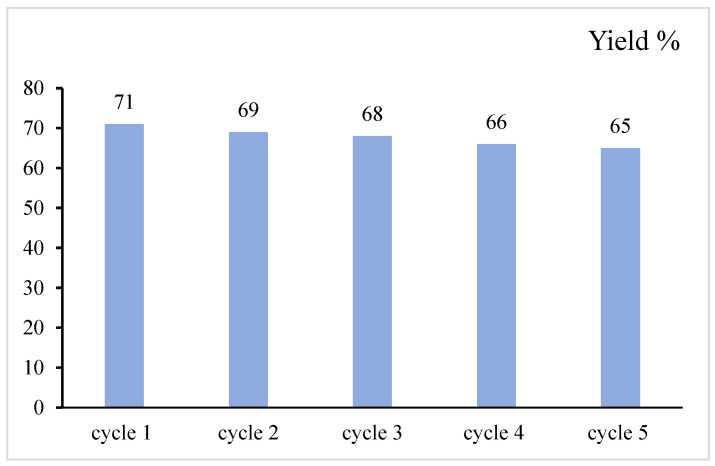
Recyclability of catalysts.

Heterogeneous catalysts offer advantages in recyclability compared to their homogeneous counterparts. The reusability of 3%Cu/NH_2_-Ph@CK was tested in the model reaction of benzoic acid and cyclohexane. After five cycles, the catalyst retained its performance, consistently yielding 65% of the product (Figure 5). These results highlight the catalyst’s potential for sustainable applications in green chemistry.

**Scheme 2:**
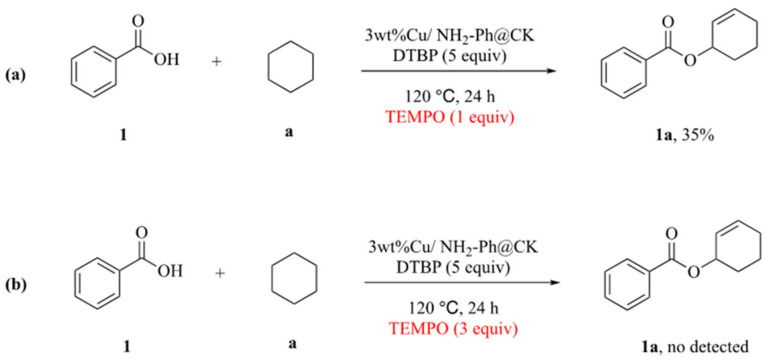
Radical trapping experiments with 1 equiv. (**a**) and 3 equiv. (**b**) TEMPO.

To investigate the reaction mechanism underlying the copper-catalyzed CDC reaction between carboxylic acid and alkanes, radical trapping experiments were performed (Scheme 2). When 0.3 mmol of benzoic acid (**1**) was reacted with 1 mL of cyclohexane (**a**) in the presence of 1 equivalent of the radical scavenger TEMPO (2,2,6,6-tetramethylpiperidine-N-oxyl), the yield of the allylic ester product dropped to 35%. Increasing TEMPO to 3 equivalents completely suppressed product formation, indicating the involvement of radical intermediates in the reaction pathway.

**Scheme 3:**
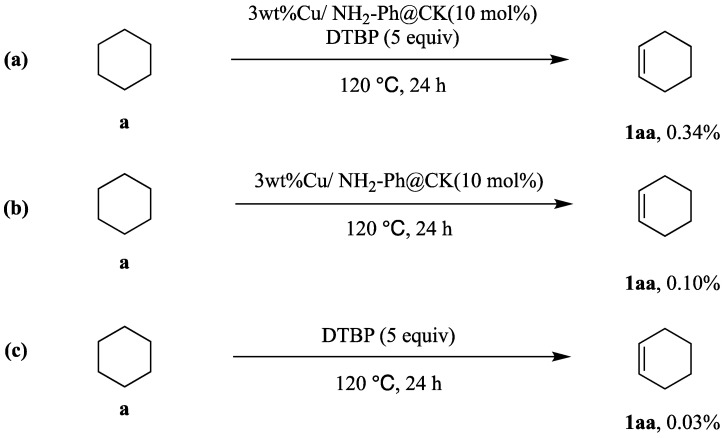
Control experiments for check the conversion of cyclohexane to cyclohexene. (**a**) Added catalyst and oxidant; (**b**) added catalyst without oxidant; and (**c**) just added oxidant, without catalyst. Yields of cyclohexene were calculated from the hydrogen ratio of cyclohexene and cyclohexane of ^1^H NMR spectra (as shown in Figure S1).

To further clarify the origin of the alkene intermediate, we investigated the dehydrogenation of cyclohexane under various conditions (Scheme 3). Cyclohexene formation was observed when either the copper catalyst or oxidant (DTBP) was present, but the highest yield was achieved when both components were used concurrently, supporting their synergistic role in oxidative dehydrogenation. These findings are consistent with previously proposed mechanisms. Hartwig et al. [13] proposed an oxidative dehydrogenation (ODC) mechanism wherein copper-activated DTBP abstracts a hydrogen atom from cyclohexane to form a cyclohexyl radical. This radical undergoes oxidation through single-electron transfer, generating a carbocation intermediate that subsequently loses a proton to form cyclohexene. Further hydrogen abstraction yields an allylic radical that participates in esterification. Alternatively, Mondal et al. [23] proposed a Kharasch–Sosnovsky-type mechanism involving hydrogen atom transfer (HAT) from alkanes by tert-butyl radicals, forming alkene intermediates that proceed to ester products.

**Scheme 4:**
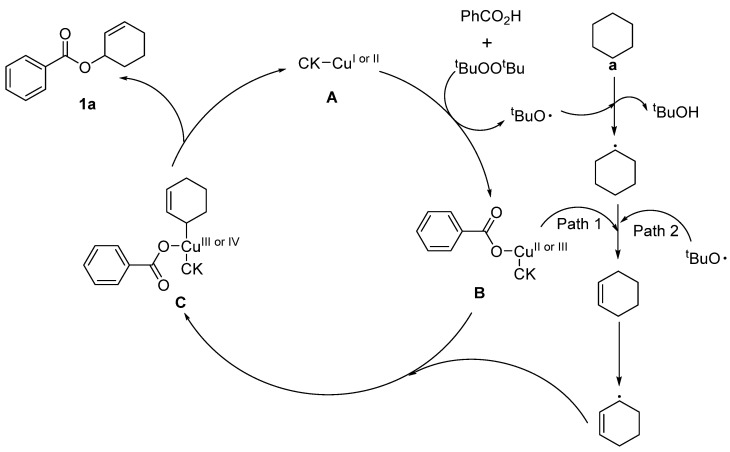
Proposed reaction mechanism.

Based on our results and literature precedents [13,14,22,23,24], we propose a tentative mechanism (Scheme 4). The catalytic screening results showed moderate activity for copper catalysts with oxidation states of +1 or +2 (Table 2, entries 18–19). XPS analysis (Figure 2) further confirmed the presence of both Cu(I) and Cu(II) species in the catalyst before and after reaction, consistent with redox cycling during catalysis. In the proposed pathway, the copper catalyst (A), initially in the +1 or +2 state, is oxidized by DTBP to form a higher-valent copper complex (B) coordinated with benzoic acid. Simultaneously, the *tert*-butyl radical generated from DTBP abstracts a hydrogen atom of cyclohexane, producing a cyclohexyl radical. We propose two pathways for cyclohexene formation:

1. Path 1: Oxidation of the cyclohexyl radical via single-electron transfer, yielding a carbocation intermediate, followed by deprotonation.
2. Path 2: HAT-driven direct dehydrogenation by tert-butyl radicals to form cyclohexene.

The resulting allylic radical then couples with complex B to form intermediate C, which decomposes to yield the allylic ester products **1a** and regenerate catalyst A.

## 3. Conclusions

In this study, we developed a recyclable copper-based heterogeneous catalyst for the direct esterification of benzoic acid and alkanes via C(*sp*^3^)-H bond activation. This catalytic system demonstrates high atom economy, environmental sustainability, and operational simplicity, aligning with the principles of green chemistry. Through systematic optimization of reaction parameters—including oxidant loading, substrate ratios, temperature, and time—we achieved improved yields of allylic esters. The copper-loaded coal-bearing kaolin catalyst exhibited excellent activity and selectivity. Notably, it performed well even with carboxylic acids bearing electron-donating groups or halogens, delivering moderate to good product yields.

The catalyst retained its activity after five reuse cycles and was easily separated and recovered, highlighting its practicality. Overall, this heterogeneous copper system offers a promising alternative to conventional homogeneous copper catalysts for C(*sp^3^*)–H bond activation, and paves the way for sustainable CDC reactions in organic synthesis.

## Figures and Tables

**Table 1:** Copper elemental analysis and specific surface area (S_BET_) results.

Entry	Sample	Cu (wt%)	S_BET_ (m^2^/g)
1	CK	0	16
2	NH_2_-Ph@CK	0	11
3	3%Cu/NH_2_-Ph@CK	2.9	15
4	Recycle once 3%Cu/NH_2_-Ph@CK	2.7	25
5	Recycle five 3%Cu/NH_2_-Ph@CK	2.5	30

**Table 2:** Effect of catalysts on the reaction *^a^*.

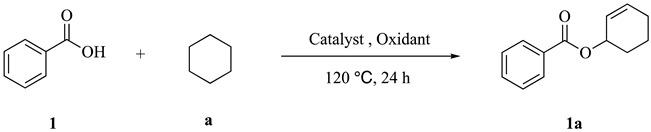		
Entry	Catalyst (mol%)	Yield (%) *^b^*
1	–	trace
2	CK (10)	trace
3	NH_2_@CK (10)	trace
4	Ph@CK (10)	trace
5	NH_2_-Ph@CK (10)	trace
6	3%Cu@CK (10)	58
7	3%Cu/NH_2_@CK (10)	58
8	3%Cu/Ph@CK (10)	59
9	3%Cu/NH_2_-Ph@CK (1)	56
10	3%Cu/NH_2_-Ph@CK (3)	58
11	3%Cu/NH_2_-Ph@CK (5)	58
12	3%Cu/NH_2_-Ph@CK (7)	59
13	3%Cu/NH_2_-Ph@CK (10)	65
14	1%Cu/NH_2_-Ph@CK (10)	57
15	2%Cu/NH_2_-Ph@CK (10)	59
16	5%Cu/NH_2_-Ph@CK (10)	54
17	7%Cu/NH_2_-Ph@CK (10)	53
18	CuCl_2_ (10)	48
19	CuCl (10)	55
20	CuO (10)	65
21	CuI (10)	62

*^a^* Reaction conditions: **1** (0.3 mmol), **a** (1.5 mL), oxidant: DTBP (5 equiv.), 120 °C, 24 h. *^b^* Isolated yield.

**Table 3:** Effect of oxidants on the reaction *^a^*.

Entry	Catalyst	Oxidant (Equiv.)	Yield (%) *^b^*
1	3%Cu/NH_2_-Ph@CK	–	N.D.
2	3%Cu/NH_2_-Ph@CK	DTBP (1)	8
3	3%Cu/NH_2_-Ph@CK	DTBP (3)	29
4	3%Cu/NH_2_-Ph@CK	DTBP (5)	65
5 *^c^*	3%Cu/NH_2_-Ph@CK	DTBP (5)	71
6 *^d^*	3%Cu/NH_2_-Ph@CK	DTBP (5)	52
7	3%Cu/NH_2_-Ph@CK	DTBP (10)	67
8	3%Cu/NH_2_-Ph@CK	DTBP (15)	67
9	3%Cu/NH_2_-Ph@CK	DTBP (20)	68
10	3%Cu/NH_2_-Ph@CK	TBHP (in H_2_O) (5)	14
11	3%Cu/NH_2_-Ph@CK	TBHP (in nonane) (5)	16
12	3%Cu/NH_2_-Ph@CK	H_2_O_2_ (5)	N.D.
13	3%Cu/NH_2_-Ph@CK	K_2_S_2_O_8_ (5)	N.D.
14	3%Cu/NH_2_-Ph@CK	CH_3_COOOH (5)	N.D.
15	3%Cu/NH_2_-Ph@CK	1,4-Benzoquine (5)	N.D.

Reaction conditions: 1 (0.3 mmol), a (1.5 mL), catalyst (10 mol%), 120 °C, 24 h. *^b^* Isolated yield. *^c^* **a** (1 mL). *^d^* **a** (2 mL).

**Table 4:** The thermal filtration experiments *^a^*.

Entry	Time/h	Yield/% *^b^*
1	4	30
2	8	31

*^a^* Reaction conditions: **1** (0.3 mmol), **a** (1 mL), catalyst (10 mol%), DTBP (5 equiv.), 120 °C, 24 h. *^b^* Isolated yield.

**Table 5:** Substrate scope for CDC of allyl esters *^a^*.

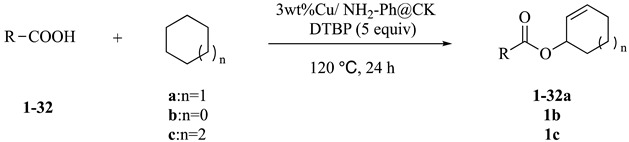			
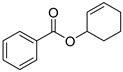	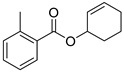	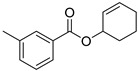	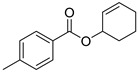
**1a**, 71%	**2a**, 50%	**3a**, 60%	**4a**, 65%
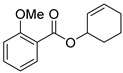	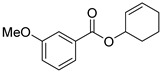	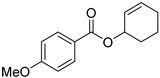	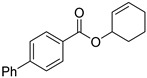
**5a**, 54%	**6a**, 60%	**7a**, 64%	**8a**, 47%
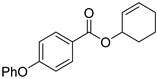	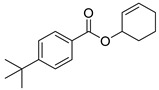	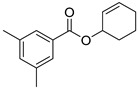	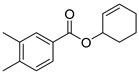
**9a**, 63%	**10a**, 48%	**11a**, 58%	**12a**, 56%
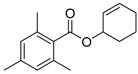	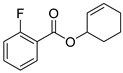	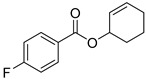	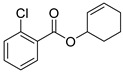
**13a**, 54%	**14a**, 61%	**15a**, 66%	**16a**, 58%
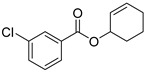	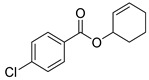	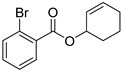	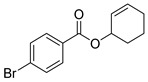
**17a**, 61%	**18a**, 64%	**19a**, 50%	**20a**, 64%
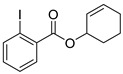	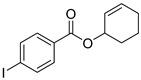	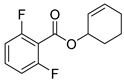	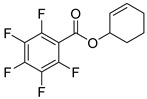
**21a**, 36%	**22a**, 54%	**23a**, 50%	**24a**, 43%
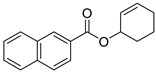	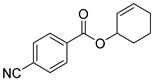	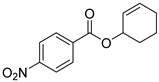	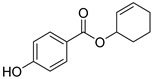
**25a**, 65%	**26a**, 20%	**27a**, N.D.	**28a**, N.D.
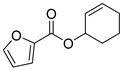	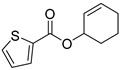	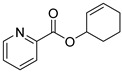	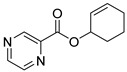
**29a**, 20%	**30a**, 42%	**31a**, N.D.	**32a**, N.D.
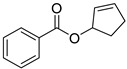	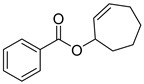		
**1b**, 46%	**1c**, 51%		

*^a^* Reaction conditions: **1**–**32** (0.3 mmol), **a**–**c** (1 mL), catalyst (10 mol%), DTBP (5 equiv.), 120 °C, 24 h. Isolated yield based on aromatic carboxylic acid. N.D., not detected.

## Data Availability

Data are contained within the article and Supplementary Materials.

## Supplementary Materials

The following supporting information can be downloaded at https://www.mdpi.com/article/10.3390/molecules30102232/s1. Figure S1: ^1^H NMR spectra of control experiments to check the conversion of cyclohexane to cyclohexene. Details of experimental section, particle characterization and ^1^H and ^13^C NMR spectra are given in the Supporting Information. Refs. [21,25] are cited within.

## References

[1] Shulpin G.B. (2013). C–H Functionalization: Thoroughly Tuning Ligands at a Metal ion, a Chemist can Greatly. Enhance Catalyst’s Activity and Selectivity. Dalton Trans..

[2] Nakamura A., Nakada M. (2013). Allylic oxidations in natural product synthesis. Synthesis.

[3] Uyeda C., Rötheli A.R., Jacobsen E.N. (2010). Catalytic enantioselective Claisen rearrangements of O-allyl β-ketoesters. Angew. Chem. Intl. Ed..

[4] Kirsch S.F., Overman L.E. (2005). Catalytic asymmetric synthesis of chiral allylic esters. J. Am. Chem. Soc..

[5] Pacheco M.C., Purser S., Gouverneur V. (2008). The chemistry of propargylic and allylic fluorides. Chem. Rev..

[6] Atmaca U., Kaya R., Karaman H.S. (2019). Synthesis of oxazolidinone from enantiomerically enriched allylic alcohols and determination of their molecular docking and biologic activities. Bioorg. Chem..

[7] Ren T.L., Xu B.H., Mahmood S. (2017). Cobalt-catalyzed oxidative esterification of allylic/benzylic C (*sp*^3^)-H bonds. Tetrahedron.

[8] Ankisetty S., ElSohly H.N., Li X.C. (2006). Aromatic Constituents of Uvaria Grandiflora. J. Nat. Prod..

[9] Guengerich F.P., Yoshimoto F.K. (2018). Formation and Cleavage of C–C Bonds by Enzymatic Oxidation–Reduction Reactions. Chem. Rev..

[10] Xiong M.F., Ali A., Akram W. (2019). Copper porphyrin as efficient catalysts for esterification of allyl *sp*^3^ C–H bond with carboxylic acid. Catal. Commun..

[11] Krylov I.B., Vil V.A., Terent’ev A.O. (2015). Cross-dehydrogenative coupling for the intermolecular C–O bond formation. Beilstein J. Org. Chem..

[12] Shan Z.W., Chen X.Y., Zhang H. (2022). Copper Porphyrin Catalyzed C (*sp*^3^)–H Activation via Cross-Dehydrogenative Coupling: Facile Transformation of Aldehydes to Esters. Synlett.

[13] Tran B.L., Driess M., Hartwig J.F. (2014). Copper-catalyzed oxidative dehydrogenative carboxylation of unactivated alkanes to allylic esters via alkenes. J. Am. Chem. Soc..

[14] Chen X.Y., Yang S., Ren B.P. (2021). Copper porphyrin-catalyzed cross dehydrogenative coupling of alkanes with carboxylic acids: Esterification and decarboxylation dual pathway. Tetrahedron.

[15] Detellier C. (2018). Functional Kaolinite. Chem. Rec..

[16] Bao U., Muschin T., Bao A. (2021). FeNP-loaded coal-bearing kaolin catalysts for the direct esterification of benzoic acid with cyclic ether via C (*sp*^3^)-H bond activation. Green Chem. Lett. Rev..

[17] Niu S., Xie X., Wang Z. (2021). Enhanced removal performance for Congo red by coal-series kaolin with acid treatment. Environ. Technol..

[18] Yang P., Bao Y.S. (2017). Palladium Nanoparticles Supported on Organofunctionalized Kaolin as an Efficient Heterogeneous Catalyst for Directed C–H Functionalization of Arylpyrazoles. RSC Adv..

[19] Muschin T., Duo X., Bao U., Zulchin H., Agula B. (2019). Environmentally Friendly Treatment of Coal-Bearing Kaolin by Polyhydroxy-Iron for Anionic Dye Removal. ChemistrySelect.

[20] Muschin T., Zulchin H., Jia M. (2021). Adsorption Behavior of Polyhydroxy-Iron-Modified Coal-Bearing Kaolin for Fluoride Removal. ChemistrySelect.

[21] Su D., Muschin T., Wu Y. (2024). Direct esterification of allylic C (*sp*^3^)–H via iron nanoparticle–loaded kaolin-catalyzed cross dehydrogenative coupling. Green Chem. Lett. Rev..

[22] Wang C.Y., Song R.J., Wei W.T. (2015). Copper-catalyzed oxidative coupling of acids with alkanes involving dehydrogenation: Facile access to allylic esters and alkylalkenes. Chem. Commun..

[23] Mondal R., Chakraborty G., van Vliet K.M. (2020). Copper-catalyzed oxidative dehydrogenative functionalization of alkanes to allylic esters. Inorg. Chim. Acta..

[24] Kunchur H.S., Sonawane S.C., Saini P. (2023). Copper (I) Complexes of Amide Functionalized Bisphosphine: Proximity Enhanced Metal–Ligand Cooperativity and Its Catalytic Advantage in C (*sp*^3^)–H Bond Activation of Unactivated Cycloalkanes in Dehydrogenative Carboxylation Reactions. Inorg. Chem..

[25] Wan Q.T., Meng Y.L., Shuang Y. (2021). Copper Corrole as an Efficient Catalyst for Esterification of Allylic C (*sp*^3^)–H Bonds with Carboxylic Acids. Chin. J. Org. Chem..

