# Supplementary material for: Immobilized Copper Complexes on Coal-Bearing Kaolin for Catalyzing Allylic Ester Synthesis via C(sp3)–H Bond Activation
Source: Molecules. 2025 May 21;30(10):2232. doi: 10.3390/molecules30102232 (PMC12113897; doi:10.3390/molecules30102232)

Immobilized Copper Complexes on Coal-Bearing Kaolin for Catalyzing Allylic Ester Synthesis via C(sp^3)–H Bond Activation

Chun-Ling Zhang[†,1], Dao Su[†,1], Habuer Wang[1], Tegshi Muschin*[,1], Yun Wu[1], Yong-Sheng Bao[1], Huai-Yong Zhu*[,2]

[1] Inner Mongolia Key Laboratory of Green Catalysis, College of Chemistry and Environmental Science, Inner Mongolia Normal University, Inner Mongolia, Hohhot 010022, China; zwl15648207306@163.com (C.-L.Z.); 18404714387@163.com (D.S.); habur2004@163.com (H.W.); wy@imnu.edu.cn (Y.W.)

[2] School of Chemistry, Physics and Mechanical Engineering, Queensland University of Technology, 2 George Street, Brisbane 4001, Australia

* Correspondence: tegshi@imnu.edu.cn (T.M.); hy.zhu@qut.edu.au (H.-Y.Z.)

[†] These authors contributed equally to this work.

Experimental Section

Catalyst Preparation

Kaolin Pretreatment: Synthesis of [NH_2-Ph@CK]. To prepare the kaolin-based support, 1.0 g of coal-bearing kaolin was added to 30 mL of *n*-heptane in a beaker and stirred thoroughly. Then, 1.2 mmol each of 3-aminopropyltriethoxysilane and phenyltrimethoxysilane were added under continuous magnetic stirring. The mixture was heated in an oil bath at 100 °C for 24 hours. After cooling, the product was separated by centrifugation, washed twice with ethanol, twice with deionized water, and again with ethanol. The resulting solid was vacuum-dried at 50 °C overnight. The materials modified with only 3-aminopropyltriethoxysilane or phenyltrimethoxysilane were designated as NH_2@CK and Ph@CK, respectively.

Preparation of Cu/NH_2-Ph@CK Catalyst. The 3%Cu/NH_2-Ph@CK catalyst was synthesized using a lysine-assisted impregnation-reduction method. Specifically, 0.97 g of NH_2-Ph@CK was dispersed in 20 mL tetrahydrofuran and stirred for 20 minutes. Subsequently, 21 mL of $CuCl_2$ solution and 1 mL of 0.03 mol/L lysine solution were added, and the mixture was stirred for 30 minutes. The pH was adjusted to neutral using 0.1 mol/L NaOH solution. Then, 4.5 mL of freshly prepared 0.35 mol/L $NaBH_4$ solution was added dropwise over 10–15 minutes, followed by continuous stirring for 1 hour. After standing for 24 hours, the mixture was washed with deionized water until the pH reached 7, followed by a wash with anhydrous ethanol. The solid was separated by centrifugation and dried in an oven at 60 °C. The final catalyst was obtained by grinding. The 3%Cu/NH_2@CK and 3%Cu/Ph@CK catalysts were prepared using the same procedure, substituting NH_2@CK and Ph@CK as supports, respectively.

Characterization Techniques

Transmission Electron Microscopy (TEM): The catalyst's particle morphology and size distribution were observed using a JEP2100F electron microscope operated at 200 kV.

X-Ray Diffraction (XRD): Phase composition and crystallinity were examined using a Rigaku Ultima IV diffractometer with a Cu Kα source (λ = 1.5406 Å), operating at 40 mA and 40 kV. The scan was conducted from 2θ = 10°–80° at a rate of 8°/min.

X-Ray Photoelectron Spectroscopy (XPS): The chemical states of surface elements were analyzed using a Thermo Fisher ESCALAB 250Xi spectrometer, with Al Kα as the X-ray source. Binding energies were calibrated against the C1s peak at 284.80 eV.

N_2 Adsorption–Desorption Isotherms: Surface area measurements were performed on a Micromeritics ASAP-2020 instrument. Prior to analysis, the samples were degassed under vacuum at 80 °C.

Atomic Absorption Spectroscopy (AAS): Copper content in the catalyst was quantified using a TAS-990 atomic absorption spectrophotometer.

Nuclear Magnetic Resonance (NMR): Structural confirmation of reaction products was conducted using a Bruker Avance III 600 MHz NMR spectrometer. Deuterated chloroform ($CDCl_3$) containing 0.03% (v/v) tetramethylsilane (TMS) was used as the solvent and internal reference for both ^1H and ^{13}C NMR analyses.

Catalytic Activity Test

In a typical reaction, benzoic acid (0.3 mmol), cyclohexane (1 mL), di-tert-butyl peroxide (DTBP, 5.0 equiv), and 3%Cu/NH_2-Ph@CK catalyst (10 mol%) were added to a 25 mL oven-dried reaction tube. The mixture was heated to 120 °C and stirred magnetically for 24 hours. Upon completion, the reaction mixture was cooled to room temperature, and the product was purified via flash column chromatography using a 1:20 mixture of ethyl acetate/petroleum ether as the eluent. The product was identified by ^1H and ^{13}C NMR spectroscopy.

Catalyst Recycling Test

Following each reaction cycle, the catalyst was recovered by centrifugation and washed with distilled water until neutral pH was achieved. It was then rinsed with ethanol, vacuum-dried at 60 °C, and ground into a fine powder. The recycled catalyst was reused in subsequent reaction cycles under identical conditions.

Figure S1 ^{1}H NMR spectra of control experiments to check the conversion of cyclohexane to cyclohexene

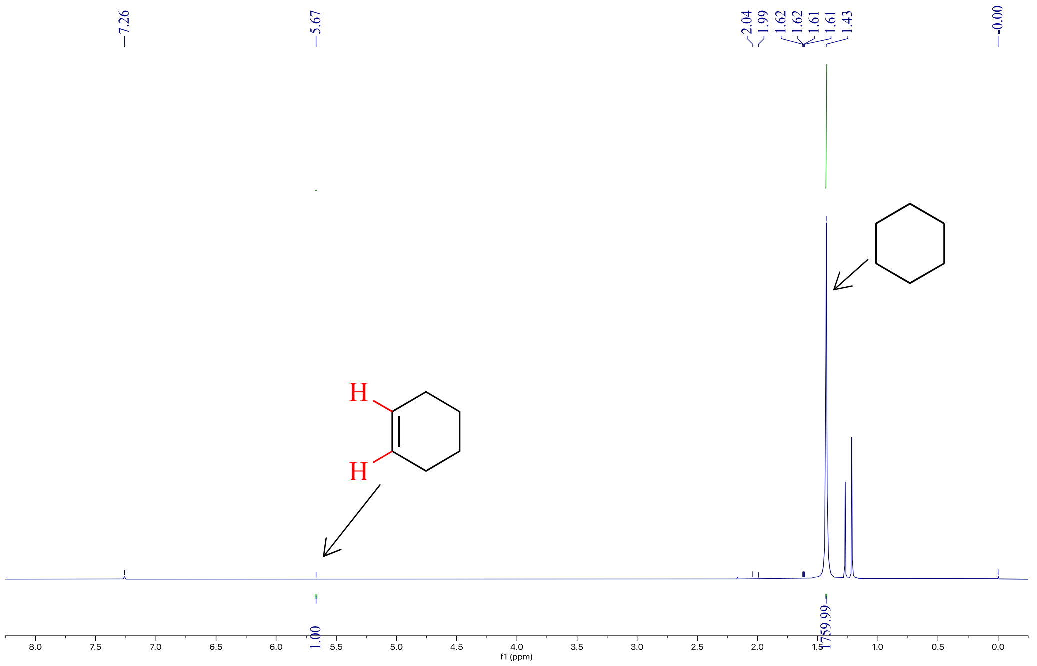

(a) Reaction conditions: cyclohexane (1 mL), 3%Cu/NH$_2$-Ph@CK (10 mol%), DTBP (5 equiv), no benzoic acid, 120 °C, 24 h. After the reaction, the hydrogen ratio of cyclohexene to cyclohexane is 1:1760.

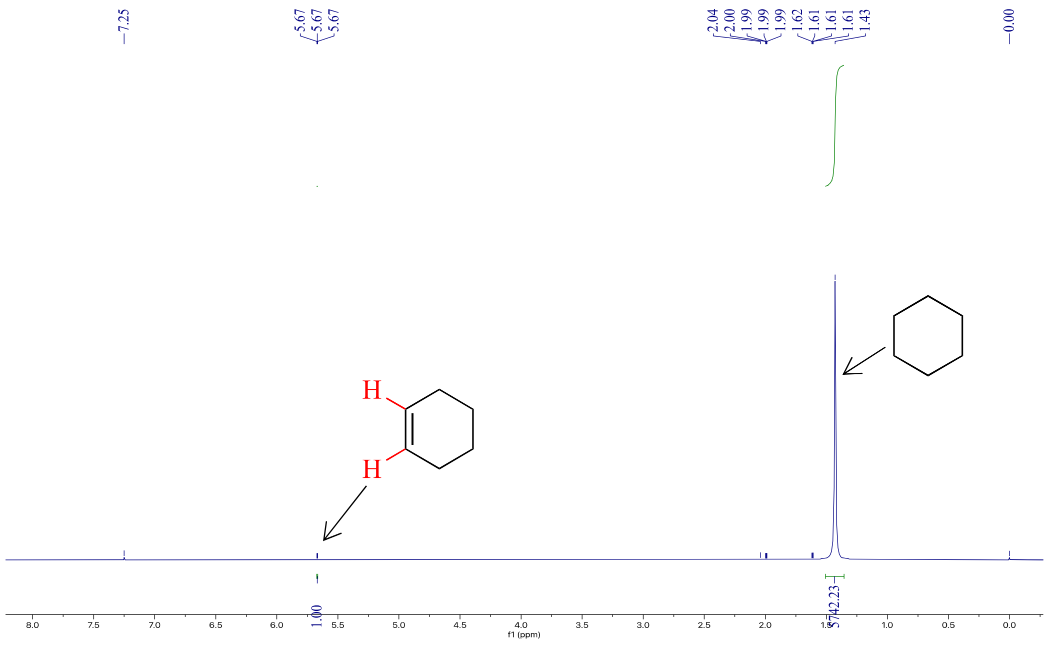

(b) Reaction conditions: cyclohexane (1 mL), 3%Cu/NH$_2$-Ph@CK (10 mol%), no benzoic acid and DTBP, 120 °C, 24 h. After the reaction, the hydrogen ratio of cyclohexene to cyclohexane is 1:5742.

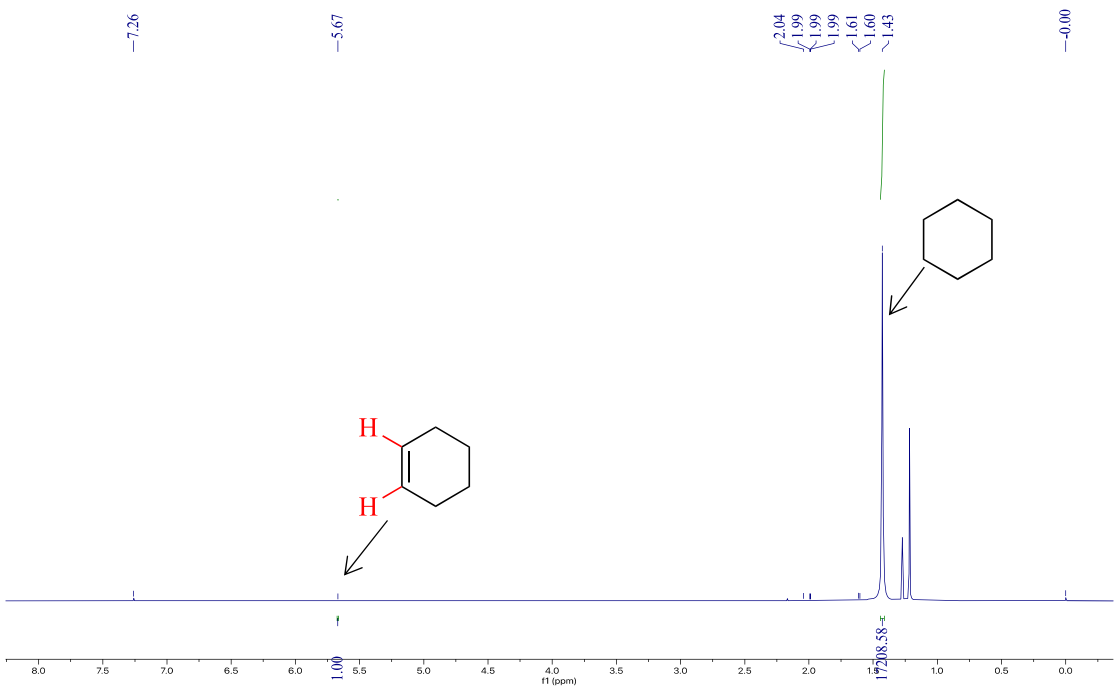

(c) Reaction conditions: cyclohexane (1 mL), DTBP(5 equiv), no benzoic acid and 3%Cu/NH_2-Ph@CK, 120 ℃, 24 h. After the reaction, the hydrogen ratio of cyclohexene to cyclohexane is 1:17209.

^{1}H NMR and ^{13}C NMR Spectra of the Products

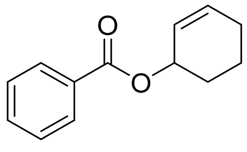

Cyclohex-2-en-1-yl benzoate (1a): (43.3 mg, 71% yield). ^{1}H NMR (600 MHz, $CDCl_3$) δ 8.06 – 8.05 (d, *J* = 7.0 Hz, 2H), 7.55 – 7.52 (t, *J* = 7.4 Hz, 1H), 7.43 – 7.41 (t, *J* = 7.7 Hz, 2H), 6.01 – 5.99 (m, 1H), 5.85 – 5.83 (d, *J* = 10.0 Hz, 1H), 5.51 (s, 1H), 2.15 – 2.12 (m, 1H), 2.06 – 2.05 (m, 1H), 1.98 – 1.83 (m, *J* = 9.8, 4.8 Hz, 1H), 1.89 – 1.83 (m, 2H), 1.72 – 1.70 (m, 1H). ^{13}C NMR (151 MHz, $CDCl_3$) δ 165.19, 131.79, 131.71, 129.80, 128.57, 127.24, 124.74, 67.57, 27.41, 23.94, 17.93.

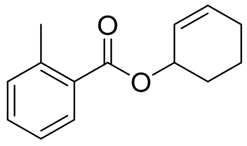

Cyclohex-2-en-1-yl 2-methylbenzoate (2a): (32.5 mg, 50% yield).^{1}H NMR (600 MHz, $CDCl_3$) δ 7.91 – 7.90 (d, *J* = 7.9 Hz, 1H), 7.38 (t, *J* = 7.5 Hz, 1H), 7.23 (t, *J* = 7.2 Hz, 2H), 6.01 – 5.99 (m, *J* = 10.1 Hz, 1H), 5.86 – 5.81 (d, 1H), 5.50 (s, 1H), 2.60 (s, 3H), 2.15 – 2.12 (m, *J* = 5.2 Hz, 1H), 2.06 – 1.96 (m, 2H), 1.91 – 1.81 (m, *J* = 41.8, 9.1, 5.1 Hz, 2H), 1.71 (m, *J* = 10.8, 2.7 Hz, 1H). ^{13}C NMR (151 MHz, $CDCl_3$) δ 167.40, 139.86, 132.74, 131.71, 131.58, 130.56, 130.35, 125.81, 125.65, 68.42, 28.47, 24.96, 21.75, 18.99.

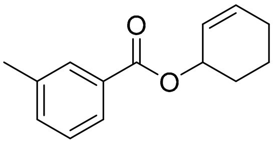

Cyclohex-2-en-1-yl 3-methylbenzoate (3a): (38.9 mg, 60% yield).^{1}H NMR (600 MHz, $CDCl_3$) δ 7.86 (d, 2H), 7.35 (d, *J* = 7.6 Hz, 1H), 7.31 – 7.30 (t, *J* = 7.5 Hz, 1H), 6.01 – 5.99 (m, *J* = 10.1 Hz, 1H), 5.84 – 5.83 (d, 1H), 5.50 (s, *J* = 1.8 Hz, 1H), 2.39 (s, 3H), 2.15 – 2.13 (m, 1H), 2.05 – 2.02 (d, *J* = 18.4 Hz, 1H), 1. 98 – 1.96 (m, *J* = 13.8 Hz, 1H), 1.89 – 1.85 (m, *J* = 13.6, 8.3 Hz, 2H), 1.71 – 1.69 (m, *J* = 8.5 Hz, 1H).^{13}C NMR (151 MHz, $CDCl_3$) δ 166.40, 138.04, 133.50, 132.75, 130.77, 130.11, 128.18, 126.77, 125.86, 68.54, 28.46, 24.98, 21.26, 19.02.

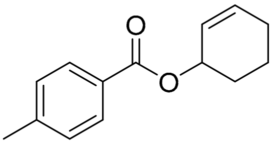

Cyclohex-2-en-1-yl 4-methylbenzoate (4a): (42.2 mg, 65% yield).^{1}H NMR (600 MHz, $CDCl_3$) δ 7.95 – 7.93 (d, *J* = 7.9 Hz, 2H), 7.22 –7.21 (d, *J* = 7.9 Hz, 2H), 6.00 – 5.98 (m, *J* = 10.1 Hz, 1H), 5.84 – 5.82 (d, 1H), 5.50 (s, 1H), 2.39 (s, 3H), 2.14 – 2.11 (m, 1H), 2.05 – 2.04 (m, 1H), 1.97 – 1.95 (m, 1H), 1.88 – 1.83 (m, 2H), 1.71 – 1.69 (m, 1H).^{13}C NMR (151 MHz, $CDCl_3$) δ 166.29, 143.34, 132.67, 129.64, 128.98, 128.12, 125.94, 68.39, 28.48, 24.99, 21.63, 19.02.

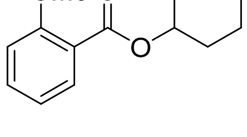

Cyclohex-2-en-1-yl 2-methoxybenzoate (5a): (37.6 mg, 54% yield).^{1}H NMR (600 MHz, $CDCl_3$) δ 7.79 – 7.77 (d, 1H), 7.46 – 7.43 (t, J = 7.9 Hz, 1H), 6.97 – 6.95 (m, 2H), 5.99 – 5.98 (m, J = 10.1 Hz, 1H), 5.86 – 5.85 (d, J = 2.2 Hz, 1H), 5.50 (s, 1H), 3.89 (s, 3H), 2.13 – 2.10 (m, 1H), 2.05 – 1.94 (m, 2H), 1.90 – 1.81 (m, 2H), 1.70 – 1.68 (m, J = 13.3, 3.9 Hz, 1H). ^{13}C NMR (151 MHz, $CDCl_3$) δ 165.78, 159.20, 133.22, 132.68, 131.48, 125.90, 120.85, 120.09, 112.11, 68.36, 56.02, 28.44, 24.98, 18.95.

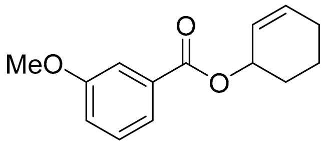

Cyclohex-2-en-1-yl 3-methoxybenzoate (6a): (41.8 mg, 60% yield).^{1}H NMR (600 MHz, $CDCl_3$) δ 7.65 – 7.64 (d, J = 7.7 Hz, 1H), 7.58 (s, J = 1.4 Hz, 1H), 7.35 – 7.32 (t, J = 7.9 Hz, 1H), 7.10 – 7.08 (d, J = 8.4, 2.7 Hz, 1H), 6.01 – 5.99 (m, 1H), 5.84 – 5.82 (d, J = 9.9, 2.2 Hz, 1H), 5.51 (s, J = 3.6 Hz, 1H), 3.85 (s, 3H), 2.13 (m, J = 8.0 Hz, 1H), 2.06 – 1.96 (m, 2H), 1.90 – 1.82 (m, 2H), 1.72 – 1.69 (m, 1H). ^{13}C NMR (151 MHz, $CDCl_3$) δ 166.09, 159.55, 132.83, 132.17, 129.28, 125.74, 122.01, 119.16, 114.19, 68.75, 55.44, 28.42, 24.96, 18.97.

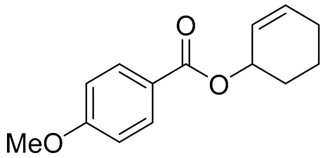

Cyclohex-2-en-1-yl 4-methoxybenzoate (7a): (44.6 mg, 64% yield).^{1}H NMR (600 MHz, $CDCl_3$) δ 8.01 – 8.00 (d, 2H), 6.91 – 6.90 (d, 2H), 6.00 – 5.98 (m, 1H), 5.84 – 5.81 (m, 1H), 5.49 – 5.47 (s, 1H), 3.84 (d, J = 2.0 Hz, 3H), 2.14 – 2.11 (m, J = 5.2 Hz, 1H), 2.05 – 1.95 (m, 2H), 1.89 – 1.81 (m, 2H), 1.71 – 1.69 (t, 1H). ^{13}C NMR (151 MHz, $CDCl_3$) δ 165.97, 163.27, 132.59, 131.60, 126.03, 123.29, 113.52, 68.26, 55.40, 28.51, 24.99, 19.03.

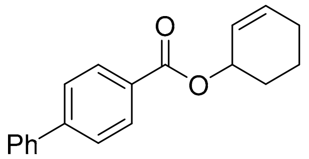

Cyclohex-2-en-1-yl [1,1'-biphenyl]-4-carboxylate (8a): (39.2 mg, 47% yield).^{1}H NMR (600 MHz, $CDCl_3$) δ 8.13 – 8.11 (d, 2H), 7.65 – 7.61 (dd, 4H), 7.47 – 7.44 (t, J = 7.5 Hz, 2H), 7.40 – 7.37 (t, J = 7.3 Hz, 1H), 6.02 – 6.01 (m, J = 10.1 Hz, 1H), 5.87 – 5.85 (d, 1H), 5.53 (s, J = 4.5 Hz, 1H), 2.17 – 2.12 (m, 1H), 2.07 – 1.97 (m, 2H), 1.92 – 1.85 (m, 2H), 1.73 – 1.71 (m, 1H). ^{13}C NMR (151 MHz, $CDCl_3$) δ 166.11, 145.50, 140.12, 132.83, 130.12, 129.58, 128.91, 128.08, 127.28, 126.97, 125.81, 68.64, 28.47, 24.98, 19.00.

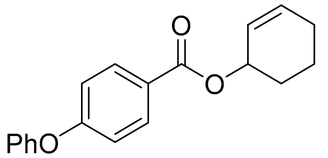

Cyclohex-2-en-1-yl 4-phenoxybenzoate (9a): (55.6 mg, 63% yield).^{1}H NMR (600 MHz, $CDCl_3$) δ 8.03 – 8.01 (d, J = 8.8 Hz, 2H), 7.38 – 7.36 (t, 2H), 7.19 – 7.16 (t, 1H), 7.06 – 7.04 (d, 2H), 6.99 – 6.97 (d, J = 8.8 Hz, 2H), 6.01 – 5.97 (m, 1H), 5.84 – 5.81 (m, J = 10.1, 3.7 Hz, 1H),5.50 – 5.49 (m, J = 5.3 Hz, 1H), 2.15 – 2.11 (m, 1H), 2.05 – 1.94 (m, 2H), 1.88 – 1.81 (m, 2H), 1.71 – 1.70

(m, J = 5.1 Hz, 1H).^{13}C NMR (151 MHz, $CDCl_3$) δ 165.69, 161.62, 155.81, 132.73, 131.69, 129.98, 125.86, 125.23, 124.36, 119.94, 117.37, 68.47, 28.47, 24.96, 18.98.

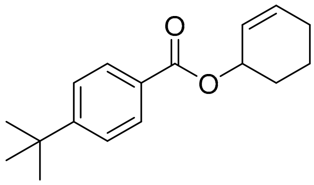

Cyclohex-2-en-1-yl 4-(tert-butyl) benzoate (10a): (37.2 mg, 48% yield). ^{1}H NMR (600 MHz, $CDCl_3$) δ 7.99 (d, J = 7.3 Hz, 2H), 7.45 – 7.43 (d, 2H), 6.00 – 5.98 (m, J = 12.3 Hz, 1H), 5.84 – 5.82 (d, 1H), 5.50 (s, J = 3.4 Hz, 1H), 2.15 – 2.10 (d, J = 16.4, 5.1 Hz, 1H), 2.06 – 1.94 (m, 2H), 1.89 – 1.81 (m, 2H), 1.72 – 1.67 (m, 1H), 1.33 (d, 9H). ^{13}C NMR (151 MHz, $CDCl_3$) δ 166.23, 156.37, 132.63, 129.48, 128.07, 125.94, 125.23, 68.34, 35.05, 31.14, 28.49, 24.98, 19.00.

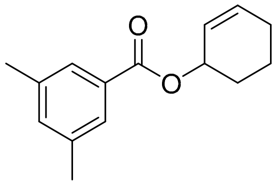

Cyclohex-2-en-1-yl 3,5-dimethylbenzoate (11a): (40.0 mg, 58% yield). ^{1}H NMR (600 MHz, $CDCl_3$) δ 7.66 (s, 2H), 7.17 (s, 1H), 6.01 – 5.99 (m, J = 10.1 Hz, 1H), 5.84 – 5.82 (d, J = 12.0 Hz, 1H), 5.50 (s, 1H), 2.35 (s, 6H), 2.16 – 2.11 (m, 1H), 2.07 – 1.96 (m, 2H), 1.90 – 1.82 (m, J = 16.8, 9.4 Hz, 2H), 1.72 – 1.68 (m, J = 11.0, 6.6 Hz, 1H). ^{13}C NMR (151 MHz, $CDCl_3$) δ 166.58, 137.92, 134.40, 132.70, 130.70, 127.30, 125.93, 68.48, 28.47, 24.99, 21.15, 19.06.

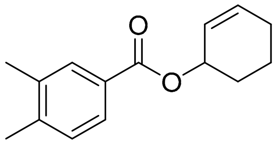

Cyclohex-2-en-1-yl 3,4-dimethylbenzoate (12a): (38.6 mg, 56% yield).^{1}H NMR (600 MHz, $CDCl_3$) δ 7.71 – 7.69 (d, 1H), 7.56 (s, J = 1.5 Hz, 1H), 6.88 – 6.87 (d, J = 8.4 Hz, 1H), 6.01 – 5.99 (m, J = 10.1 Hz, 1H), 5.84 – 5.82 (d, J = 12.7 Hz, 1H), 5.50 (s, J = 7.6 Hz, 1H), 3.94 (s, 6H), 2.07 – 1.96 (m, 2H), 1.89 – 1.82 (m, 2H), 1.74 – 1.68 (m, J = 5.7 Hz, 2H). ^{13}C NMR (151 MHz, $CDCl_3$) δ 166.03, 152.91, 148.63, 132.64, 126.01, 123.58, 123.38, 112.12, 110.23, 68.54, 56.03, 28.50, 24.98, 19.09.

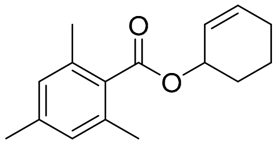

Cyclohex-2-en-1-yl 2,4,6-trimethylbenzoate (13a): (39.6 mg, 54% yield). ^{1}H NMR (600 MHz, $CDCl_3$) δ 6.83 (s, 2H), 5.99 – 5.98 (m, J = 10.2 Hz, 1H), 5.86 – 5.83 (d, J = 12.9 Hz, 1H), 5.55 – 5.51 (s, 1H), 2.30 (s, 6H), 2.26 (s, 3H), 2.09 – 2.07 (m, 1H), 2.04 – 1.96 (m, 2H), 1.89 – 1.86 (m, 1H), 1.79 – 1.76 (m, 1H), 1.69 – 1.66 (m, J = 14.1 Hz, 1H). ^{13}C NMR (151 MHz, $CDCl_3$) δ 169.80, 139.04, 134.90, 132.89, 131.36, 128.35, 125.50, 68.68, 28.40, 24.90, 21.10, 19.67, 18.88.

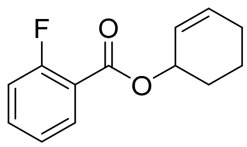

Cyclohex-2-en-1-yl 2-fluorobenzoate (14a): (40.3 mg, 61% yield).^{1}H NMR (600 MHz, $CDCl_3$) δ 7.93 – 7.91 (t, *J* = 7.5 Hz, 1H), 7.51 – 7.48 (q, *J* = 6.8 Hz, 1H), 7.20 – 7.18 (t, *J* = 7.6 Hz, 1H), 7.13 – 7.10 (t, 1H), 6.02 – 6.00 (m, 1H), 5.87 – 5.84 (d, 1H), 5.52 – 5.51 (s, 1H), 2.16 – 2.12 (m, *J* = 18.1, 6.0 Hz, 1H), 2.05 – 1.94 (m, 2H), 1.92 – 1.82 (m, 2H), 1.72 – 1.68 (m, 1H). ^{13}C NMR (151 MHz, $CDCl_3$) δ 164.05, 164.03, 162.85, 161.13, 134.22, 134.16, 133.13, 132.05, 125.46, 123.86, 123.84, 119.49, 119.42, 117.01, 116.86, 69.06, 28.38, 24.96, 18.83.

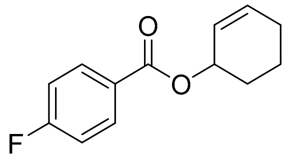

Cyclohex-2-en-1-yl 4-fluorobenzoate (15a): (43.6 mg, 66% yield). ^{1}H NMR (600 MHz, $CDCl_3$) δ 8.00 – 7.97 (q, 2H), 7.03 –7.00 (t, *J* = 8.2 Hz, 2H), 5.94 – 5.92 (m, *J* = 10.1 Hz, 1H), 5.76 – 5.75 (d, *J* = 3.1 Hz, 1H), 5.42 (s, *J* = 2.8 Hz, 1H), 2.07 – 2.04 (m, *J* = 19.7 Hz, 1H), 1.98 – 1.87 (m, 2H), 1.81 – 1.74 (m, 2H), 1.64 – 1.63 (m, *J* = 4.6 Hz, 1H).^{13}C NMR (151 MHz, $CDCl_3$) δ 166.55, 165.27, 164.87, 132.98, 132.16, 132.10, 127.10, 127.08, 125.65, 115.47, 115.32, 68.81, 28.44, 24.97, 18.96.

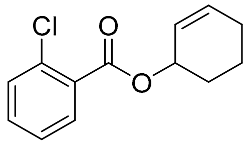

Cyclohex-2-en-1-yl 2-chlorobenzoate (16a): (41.2 mg, 58% yield).^{1}H NMR (600 MHz, $CDCl_3$) δ 7.81 – 7.79 (d, *J* = 7.7 Hz, 1H), 7.44 – 7.38 (m, 2H), 7.31 – 7.28 (t, *J* = 7.5 Hz, 1H), 6.03 – 6.01 (m, *J* = 10.3 Hz, 1H), 5.87 – 5.85 (d, *J* = 9.9 Hz, 1H), 5.53 (s, *J* = 4.6 Hz, 1H), 2.15 – 2.11 (dd, *J* = 18.1, 3.9 Hz, 1H), 2.05 – 1.92 (m, 3H), 1.84 – 1.82 (m, *J* = 4.0 Hz, 1H), 1.71 – 1.70 (m, *J* = 6.2, 2.9 Hz, 1H).^{13}C NMR (151 MHz, $CDCl_3$) δ 165.49, 133.53, 133.22, 132.25, 131.26, 130.95, 130.90, 126.52, 125.30, 69.44, 28.31, 24.93, 18.84.

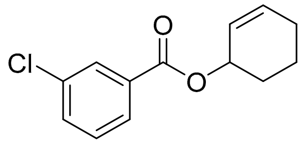

Cyclohex-2-en-1-yl 3-chlorobenzoate (17a): (43.3 mg, 61% yield).^{1}H NMR (600 MHz, $CDCl_3$) δ 8.02 (s, 1H), 7.94 (d, *J* = 7.8 Hz, 1H), 7.56 – 7.49 (m, 1H), 7.38 (t, *J* = 7.9 Hz, 1H), 6.02 (d, *J* = 10.2 Hz, 1H), 5.82 (d, *J* = 8.1 Hz, 1H), 5.51 (d, *J* = 3.5 Hz, 1H), 2.15 (dd, *J* = 18.2, 4.1 Hz, 1H), 2.07 – 1.94 (m, 2H), 1.90 – 1.81 (m, 2H), 1.74 – 1.68 (m, 1H). ^{13}C NMR (151 MHz, $CDCl_3$) δ 165.02, 134.46, 133.19, 132.78, 132.60, 129.67, 129.61, 127.76, 125.41, 69.16, 28.37, 24.94, 18.91.

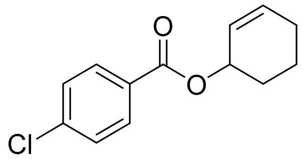

Cyclohex-2-en-1-yl 4-chlorobenzoate (18a): (45.5 mg, 64% yield).^{1}H NMR (600 MHz, $CDCl_3$) δ 7.99 – 7.98 (d, *J* = 7.0 Hz, 2H), 7.40 – 7.39 (d, *J* = 7.0 Hz, 2H), 6.02 – 6.00 (m, *J* = 10.2 Hz, 1H), 5.83 – 5.81 (d, *J* = 10.3 Hz, 1H), 5.50 (s, *J* = 3.8 Hz, 1H), 2.16 – 2.12 (m, 1H), 2.06 – 1.95 (m, 2H), 1.89 – 1.82 (m, 2H), 1.71 (m, *J* = 5.3 Hz, 1H).^{13}C NMR (151 MHz, $CDCl_3$) δ 165.33, 139.17, 133.03, 130.99, 129.29, 128.60, 125.54, 68.93, 28.39, 24.94, 18.92.

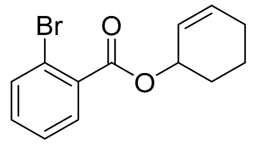

Cyclohex-2-en-1-yl 2-bromobenzoate (19a): (42.2 mg, 50% yield). ^{1}H NMR (600 MHz, $CDCl_3$) δ 7.77 – 7.76 (d, *J* = 7.6 Hz, 1H), 7.64 – 7.63 (d, *J* = 8.0 Hz, 1H), 7.36 – 7.34 (t, *J* = 7.5 Hz, 1H), 7.31 – 7.29 (t, *J* = 7.6 Hz, 1H), 6.03 – 6.01 (m, *J* = 10.1 Hz, 1H), 5.86 – 5.86 (d, *J* = 6.5 Hz, 1H), 5.53 (s, *J* = 3.6 Hz, 1H), 2.15 – 2.11 (dd, *J* = 18.9, 4.6 Hz, 1H), 2.05 – 1.93 (m, 3H), 1.85 – 1.83 (m, *J* = 8.9, 4.4 Hz, 1H), 1.72 – 1.69 (m, 1H). ^{13}C NMR (151 MHz, $CDCl_3$) δ 165.99, 134.21, 133.23, 132.96, 132.26, 131.18, 127.11, 125.26, 121.48, 69.58, 28.27, 24.92, 18.85.

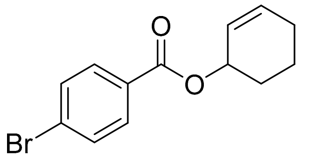

Cyclohex-2-en-1-yl 4-bromobenzoate (20a): (54.0 mg, 64% yield). ^{1}H NMR (600 MHz, $CDCl_3$) δ 7.91 – 7.90 (d, 2H), 7.57 – 7.55 (d, 2H), 6.02 – 6.00 (m, *J* = 10.2 Hz, 1H), 5.83 – 5.81 (d, 1H), 5.50 (s, 1H), 2.16 – 2.12 (dd, *J* = 17.8, 3.7 Hz, 1H), 2.06 – 1.95 (m, 2H), 1.88 – 1.82 (m, 2H), 1.72 – 1.69 (m, *J* = 9.4, 4.7 Hz, 1H). ^{13}C NMR (151 MHz, $CDCl_3$) δ 165.45, 133.06, 131.60, 131.14, 129.74, 127.82, 125.52, 68.97, 28.39, 24.94, 18.92.

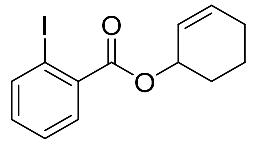

Cyclohex-2-en-1-yl 2-iodobenzoate (21a): (35.4 mg, 36% yield).^{1}H NMR (600 MHz, $CDCl_3$) δ 7.98 – 7.96 (d, 1H), 7.79 – 7.77 (dd, *J* = 7.8, 1.7 Hz, 1H), 7.340 – 7.37 (t, *J* = 8.2 Hz, 1H), 7.15 – 7.12 (m, *J* = 7.7, 1.7 Hz, 1H), 6.03 – 6.02 (m, *J* = 10.1 Hz, 1H), 5.88 – 5.86 (m, *J* = 10.1, 1.9 Hz, 1H), 5.53 (d, *J* = 5.2 Hz, 1H), 2.15 – 2.12 (m, *J* = 18.3, 4.0 Hz, 1H), 2.06 – 1.935 (m, 3H), 1.86 – 1.84 (m, *J* = 9.7, 3.6 Hz, 1H), 1.72 – 1.69 (m, *J* = 5.7, 3.4 Hz, 1H). ^{13}C NMR (151 MHz, $CDCl_3$) δ 166.35, 141.17, 135.85, 133.20, 132.36, 130.84, 127.85, 125.29, 93.90, 69.68, 28.25, 24.92, 18.87.

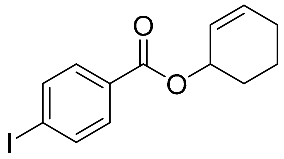

Cyclohex-2-en-1-yl 4-iodobenzoate (22a): (53.1 mg, 54% yield).^{1}H NMR (600 MHz, $CDCl_3$) δ 8.37 (s, 1H), 8.02 – 8.00 (d, *J* = 7.8 Hz, 1H), 7.87 – 7.86 (d, *J* = 7.8 Hz, 1H), 7.18 – 7.16 (t, *J* = 7.8 Hz, 1H), 6.03 – 6.01 (m, *J* = 10.2 Hz, 1H), 5.83 – 5.81 (d, 1H), 5.50 (s, 1H), 2.16 – 2.13 (dd, *J* = 18.2 Hz, 1H), 2.06 – 1.95 (m, 2H), 1.89 – 1.83 (m, 2H), 1.72 – 1.70 (m, *J* = 5.1 Hz, 1H). ^{13}C NMR (151 MHz, $CDCl_3$) δ 164.69, 141.57, 138.41, 133.13, 132.73, 129.96, 128.78, 125.43, 93.75, 69.15, 28.35, 24.93, 18.92.

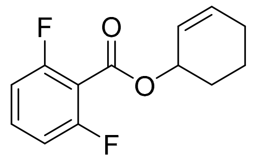

Cyclohex-2-en-1-yl 2,6-difluorobenzoate (23a): (42.5 mg, 50% yield). ^{1}H NMR (600 MHz, $CDCl_3$) δ 7.41 – 7.36 (m, *J* = 13.3, 7.5 Hz, 1H), 6.95 – 6.92 (t, *J* = 8.6 Hz, 2H), 6.04 – 6.02 (m, *J* = 10.3 Hz, 1H), 5.86 – 5.83 (d, 1H), 5.56 (s, 1H), 2.15 – 2.11 (dd, *J* = 22.8 Hz, 1H), 2.05 – 1.93 (m, 3H), 1.81 – 1.80 (m, *J* = 5.0 Hz, 1H), 1.69 (m, 1H). ^{13}C NMR (151 MHz, $CDCl_3$) δ 161.49, 161.45, 161.28, 159.79, 159.75, 133.58, 132.45, 132.38, 132.31, 124.97, 112.03, 112.00, 111.96, 111.89, 111.86, 111.84, 111.72, 70.05, 28.27, 24.90, 18.66.

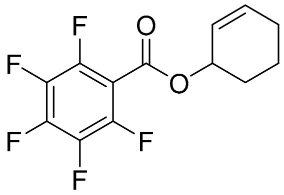

Cyclohex-2-en-1-yl 2,3,4,5,6-pentafluorobenzoate (24a): (37.7 mg, 43% yield).^{1}H NMR (600 MHz, $CDCl_3$) δ 6.08 – 6.05 (m, 1H), 5.84 – 5.83 (m, 1H), 5.55 – 5.54 (d, *J* = 4.9 Hz, 1H), 2.16 – 2.13 (dd, *J* = 18.4, 4.4 Hz, 1H), 2.07 – 2.03 (m, 1H), 1.97 – 1.92 (m, *J* = 16.7, 11.0, 5.9 Hz, 2H), 1.81 – 1.79 (m, 1H), 1.73 – 1.69 (m, *J* = 7.7, 4.0 Hz, 1H). ^{13}C NMR (151 MHz, $CDCl_3$) δ 158.77, 146.21, 144.47, 143.97, 142.25, 134.34, 124.37, 109.02, 71.23, 28.26, 24.93, 18.54.

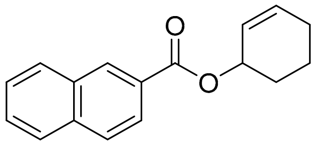

Cyclohex-2-en-1-yl 2-naphthoate (25a): (49.2 mg, 65% yield). ^{1}H NMR (600 MHz, $CDCl_3$) δ 8.61 (s, 1H), 8.08 – 8.07 (dd, *J* = 8.6, 1.7 Hz, 1H), 7.95 – 7.94 (d, *J* = 8.4 Hz, 1H), 7.87 – 7.85 (d, *J* = 8.7 Hz, 2H), 7.58 – 7.51 (m, 2H), 6.04 – 6.02 (m, 1H), 5.90 – 5.88 (m, *J* = 10.1 Hz, 1H), 5.59 – 5.58 (d, *J* = 1.6 Hz, 1H), 2.18 – 2.15 (m, *J* = 18.2 Hz, 1H), 2.08 – 2.00 (m, 2H), 1.95 – 1.88 (m, 2H), 1.75 – 1.73 (m, 1H). ^{13}C NMR (151 MHz, $CDCl_3$) δ 166.37, 135.48, 132.87, 132.52, 130.94, 129.32, 128.09, 128.07, 128.02, 127.74, 126.54, 125.85, 125.36, 68.80, 28.50, 25.00, 19.05.

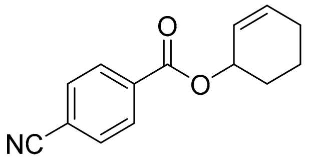

Cyclohex-2-en-1-yl ethyl 4-cyanobenzoate (26a): (13.6 mg, 20% yield).^{1}H NMR (600 MHz, $CDCl_3$) δ 8.16 – 8.14 (d, *J* = 8.0 Hz, 2H), 7.75 – 7.73 (d, *J* = 8.0 Hz, 2H), 6.05 – 6.03 (d, *J* = 10.2 Hz, 1H), 5.83 – 5.82 (d, *J* = 10.1 Hz, 1H), 5.52 (s, *J* = 3.8 Hz, 1H), 2.17 – 2.14 (d, *J* = 17.4 Hz, 1H), 2.08 – 1.97 (m, 2H), 1.91 – 1.83 (m, *J* = 46.8 Hz, 2H), 1.73 (m, 1H). ^{13}C NMR (151 MHz, $CDCl_3$) δ 164.57, 134.69, 133.54, 132.15, 130.13, 125.08, 118.05, 116.23, 69.66, 28.32, 24.92, 18.83.

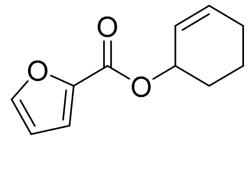

Cyclohex-2-en-1-yl furan-2-carboxylate (29a): (11.5 mg, 20% yield).^{1}H NMR (600 MHz, $CDCl_3$) δ 7.57 (s, 1H), 7.18 – 7.17 (d, *J* = 3.5 Hz, 1H), 6.50 (t, 1H), 6.02 – 6.00 (m, *J* = 10.1 Hz, 1H), 5.81 – 5.79 (d, 1H), 5.50 (s, 1H), 2.15 – 2.11 (dd, 1H), 2.05 – 1.95 (m, 2H), 1.88 – 1.81 (m, 2H), 1.70 – 1.68 (m, 1H). ^{13}C NMR (151 MHz, $CDCl_3$) δ 158.51, 146.13, 145.13, 133.20, 125.43, 117.67, 111.73, 68.85, 28.39, 24.91, 18.92.

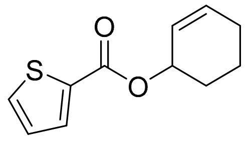

Cyclohex-2-en-1-yl thiophene-2-carboxylate (30a): (26.3 mg, 42% yield). ^{1}H NMR (600 MHz, $CDCl_3$) δ 7.80 – 7.79 (dd, *J* = 4.2 Hz, 1H), 7.54 – 7.53 (dd, 1H), 7.10 – 7.08 (t, 1H), 6.01 – 5.99 (m, *J* = 11.0 Hz, 1H), 5.83 – 5.81 (m, 1H), 5.47 – 5.46 (d, *J* = 5.3 Hz, 1H), 2.16 – 2.11 (m, 1H), 2.05 (m, *J* = 1.9 Hz, 1H), 1.98 – 1.94 (m, 1H), 1.89 – 1.81 (m, 2H), 1.70 – 1.67 (m, 1H). ^{13}C NMR (151 MHz, $CDCl_3$) δ 161.96, 134.54, 133.19, 133.00, 132.12, 127.63, 125.57, 68.99, 28.40, 24.93, 18.91.

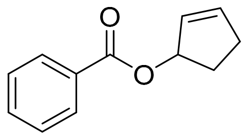

Cyclopent-2-en-1-yl benzoate (1b): (26.0 mg, 46% yield).^{1}H NMR (600 MHz, $CDCl_3$) δ 8.04 – 8.03 (d, *J* = 7.8 Hz, 2H), 7.54 – 7.52 (t, *J* = 7.5 Hz, 1H), 7.43 – 7.41 (t, *J* = 7.6 Hz, 2H), 6.16 – 6.15 (d, 1H), 5.957 – 5.94 (d, 2H), 2.61 – 2.56 (m, 1H), 2.42 – 2.37 (m, 2H), 1.99 – 1.96 (m, 1H). ^{13}C NMR (151 MHz, $CDCl_3$) δ 166.57, 137.66, 132.73, 130.74, 129.58, 129.41, 128.27, 81.13, 31.19, 29.92.

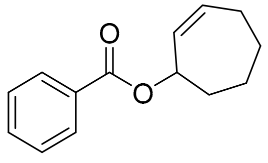

Cyclooct-2-en-1-yl benzoate (1c): (33.1 mg, 51% yield).^{1}H NMR (600 MHz, $CDCl_3$) δ 8.07 – 8.05 (dd, 2H), 7.56 – 7.54 (t, *J* = 7.4 Hz, 1H), 7.45 – 7.42 (t, *J* = 7.8 Hz, 2H), 5.89 – 5.86 (m, *J* = 11.8, 6.9 Hz, 1H), 5.81 – 5.79 (d, *J* = 11.8 Hz, 1H), 5.67 – 5.65 (d, *J* = 11.6 Hz, 1H), 2.27 – 2.24 (m, 1H), 2.15 (m, 1H), 2.00 – 1.98 (m, *J* = 11.1 Hz, 2H), 1.84 – 1.82 (m, *J* = 10.9 Hz, 1H), 1.74 – 1.73 (m, *J* = 9.2 Hz, 2H), 1.51 – 1.47 (m, 1H). ^{13}C NMR (151 MHz, $CDCl_3$) δ 165.90, 133.43, 132.79, 132.00, 130.78, 129.61, 128.30, 74.70, 32.83, 28.55, 26.73, 26.51.

References:

1. Su D.; Muschin T.; Wu Y. Direct esterification of allylic C (sp^3)–H via iron nanoparticle–loaded kaolin-catalyzed cross dehydrogenative coupling. *Green Chem. Lett. Rev.s*, **2024**, *17(1)*, 2315130.

2. Wan Q T.; Meng Y L.;Shuang Y. Copper Corrole as an Efficient Catalyst for Esterification of Allylic C (sp^3)–H Bonds with Carboxylic Acids. *Chinese. J. Org. Chem.*, **2021**, *41(7)*, 2875.

^{1}H NMR of cyclohex-2-en-1-yl benzoate (**1a**)

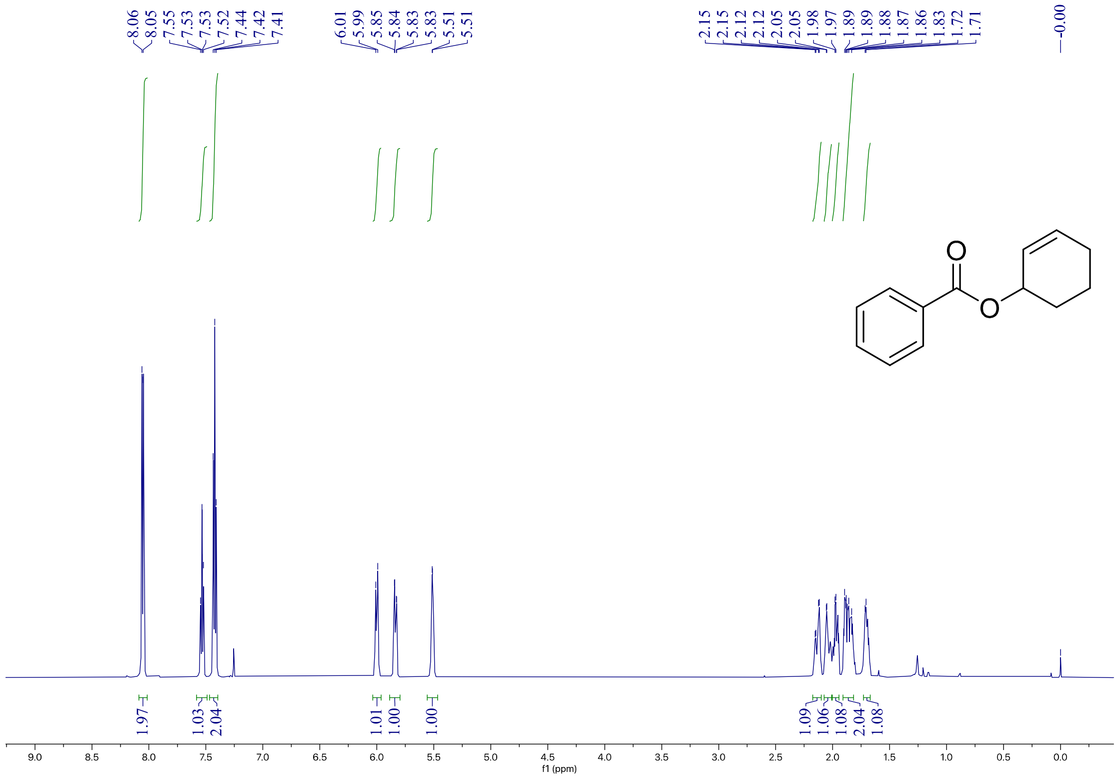

^{13}C NMR of cyclohex-2-en-1-yl benzoate (**1a**)

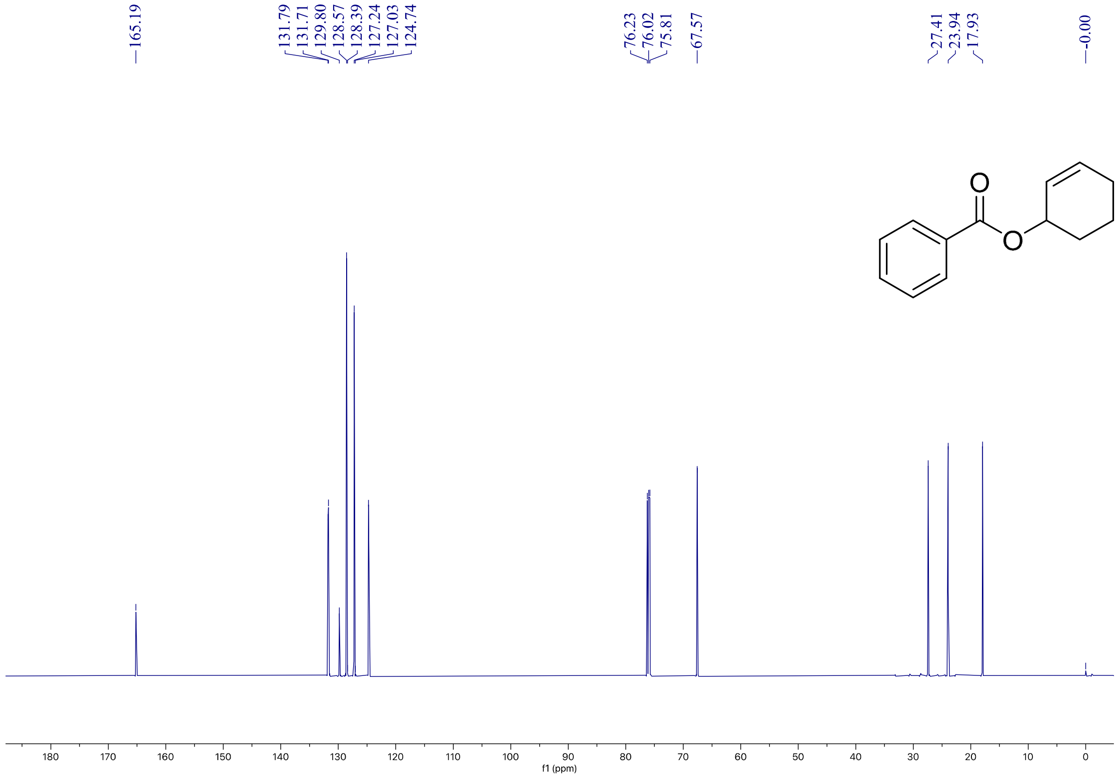

^{1}H NMR of cyclohex-2-en-1-yl 2-methylbenzoate (**2a**)

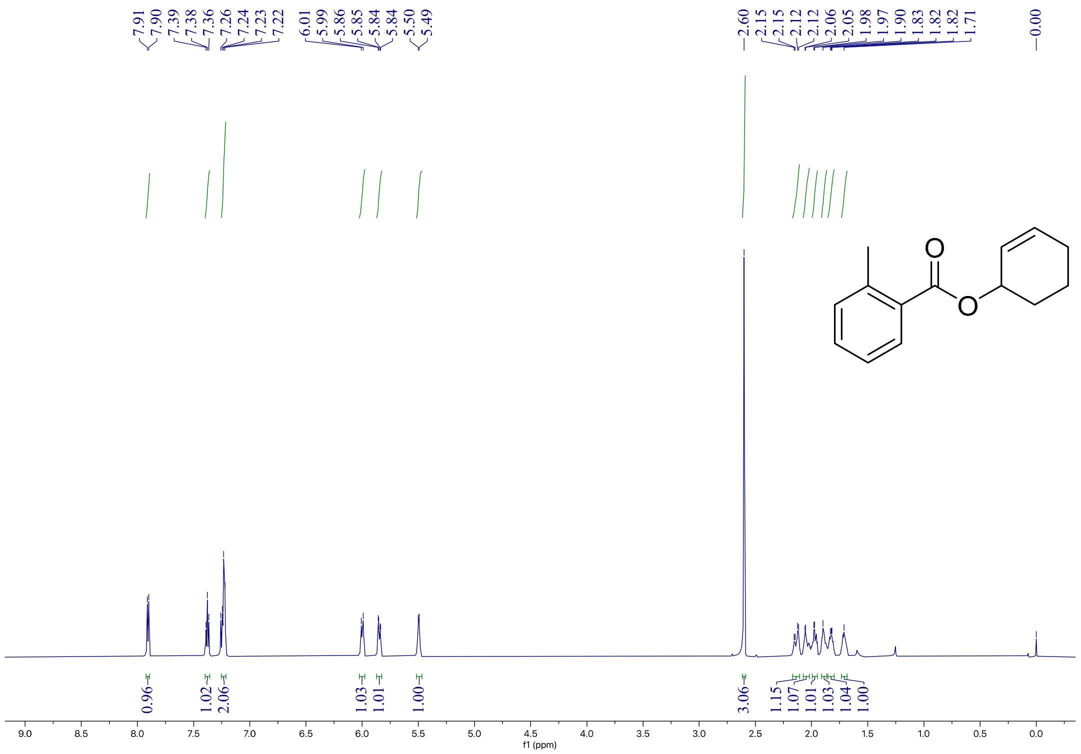

^{13}C NMR of cyclohex-2-en-1-yl 2-methylbenzoate (**2a**)

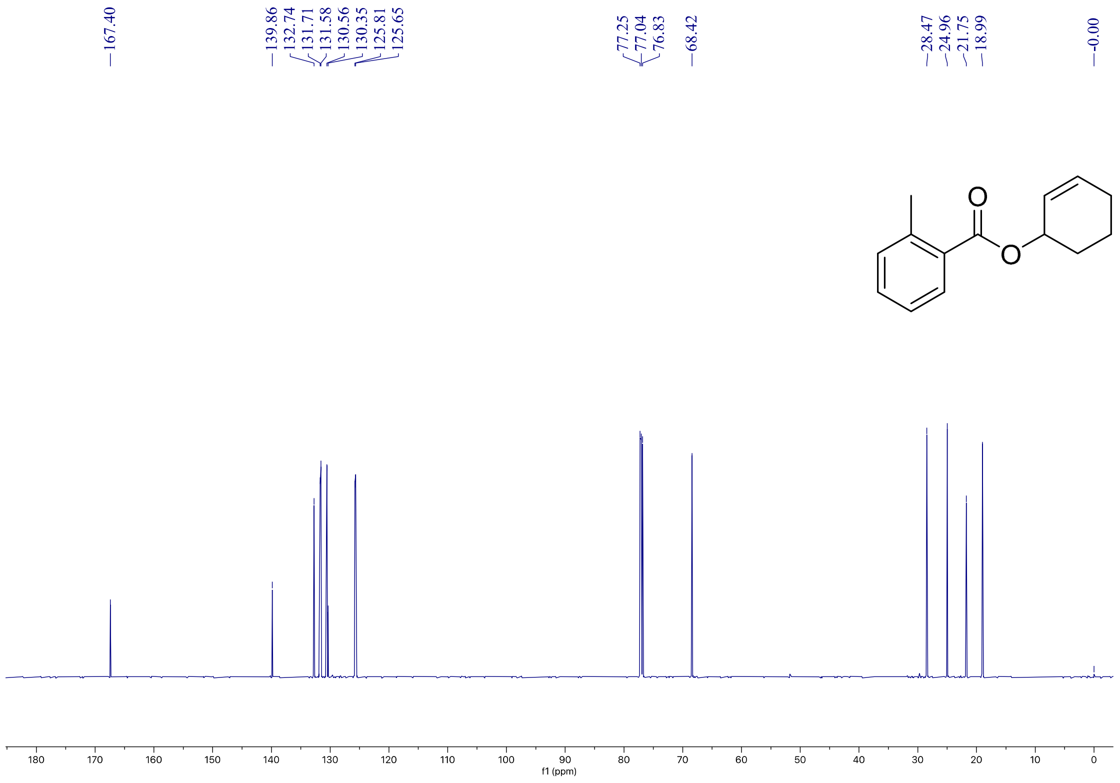

^{1}H NMR of cyclohex-2-en-1-yl 3-methylbenzoate (**3a**)

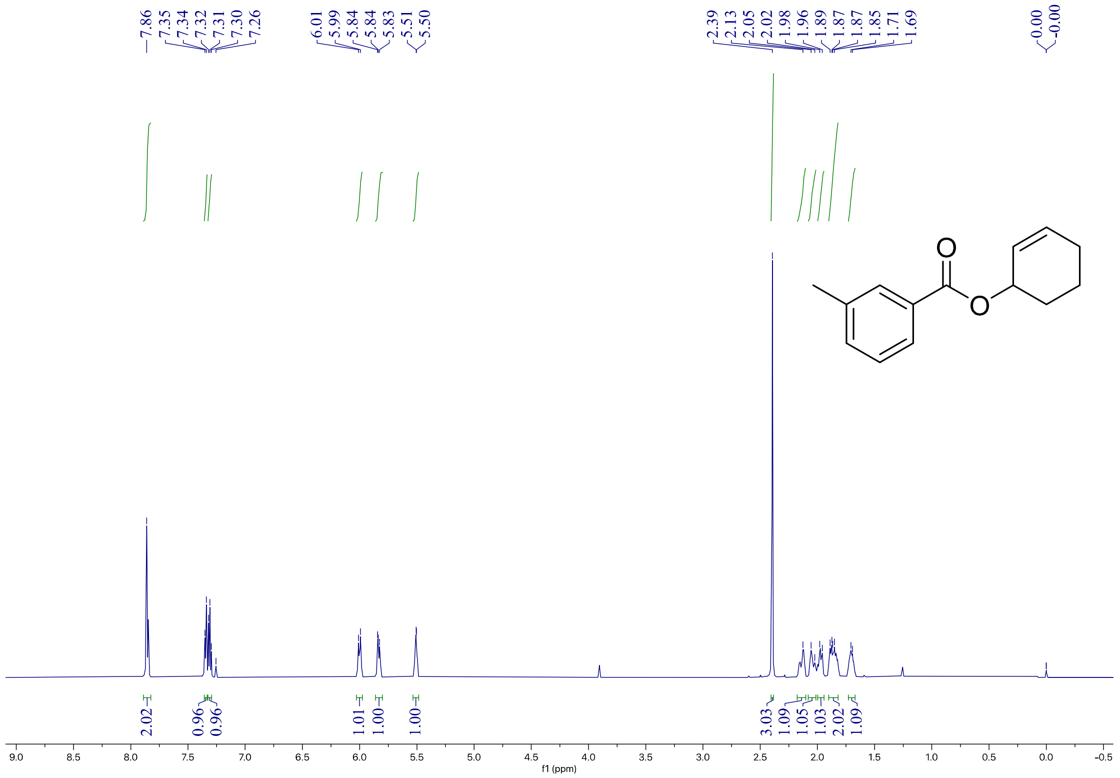

^{13}C NMR of cyclohex-2-en-1-yl 3-methylbenzoate (**3a**)

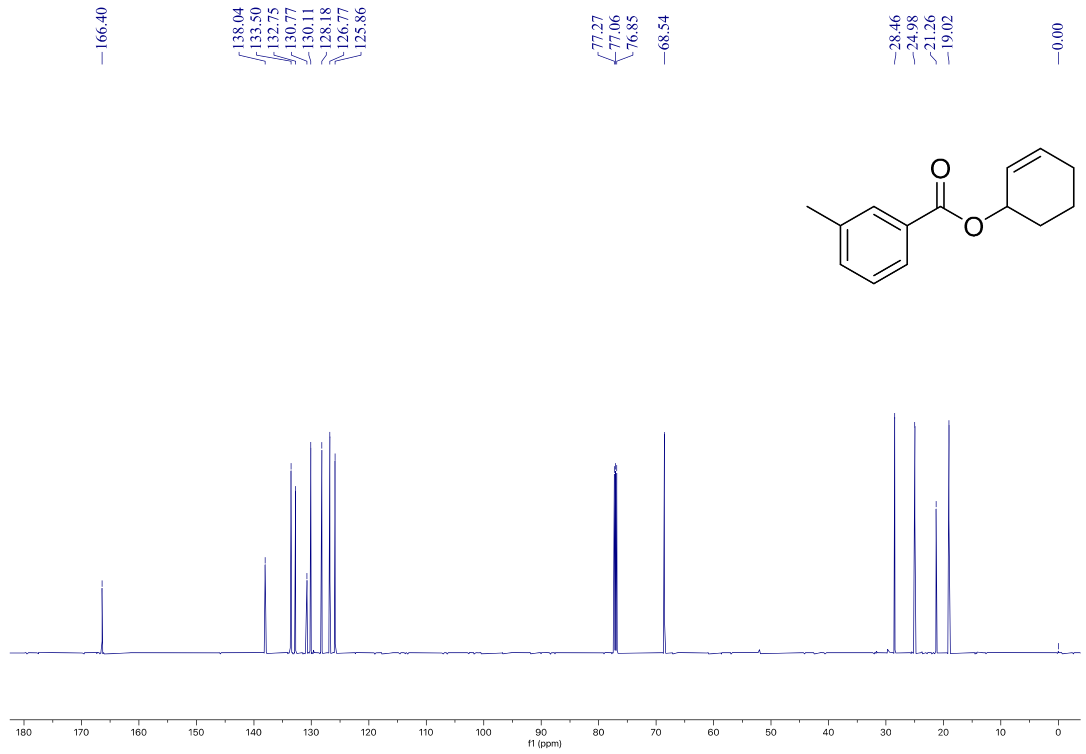

^{1}H NMR of cyclohex-2-en-1-yl 4-methylbenzoate (**4a**)

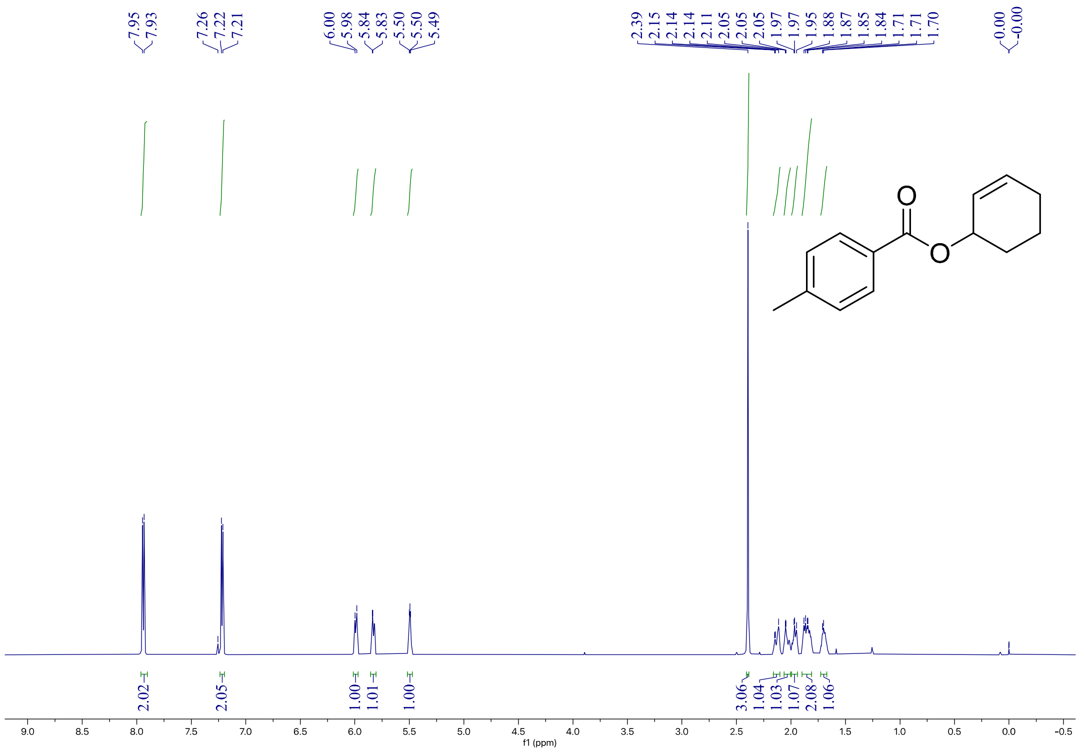

^{13}C NMR of cyclohex-2-en-1-yl 4-methylbenzoate (**4a**)

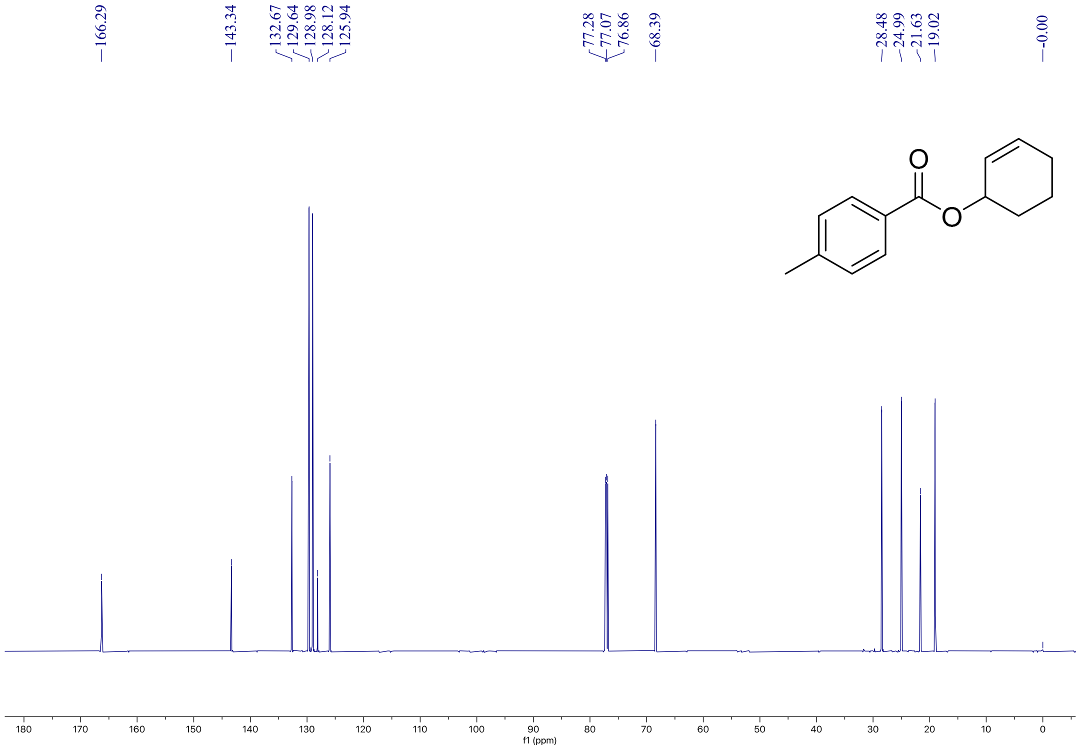

^{1}H NMR of cyclohex-2-en-1-yl 2-methoxybenzoate (**5a**)

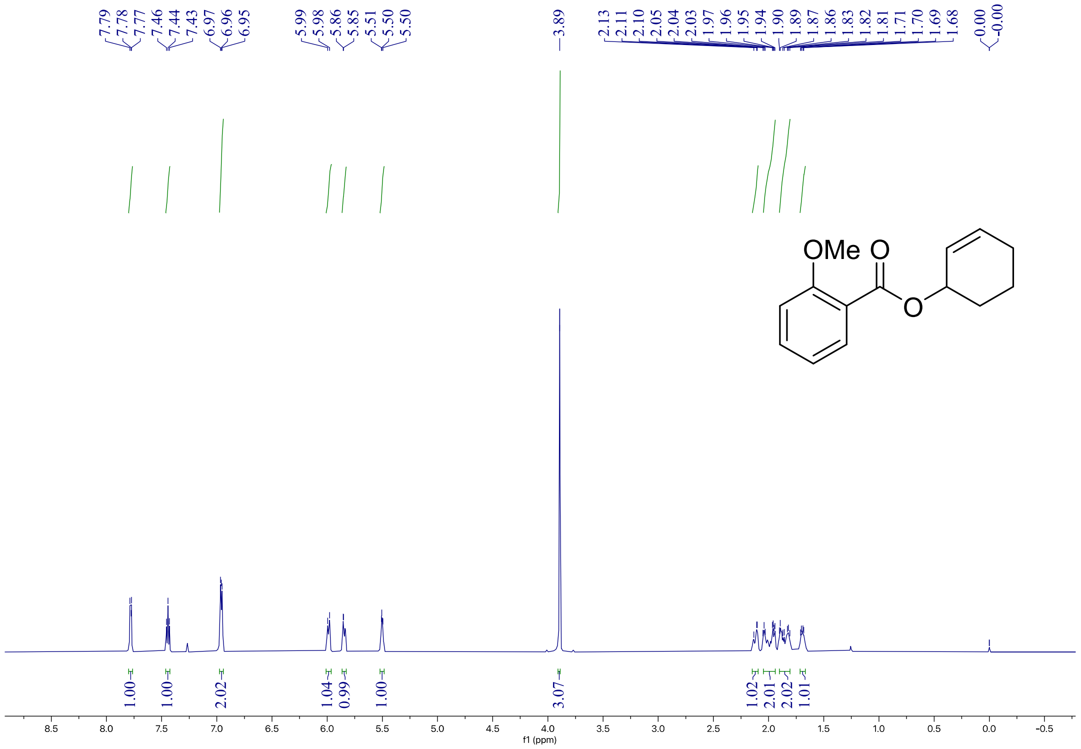

^{13}C NMR of cyclohex-2-en-1-yl 2-methoxybenzoate (**5a**)

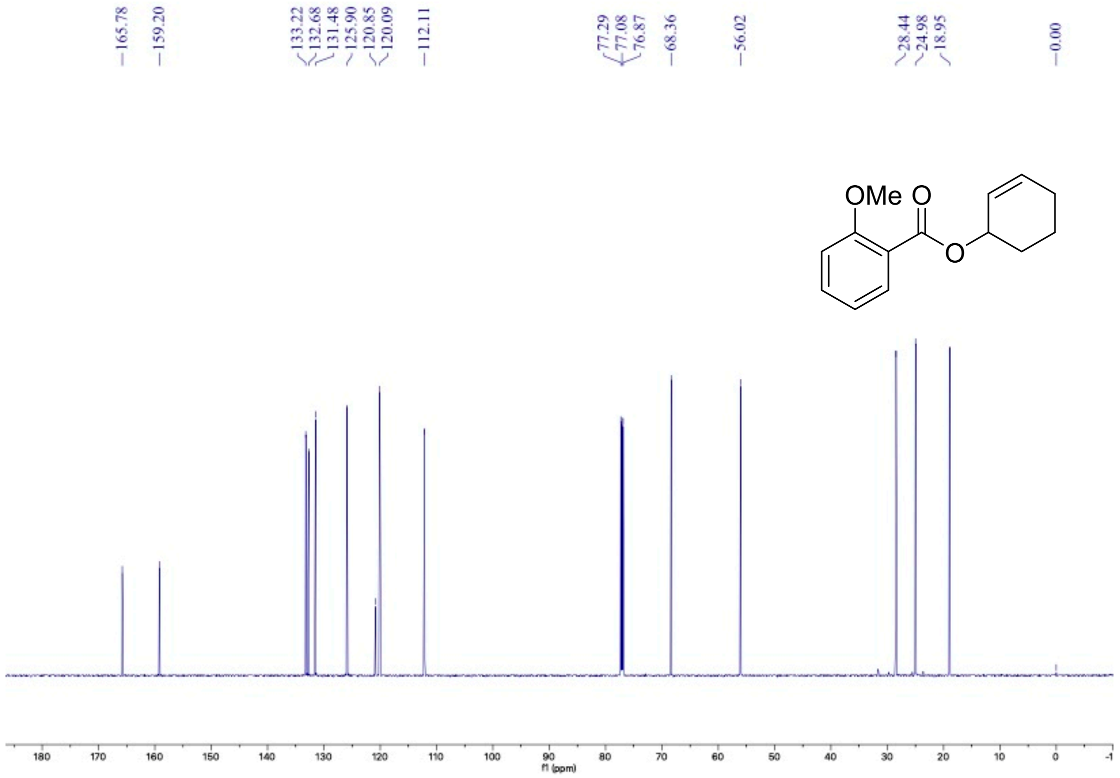

^{1}H NMR of cyclohex-2-en-1-yl 3-methoxybenzoate (**6a**)

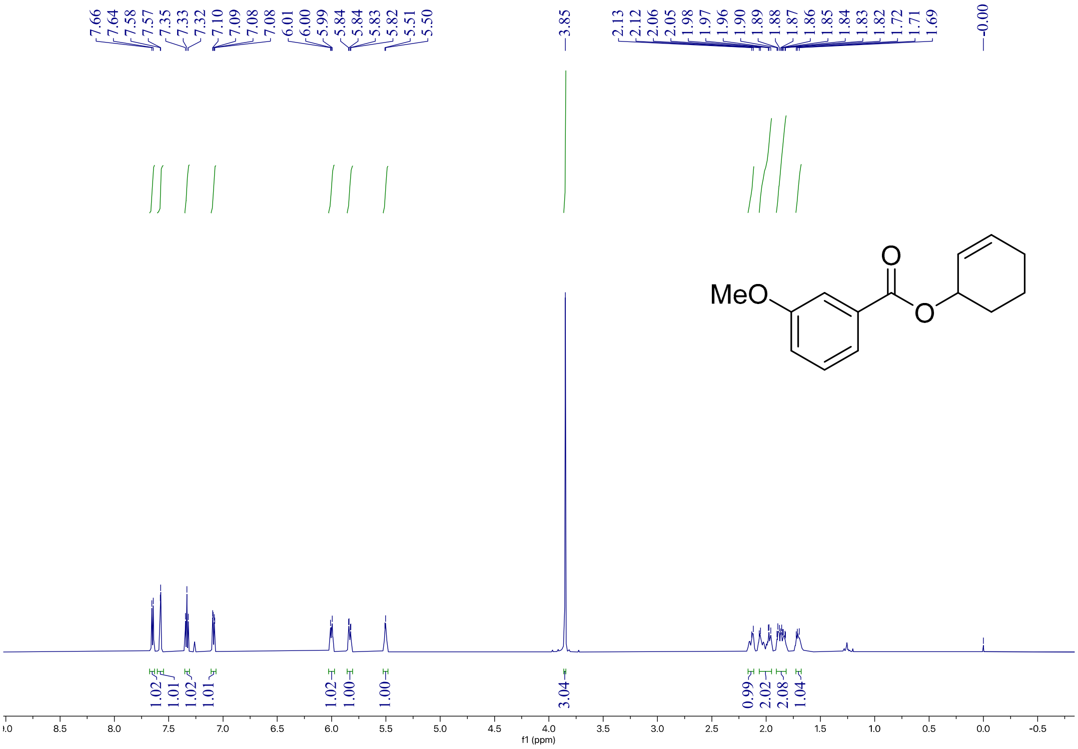

^{13}C NMR of cyclohex-2-en-1-yl 3-methoxybenzoate (**6a**)

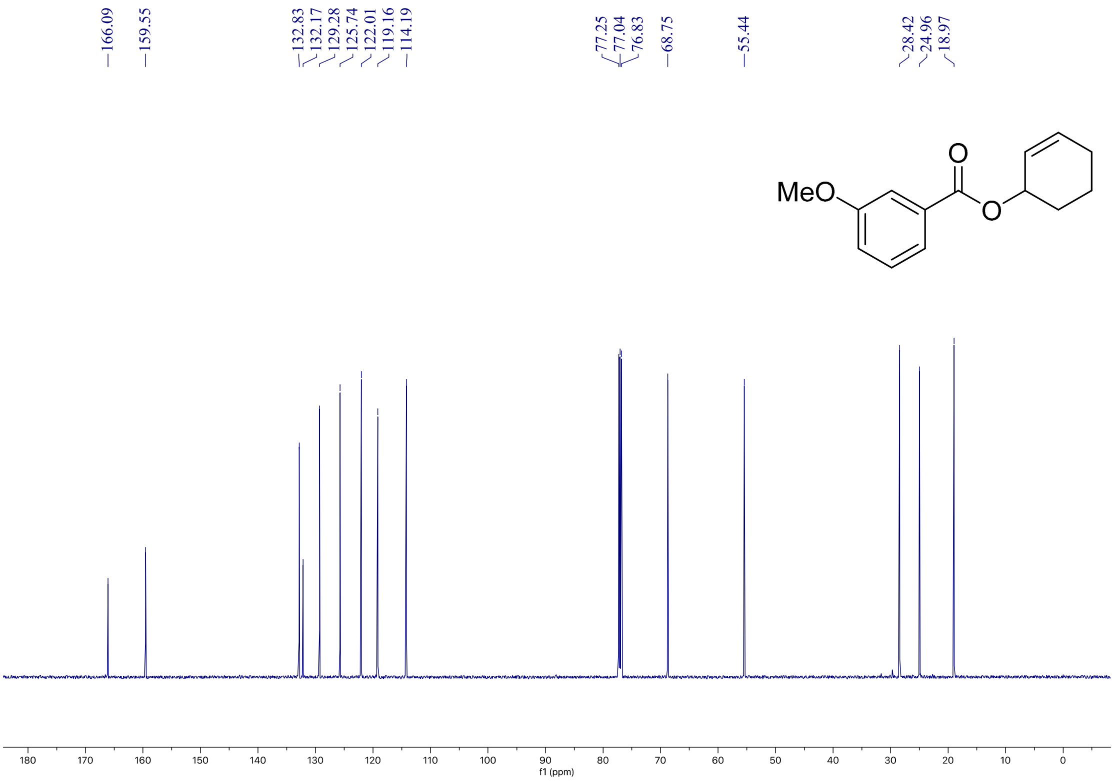

^1H NMR of cyclohex-2-en-1-yl 4-methoxybenzoate (**7a**)

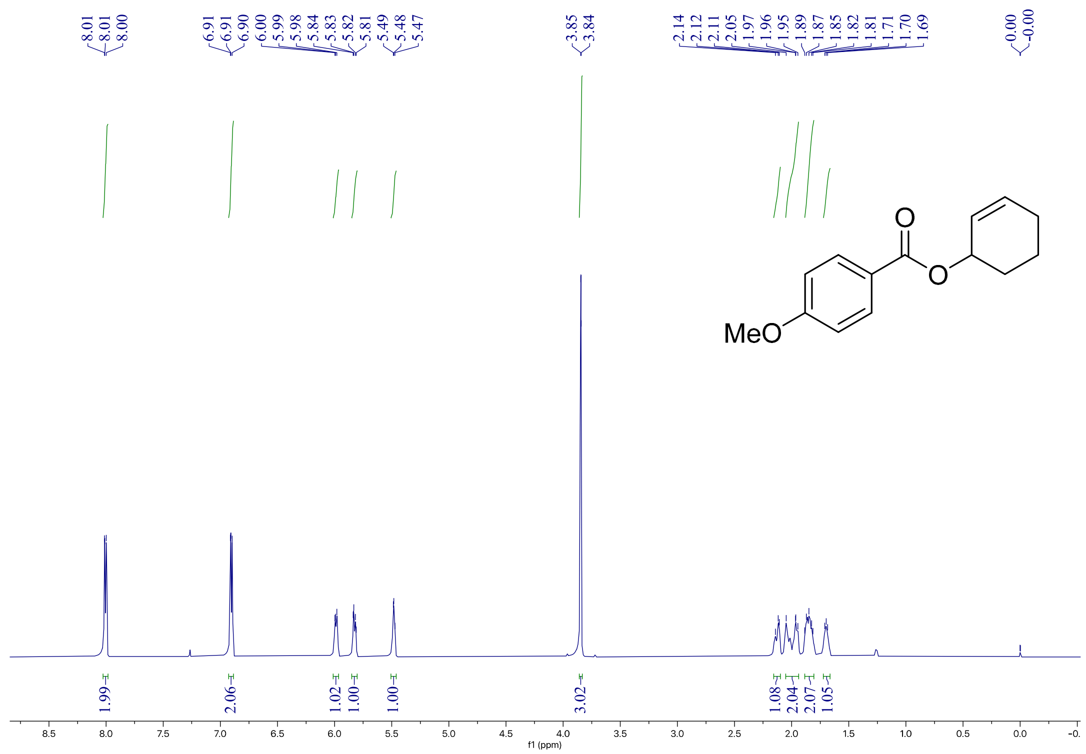

^{13}C NMR of cyclohex-2-en-1-yl 4-methoxybenzoate (**7a**)

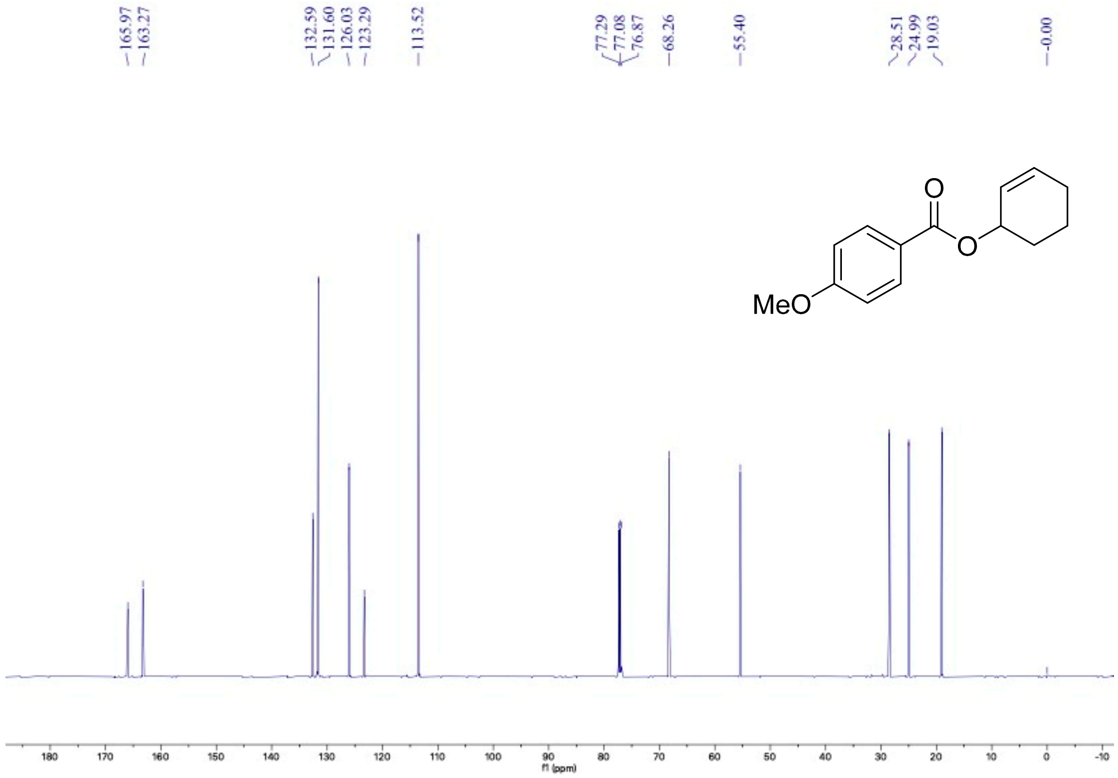

^{1}H NMR of cyclohex-2-en-1-yl [1,1'-biphenyl]-4-carboxylate (**8a**)

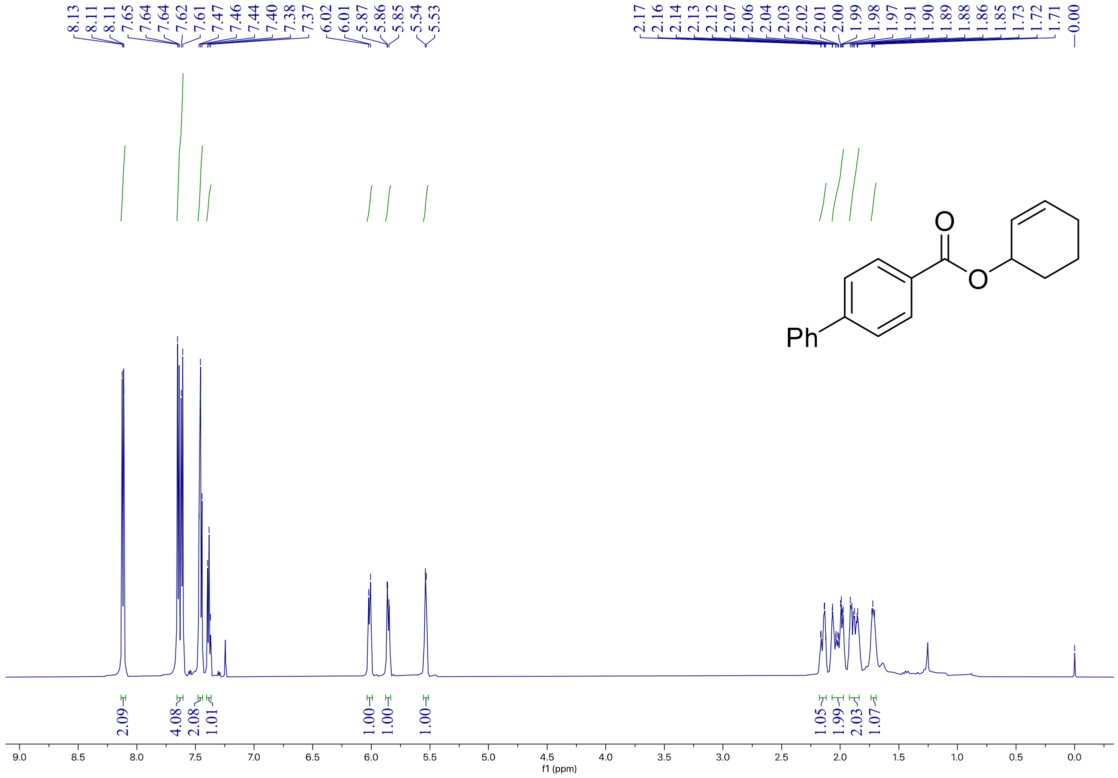

^{13}C NMR of cyclohex-2-en-1-yl [1,1'-biphenyl]-4-carboxylate (**8a**)

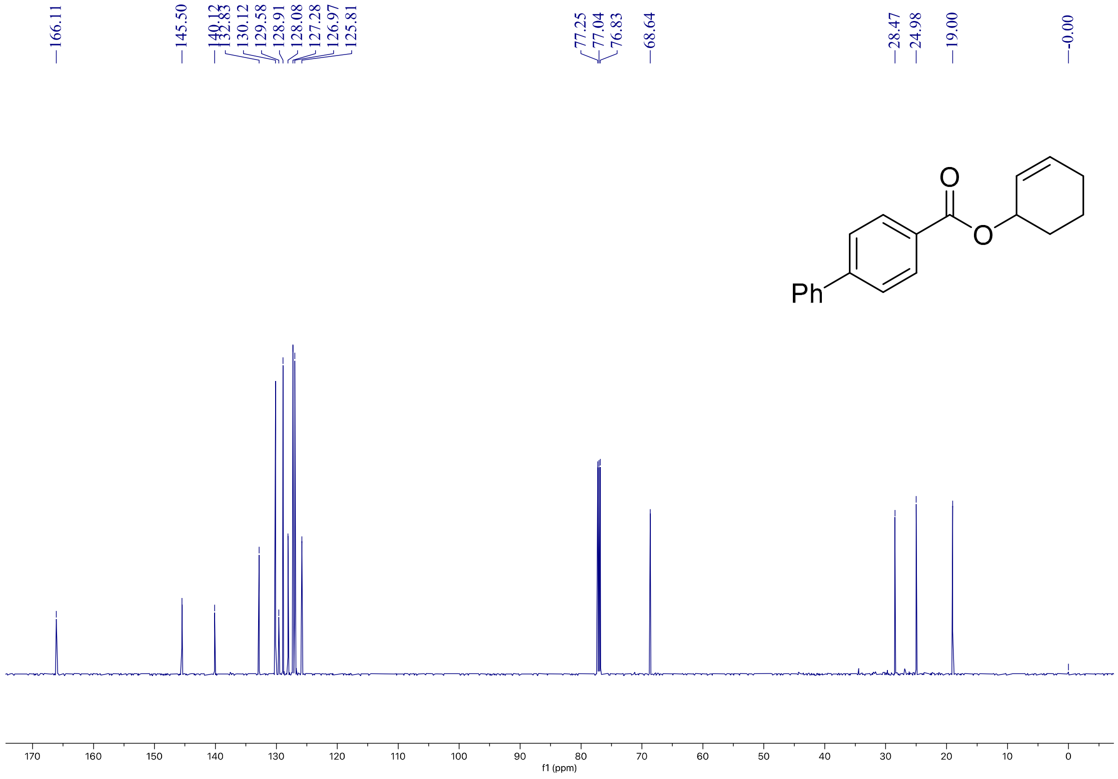

^{1}H NMR of cyclohex-2-en-1-yl 4-phenoxybenzoate (**9a**)

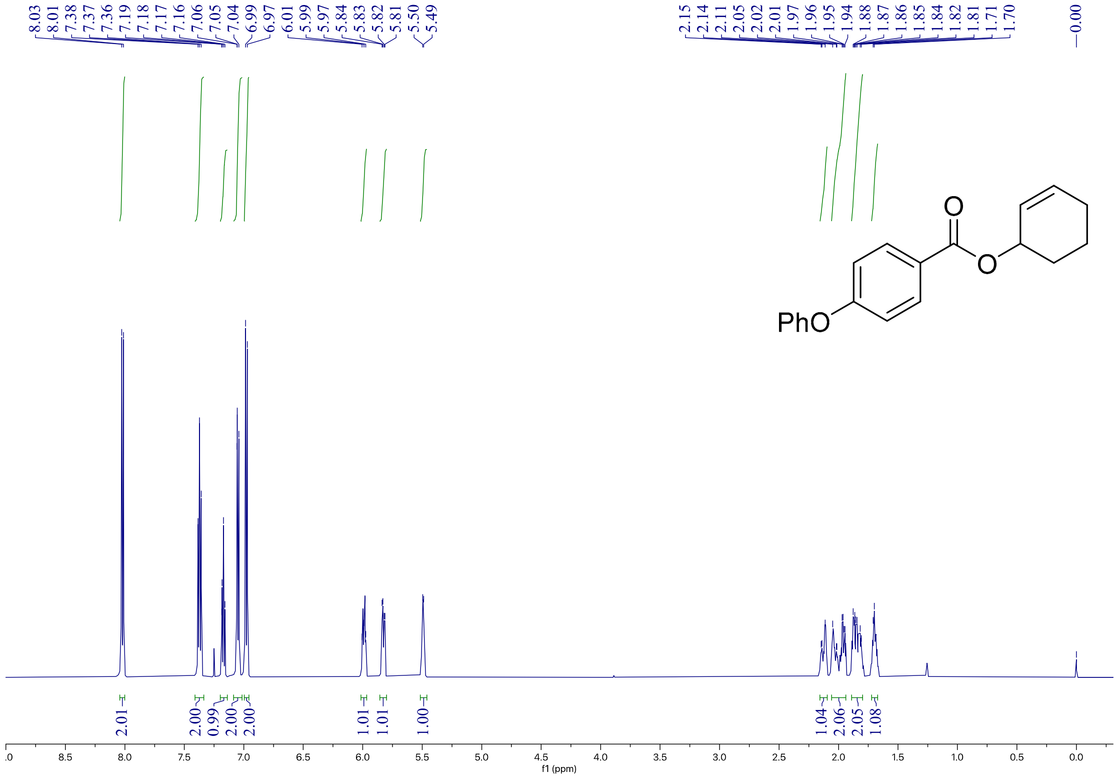

^{13}C NMR of cyclohex-2-en-1-yl 4-phenoxybenzoate (**9a**)

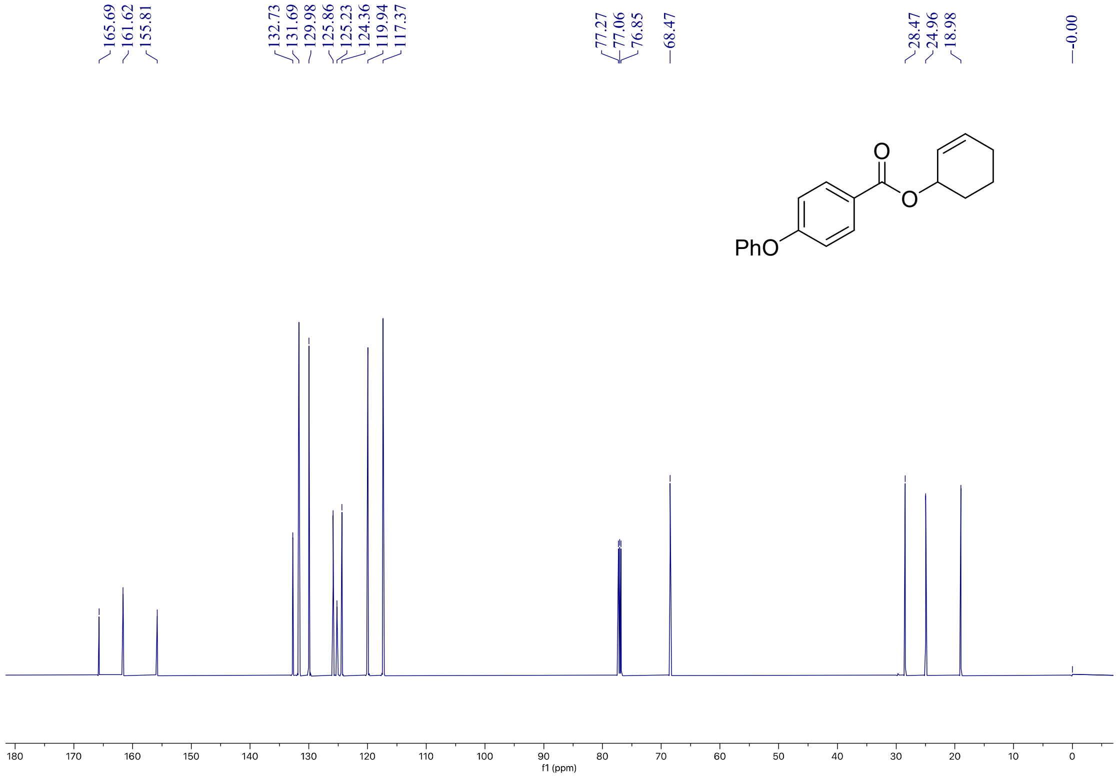

^{1}H NMR of cyclohex-2-en-1-yl 4-(*tert*-butyl)benzoate (**10a**)

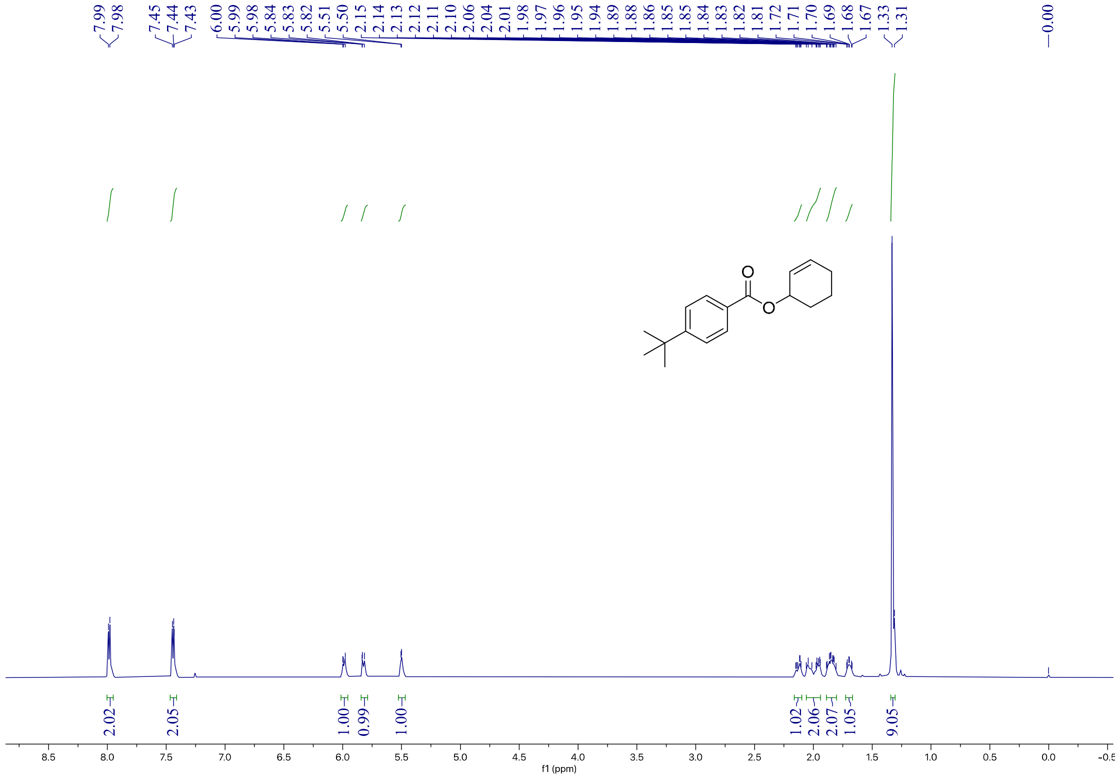

^{13}C NMR of cyclohex-2-en-1-yl 4-(*tert*-butyl)benzoate (**10a**)

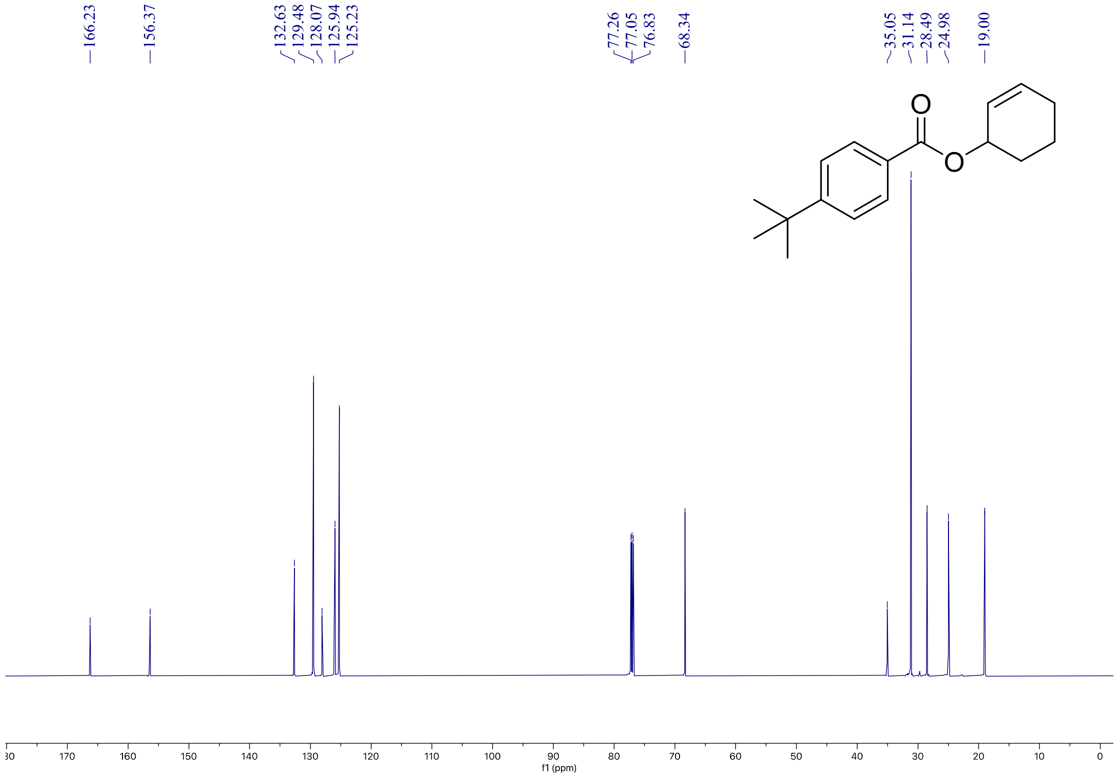

^{1}H NMR of cyclohex-2-en-1-yl 3,5-dimethylbenzoate (**11a**)

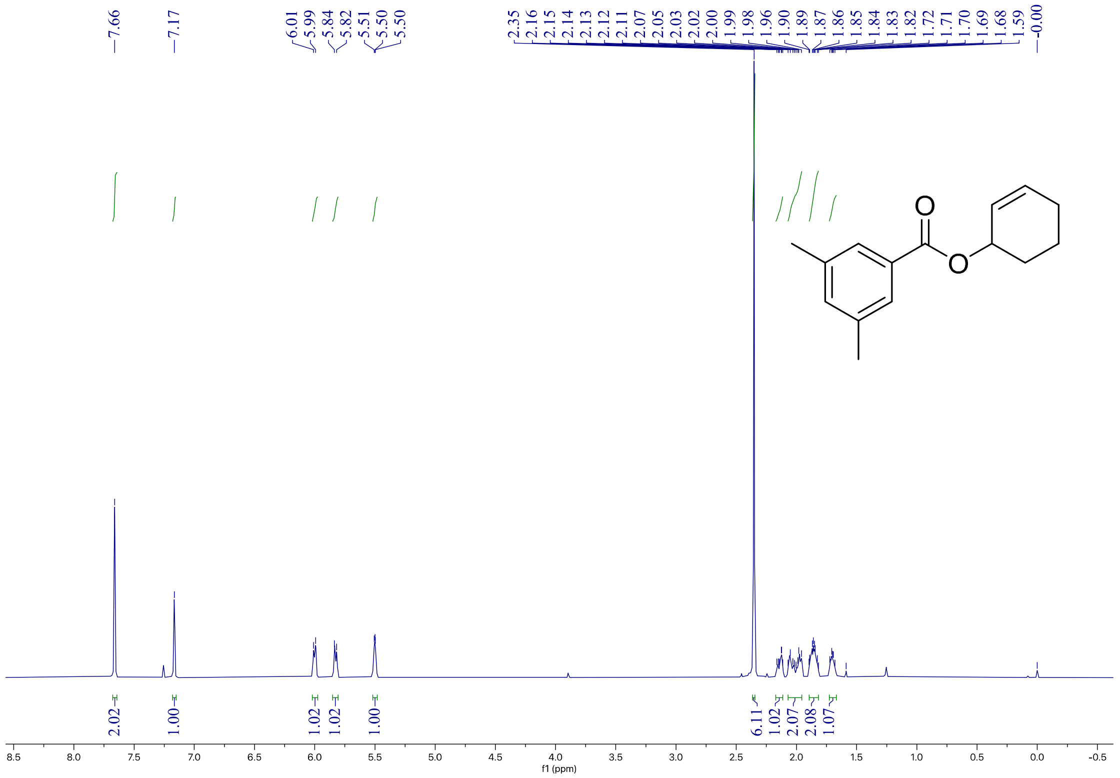

^{13}C NMR of cyclohex-2-en-1-yl 3,5-dimethylbenzoate (**11a**)

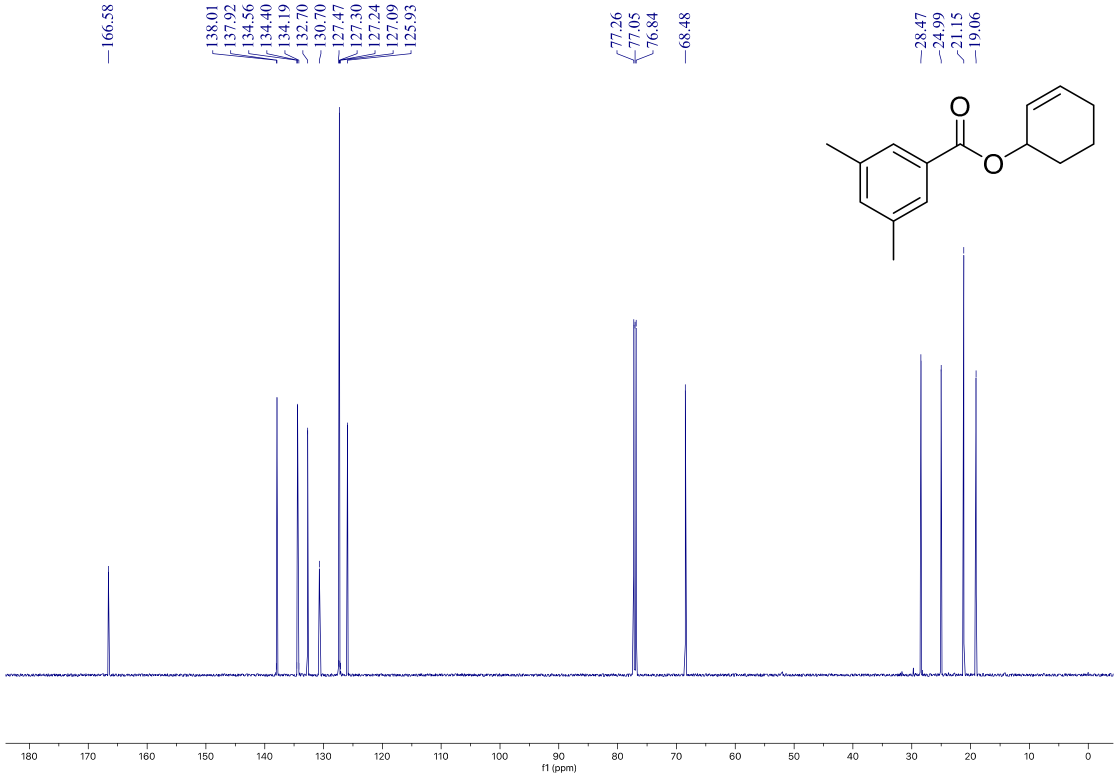

^{1}H NMR of cyclohex-2-en-1-yl 3,4-dimethylbenzoate (**12a**)

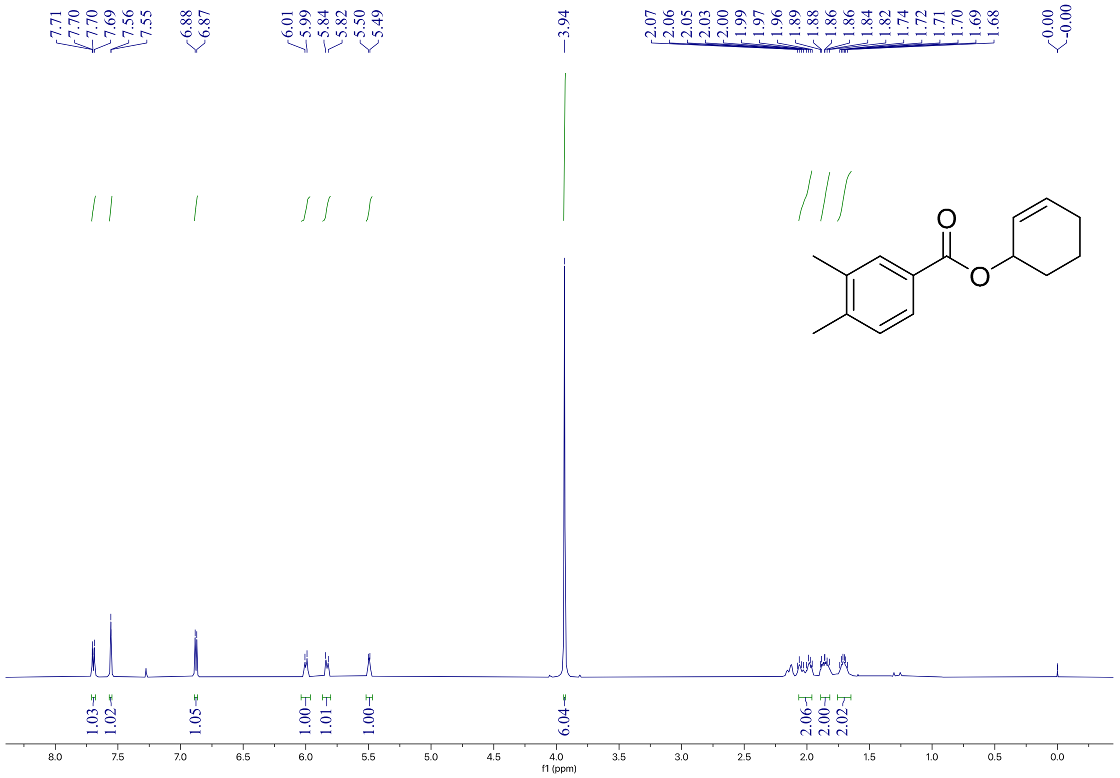

^{13}C NMR of cyclohex-2-en-1-yl 3,4-dimethylbenzoate (**12a**)

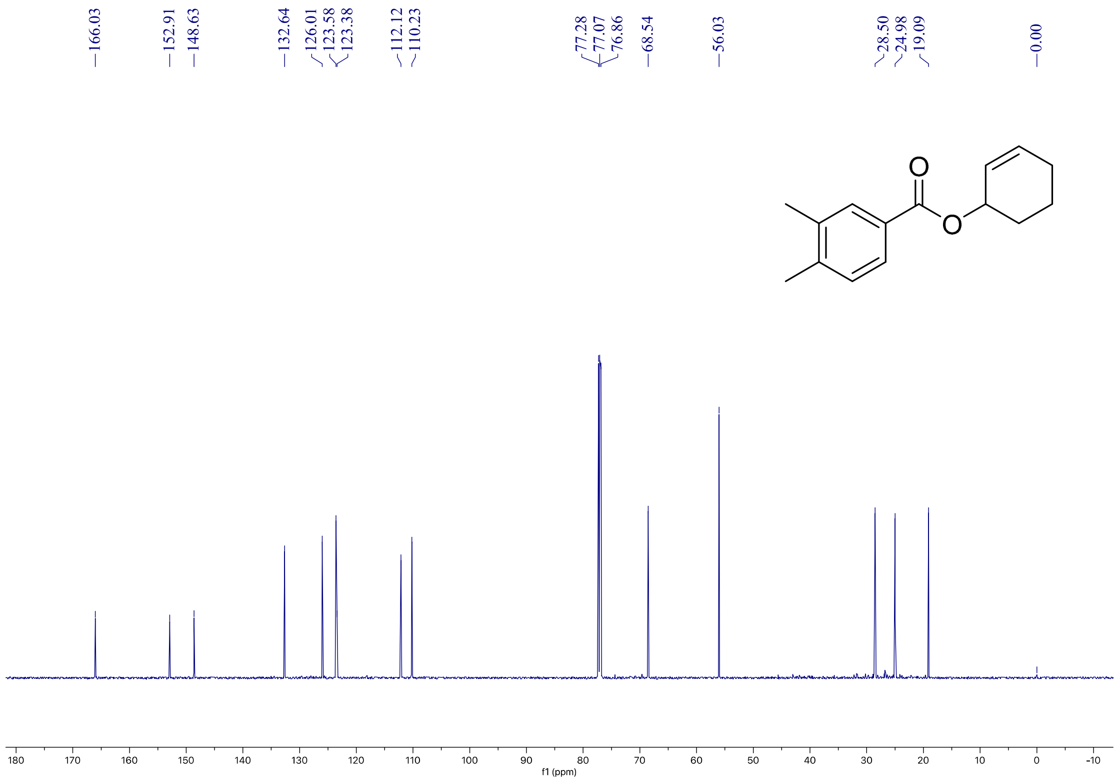

^{1}H NMR of cyclohex-2-en-1-yl 2,4,6-trimethylbenzoate (**13a**)

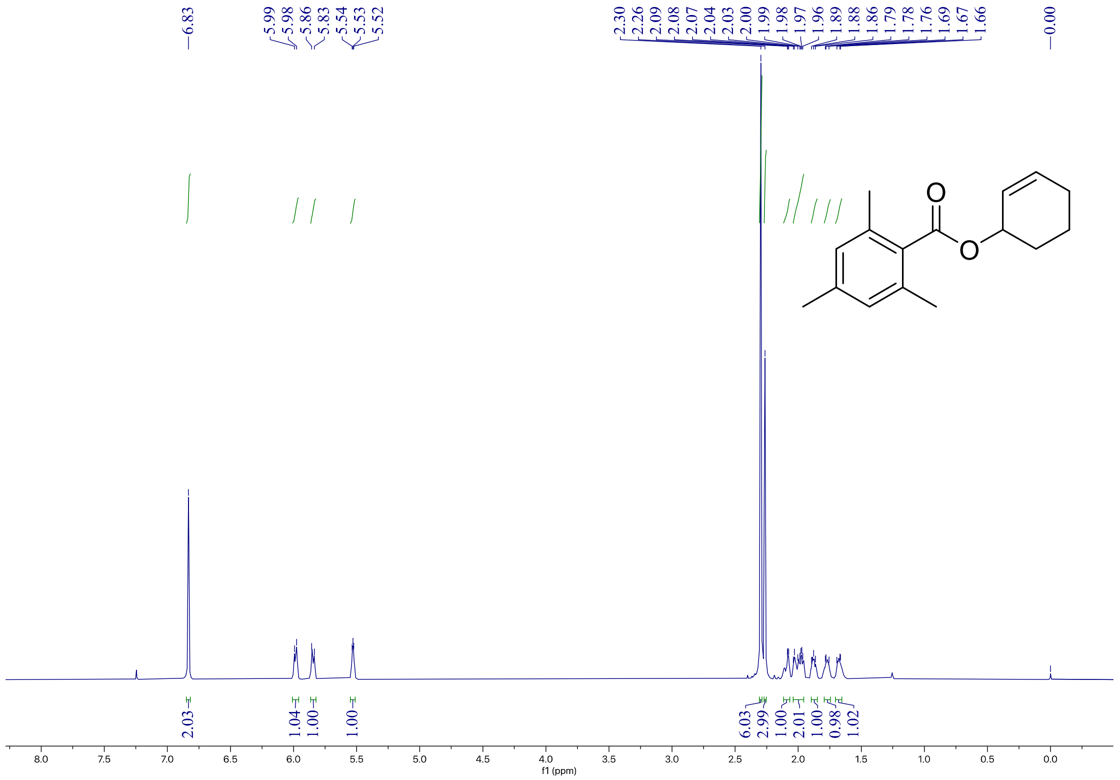

^{13}C NMR of cyclohex-2-en-1-yl 2,4,6-trimethylbenzoate (**13a**)

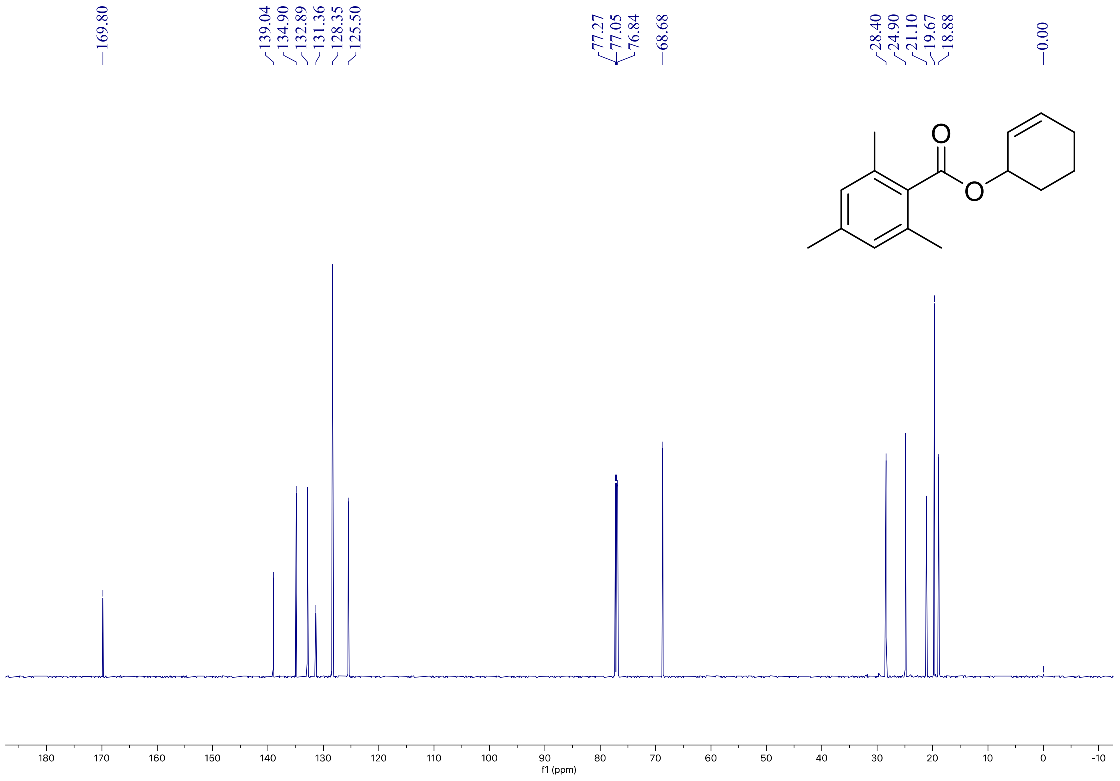

^{1}H NMR of cyclohex-2-en-1-yl 2-fluorobenzoate (**14a**)

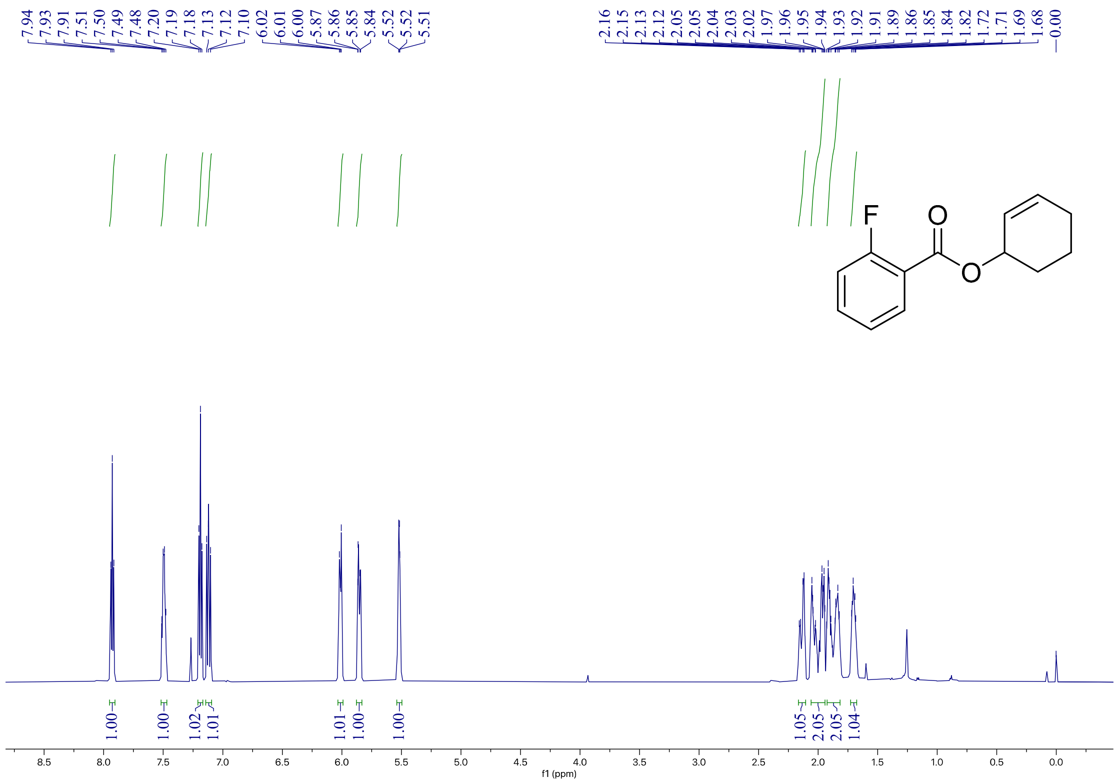

^{13}C NMR of cyclohex-2-en-1-yl 2-fluorobenzoate (**14a**)

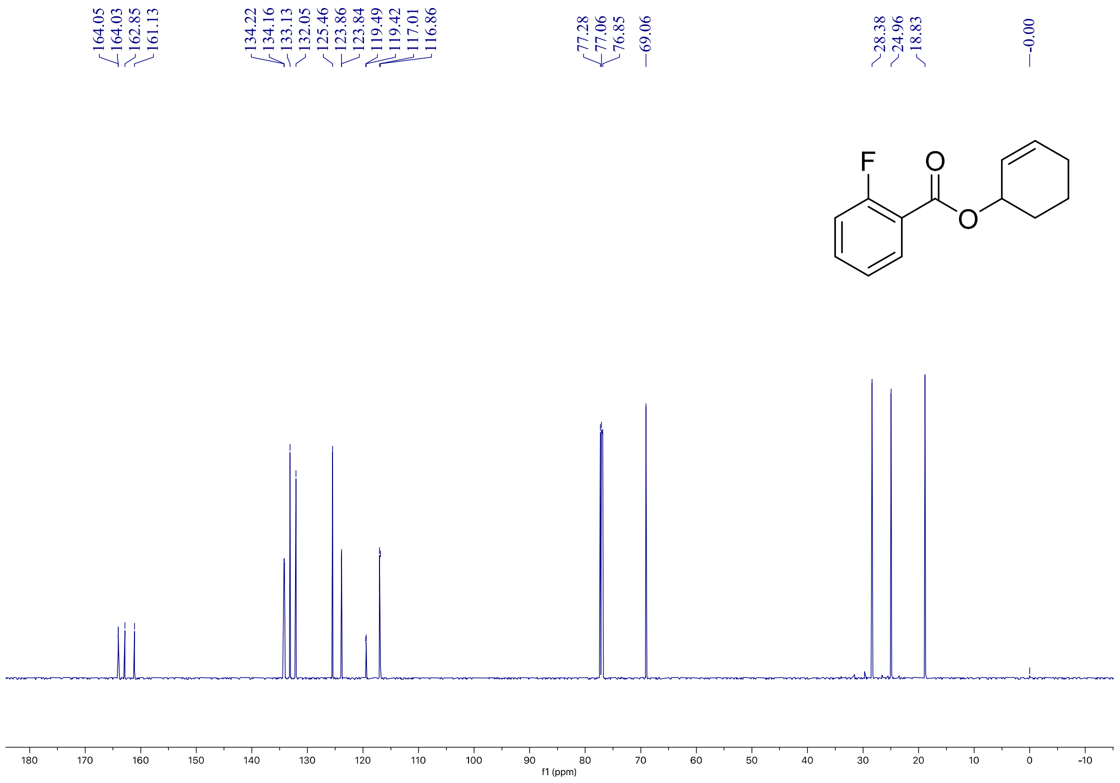

^{1}H NMR of cyclohex-2-en-1-yl 4-fluorobenzoate (**15a**)

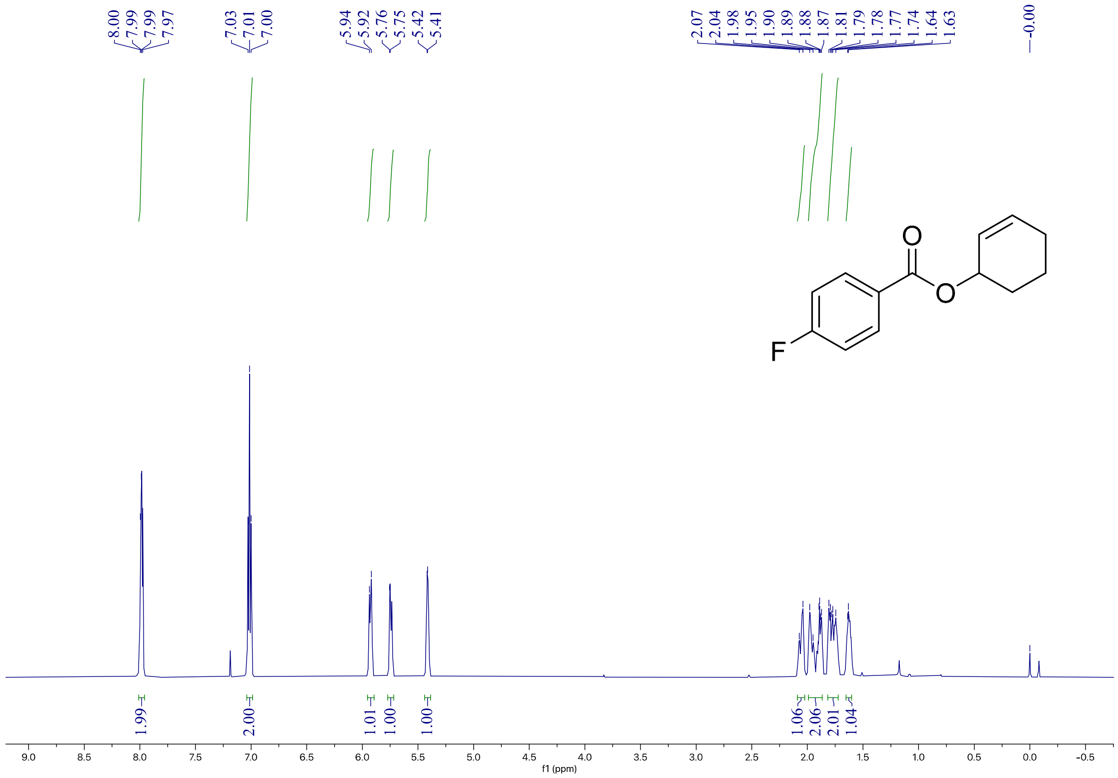

^{13}C NMR of cyclohex-2-en-1-yl 4-fluorobenzoate (**15a**)

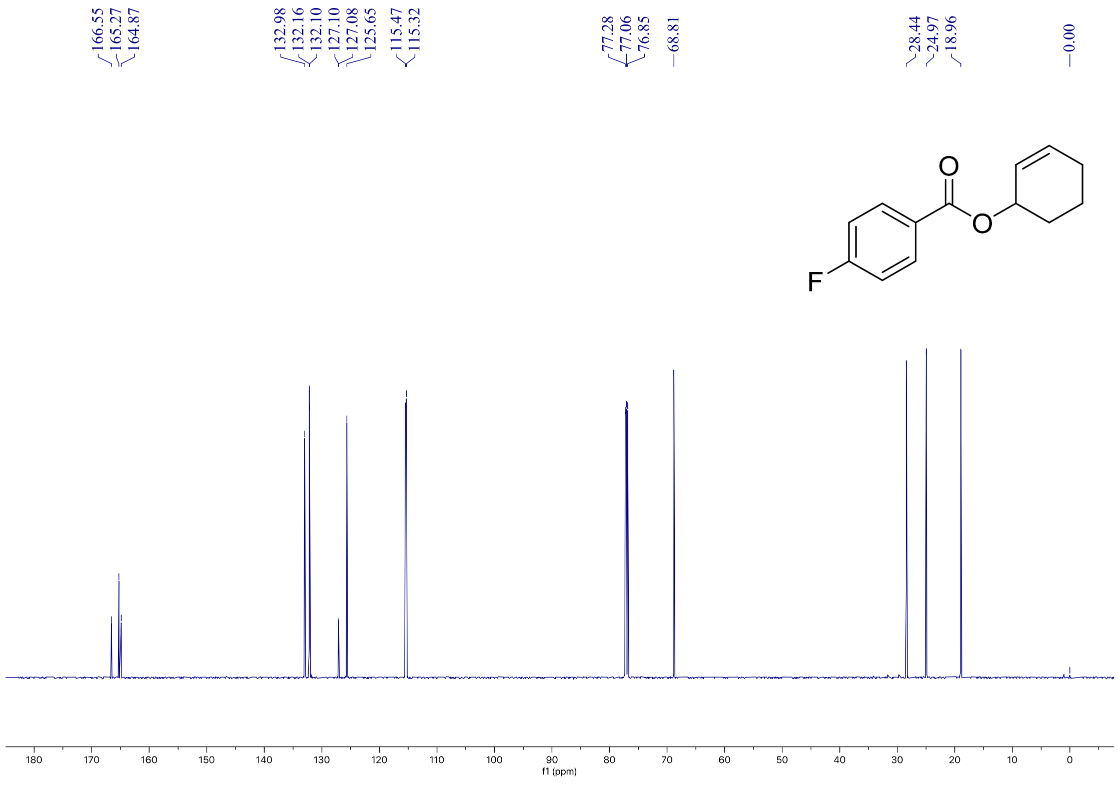

^{1}H NMR of cyclohex-2-en-1-yl 2-chlorobenzoate (**16a**)

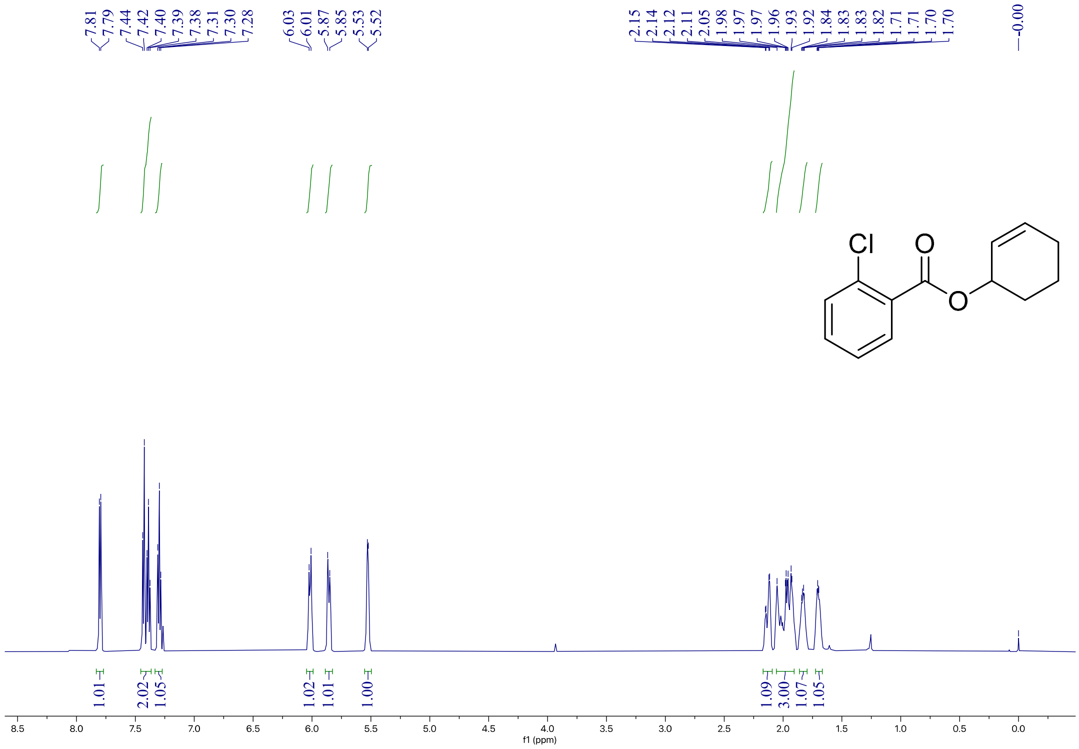

^{13}C NMR of cyclohex-2-en-1-yl 2-chlorobenzoate (**16a**)

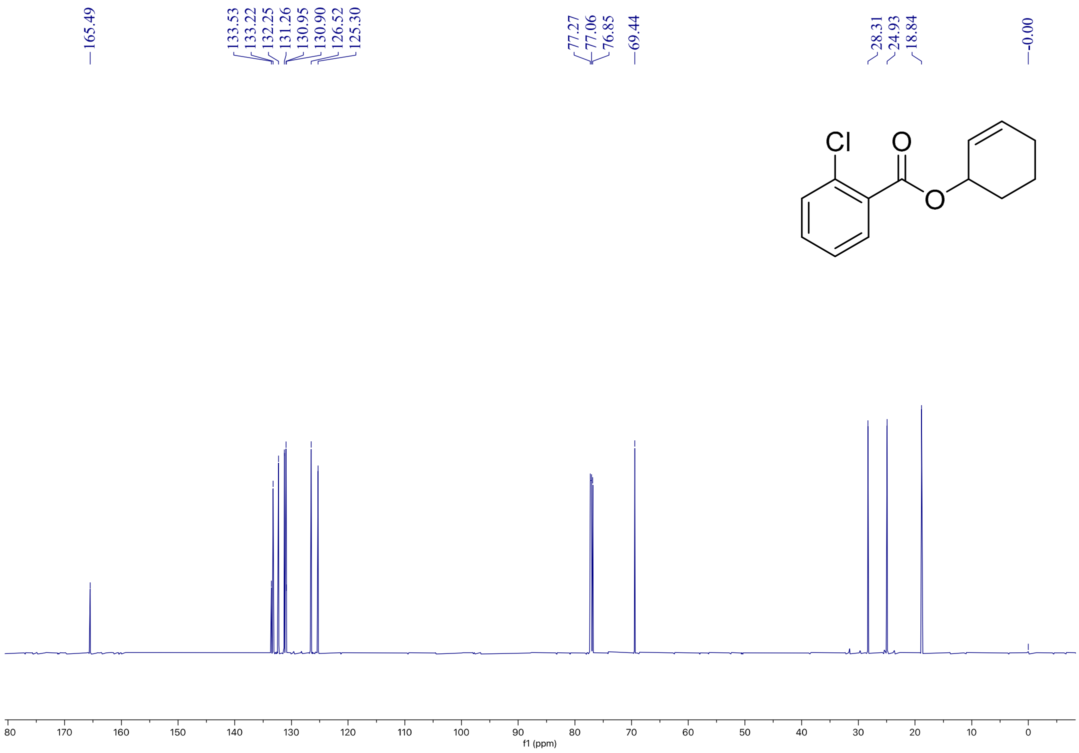

^{1}H NMR of cyclohex-2-en-1-yl 3-chlorobenzoate (**17a**)

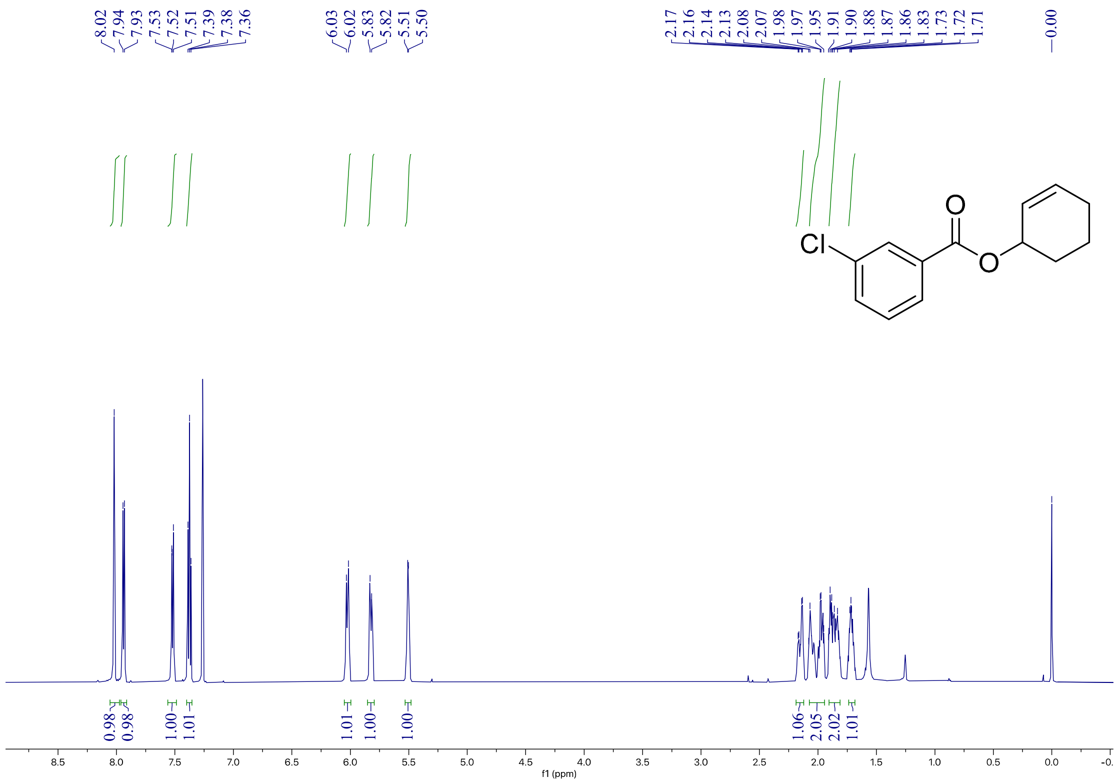

^{13}C NMR of cyclohex-2-en-1-yl 3-chlorobenzoate (**17a**)

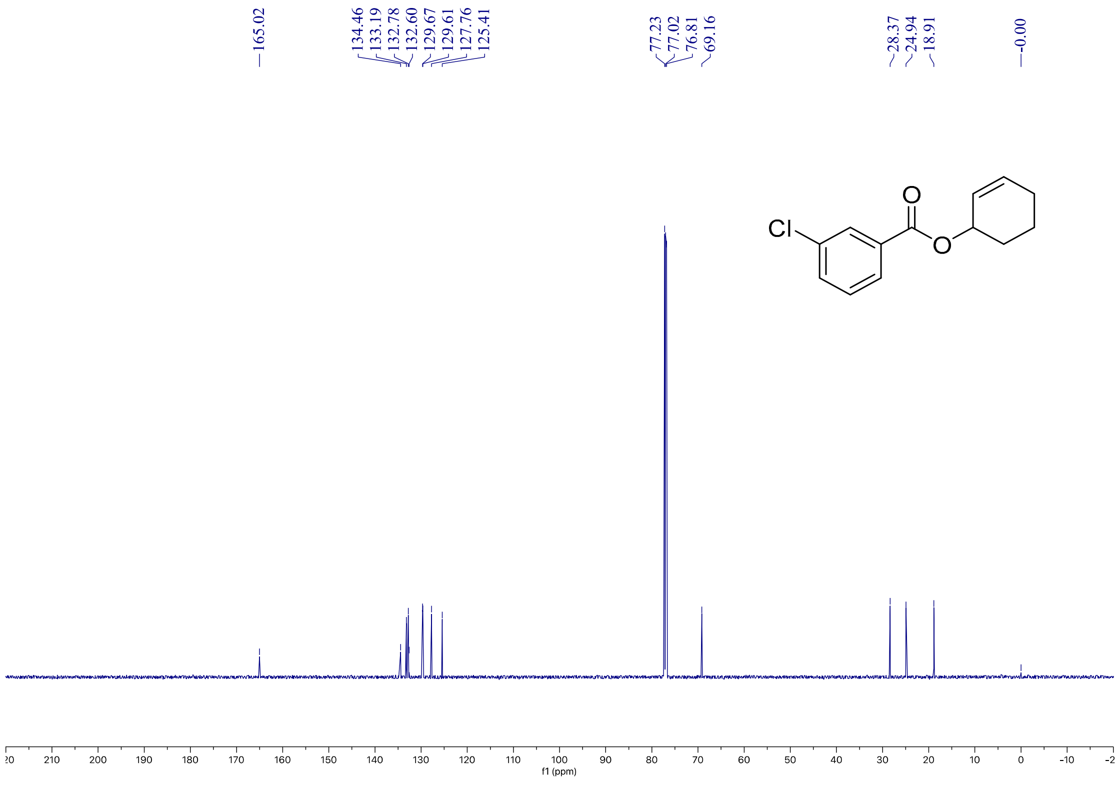

^{1}H NMR of cyclohex-2-en-1-yl 4-chlorobenzoate (**18a**)

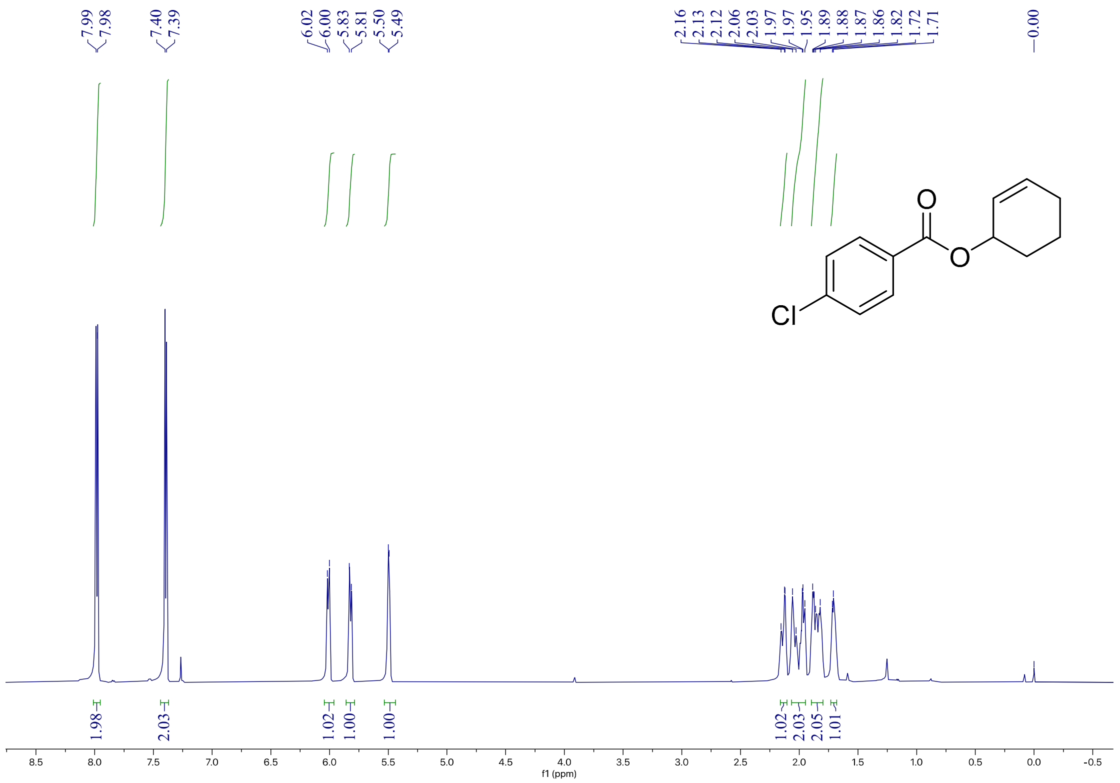

^{13}C NMR of cyclohex-2-en-1-yl 4-chlorobenzoate (**18a**)

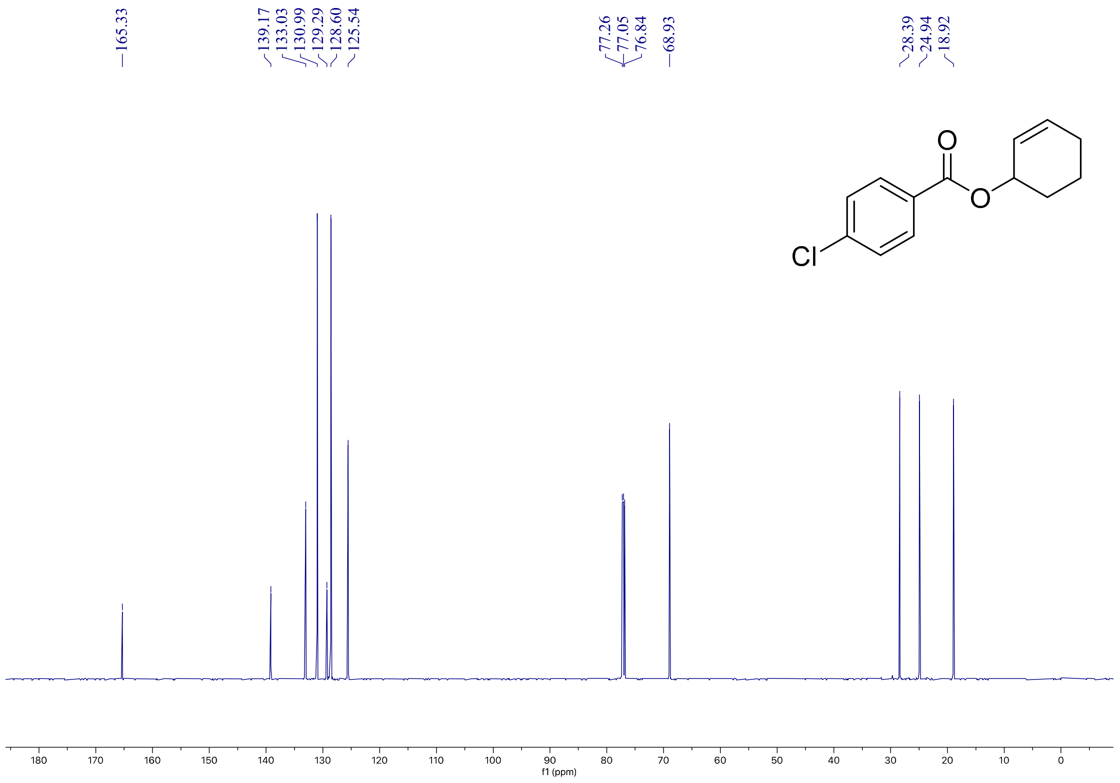

^{1}H NMR of cyclohex-2-en-1-yl 2-bromobenzoate (**19a**)

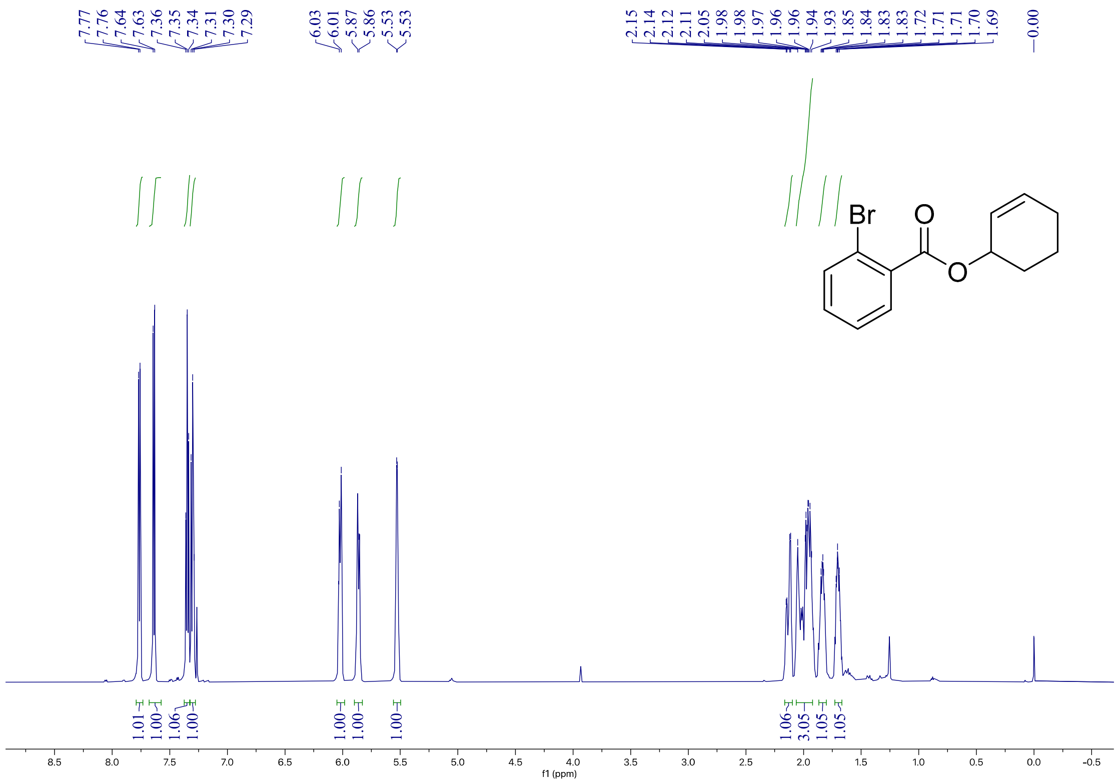

^{13}C NMR of cyclohex-2-en-1-yl 2-bromobenzoate (**19a**)

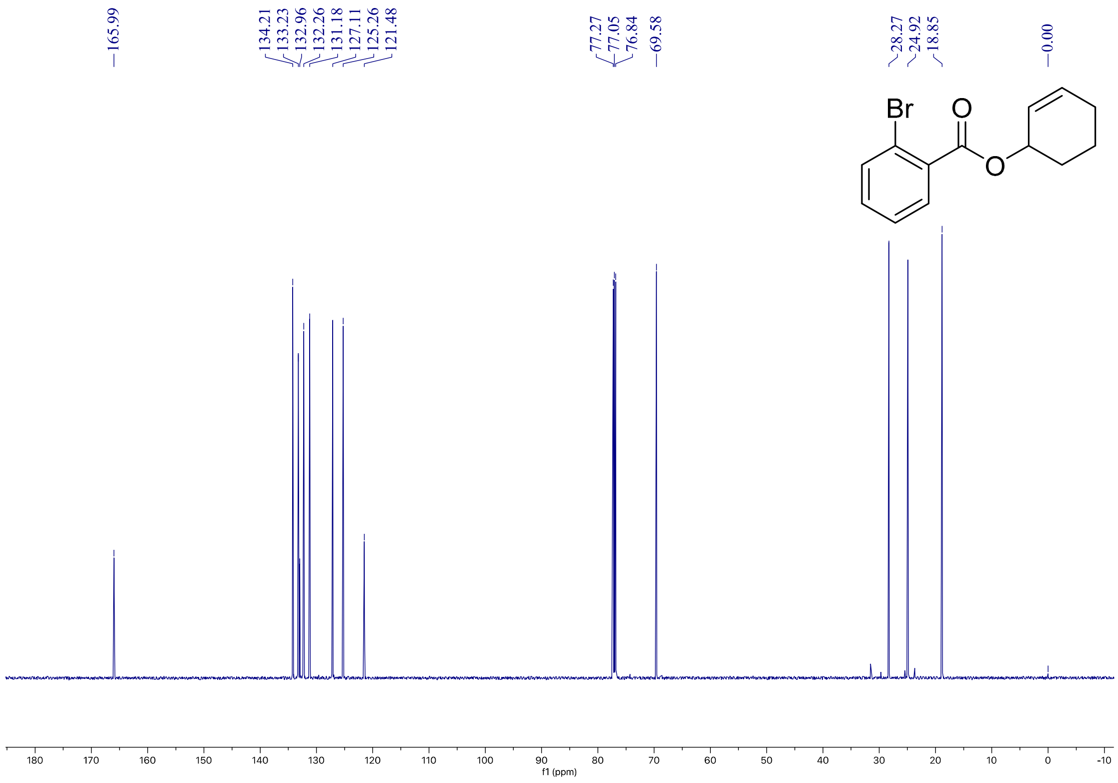

^{1}H NMR of cyclohex-2-en-1-yl 4-bromobenzoate (**20a**):

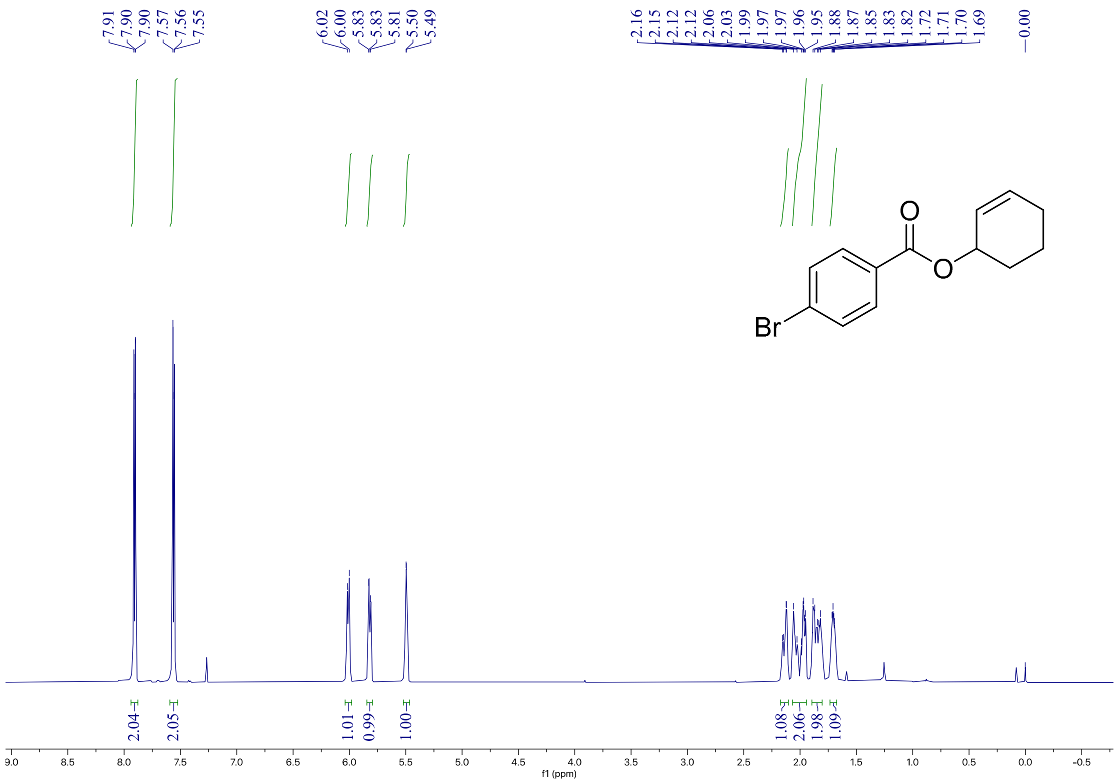

^{13}C NMR of cyclohex-2-en-1-yl 4-bromobenzoate (**20a**)

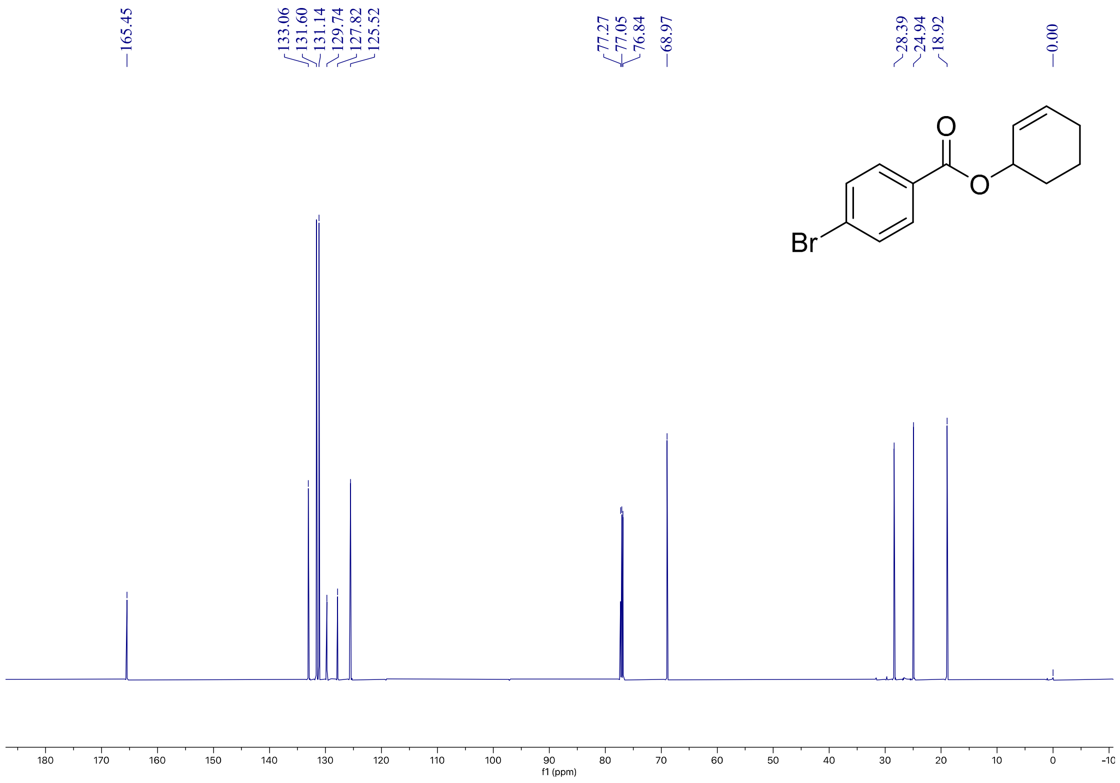

^{1}H NMR of cyclohex-2-en-1-yl 2-iodobenzoate (**21a**)

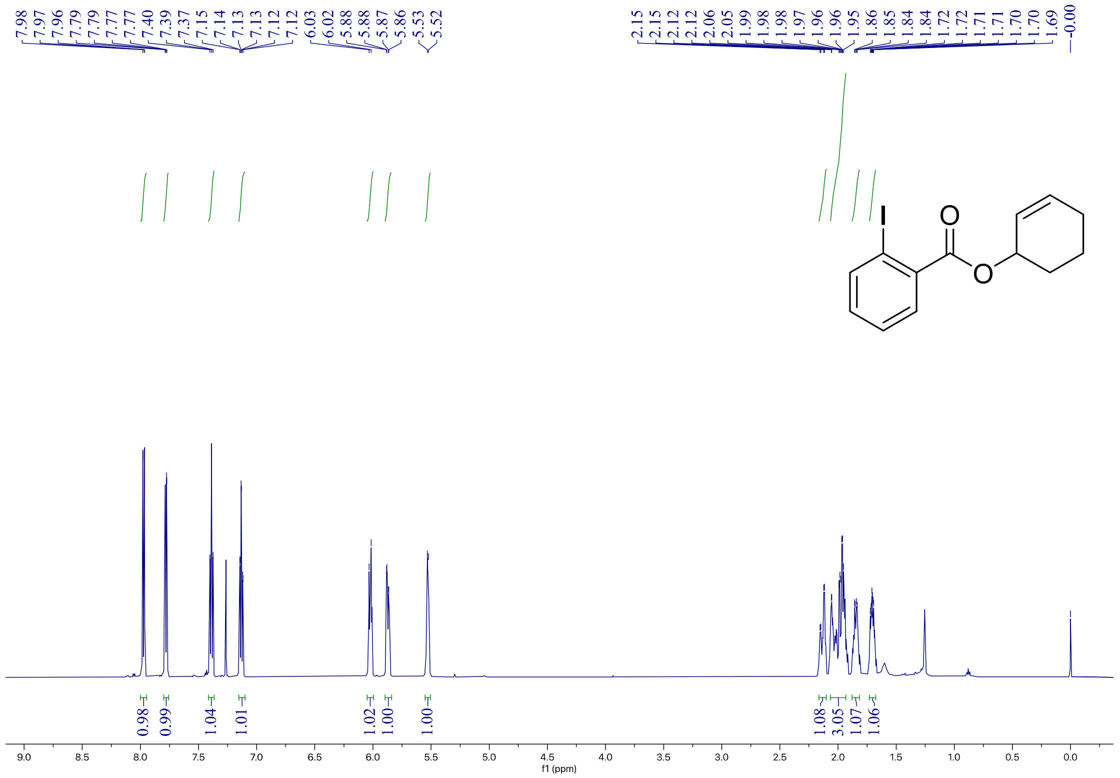

^{13}C NMR of cyclohex-2-en-1-yl 2-iodobenzoate (**21a**)

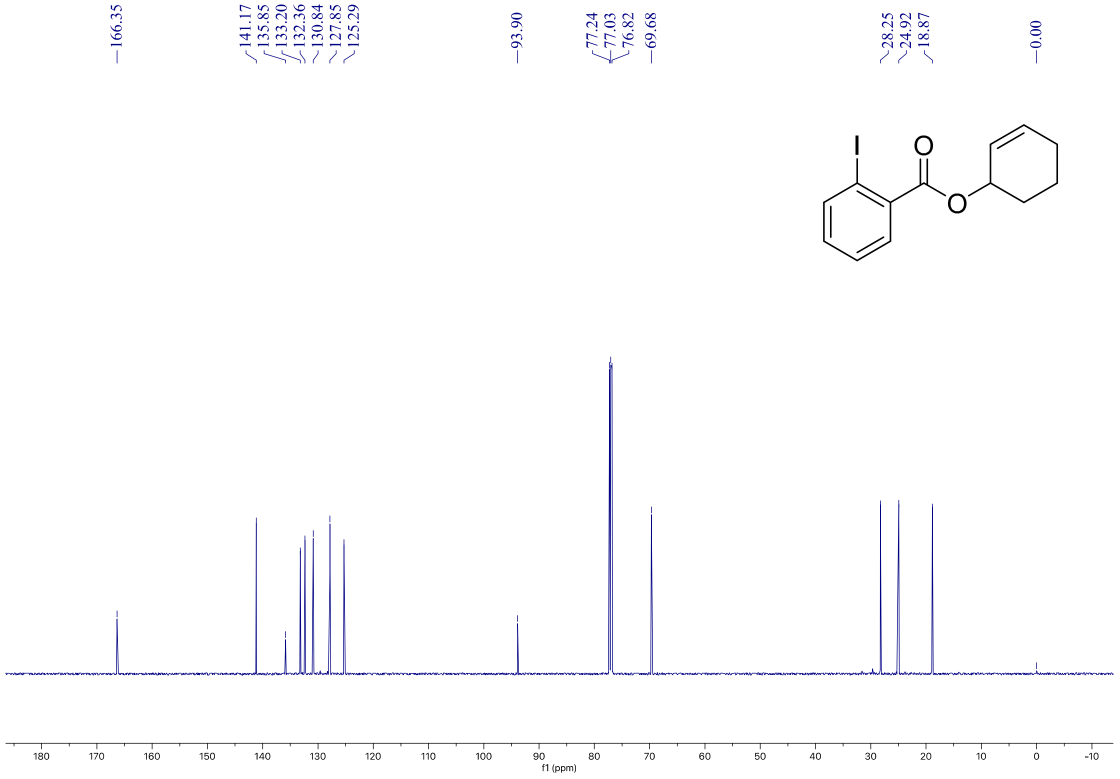

^{1}H NMR of cyclohex-2-en-1-yl 4-iodobenzoate (**22a**)

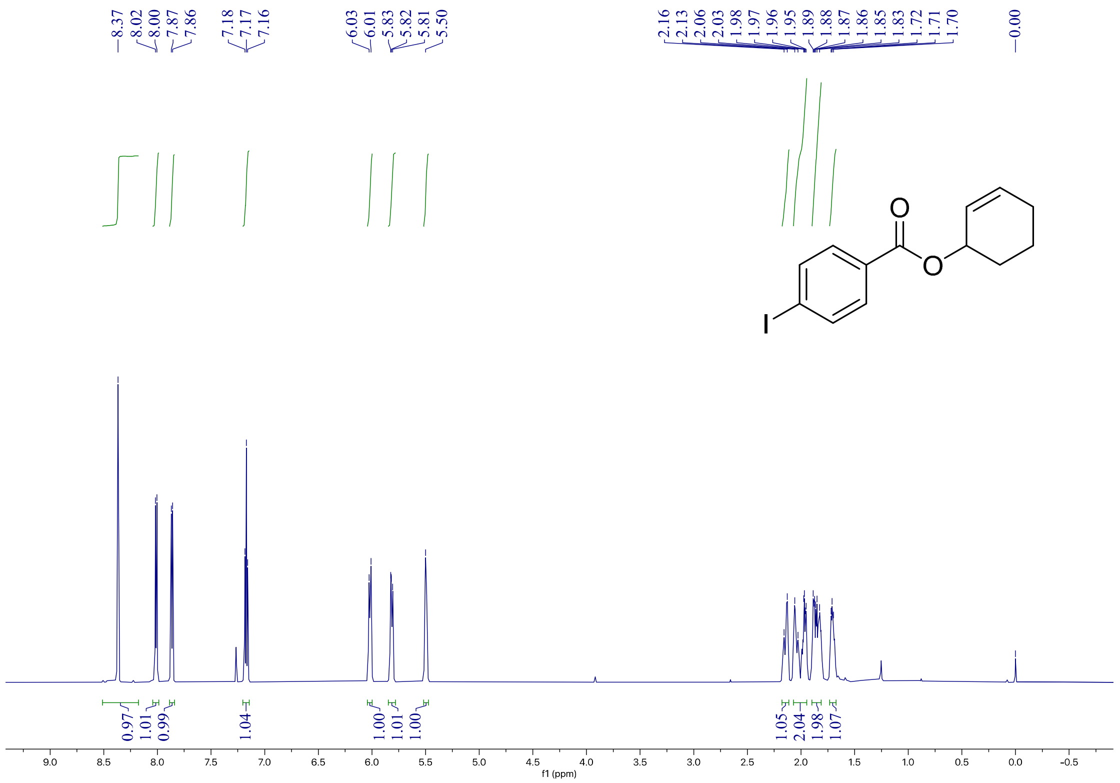

^{13}C NMR of cyclohex-2-en-1-yl 4-iodobenzoate (**22a**)

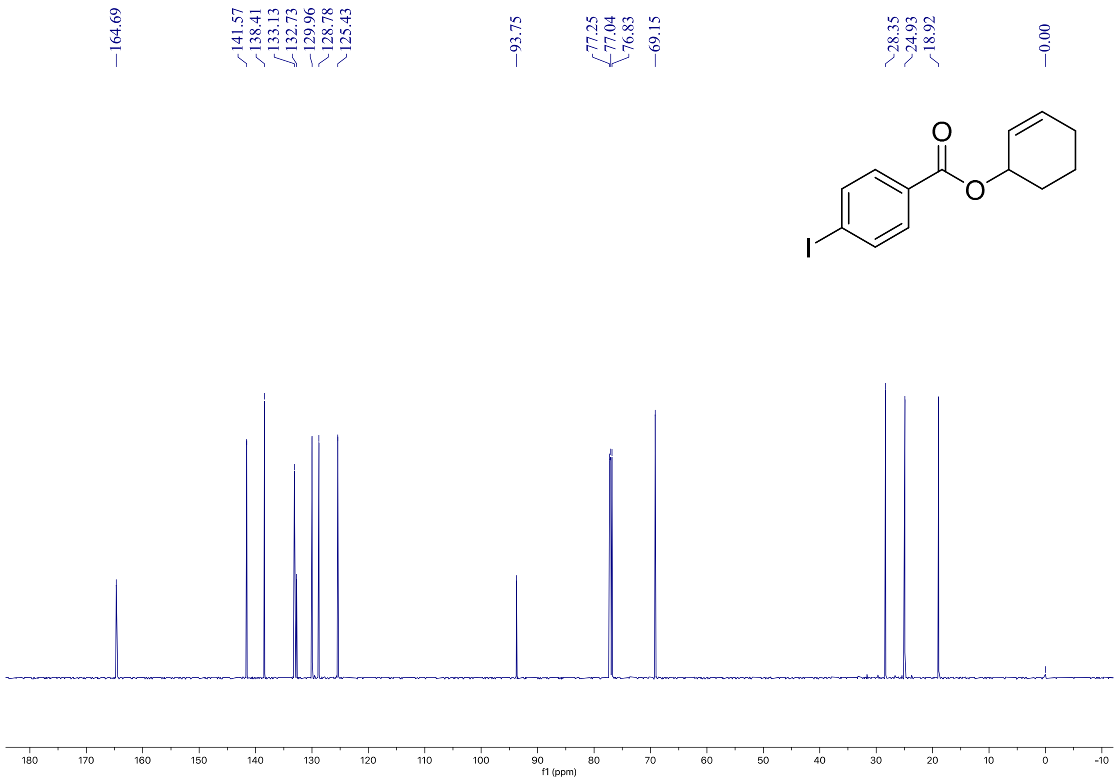

^{1}H NMR of cyclohex-2-en-1-yl 2,6-difluorobenzoate (**23a**)

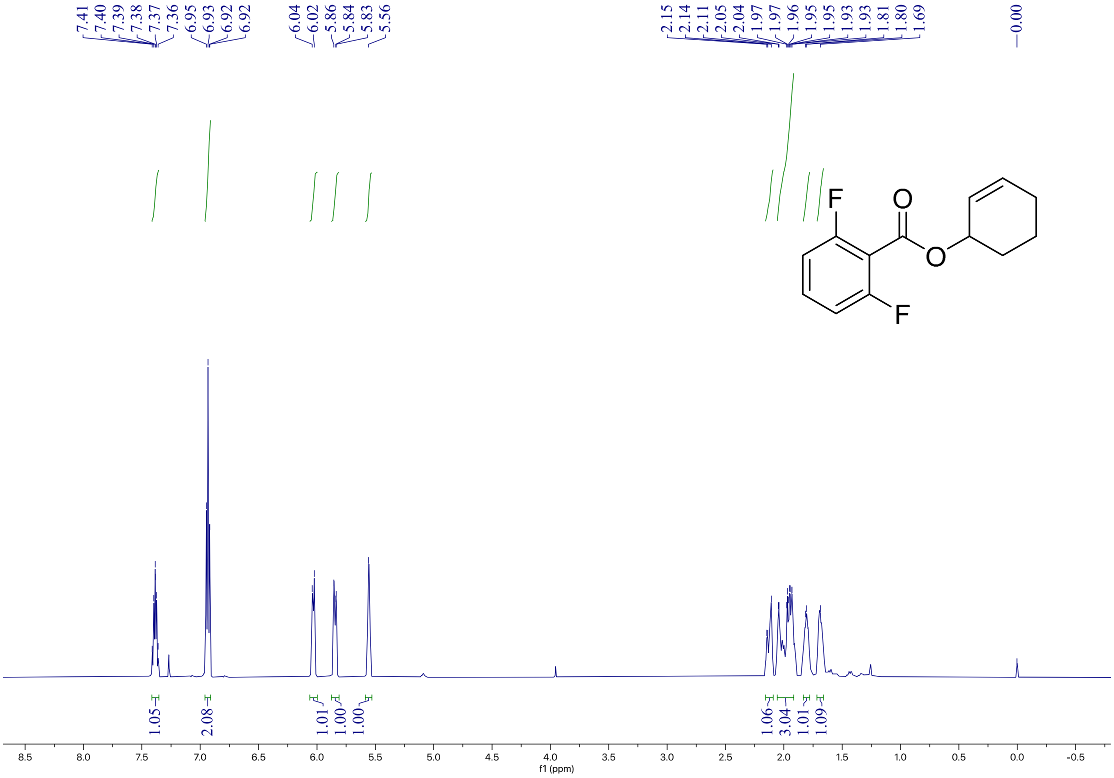

^{13}C NMR of cyclohex-2-en-1-yl 2,6-difluorobenzoate (**23a**)

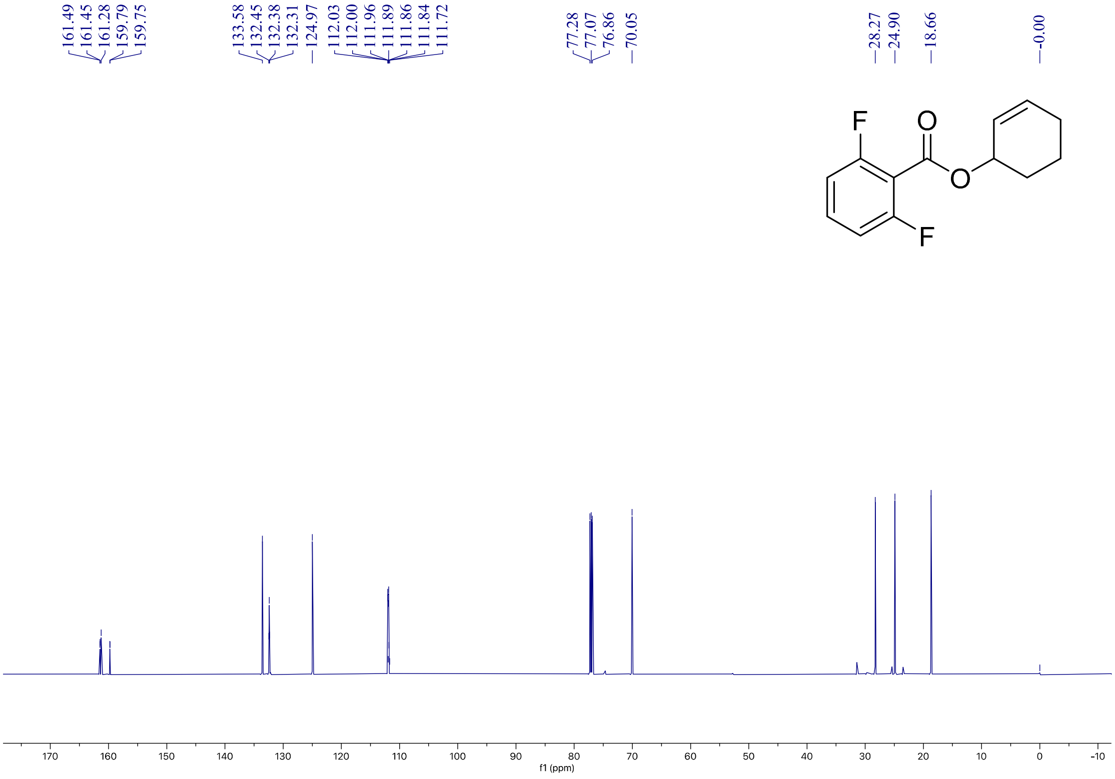

^{1}H NMR of cyclohex-2-en-1-yl 2,3,4,5,6-pentafluorobenzoate (**24a**)

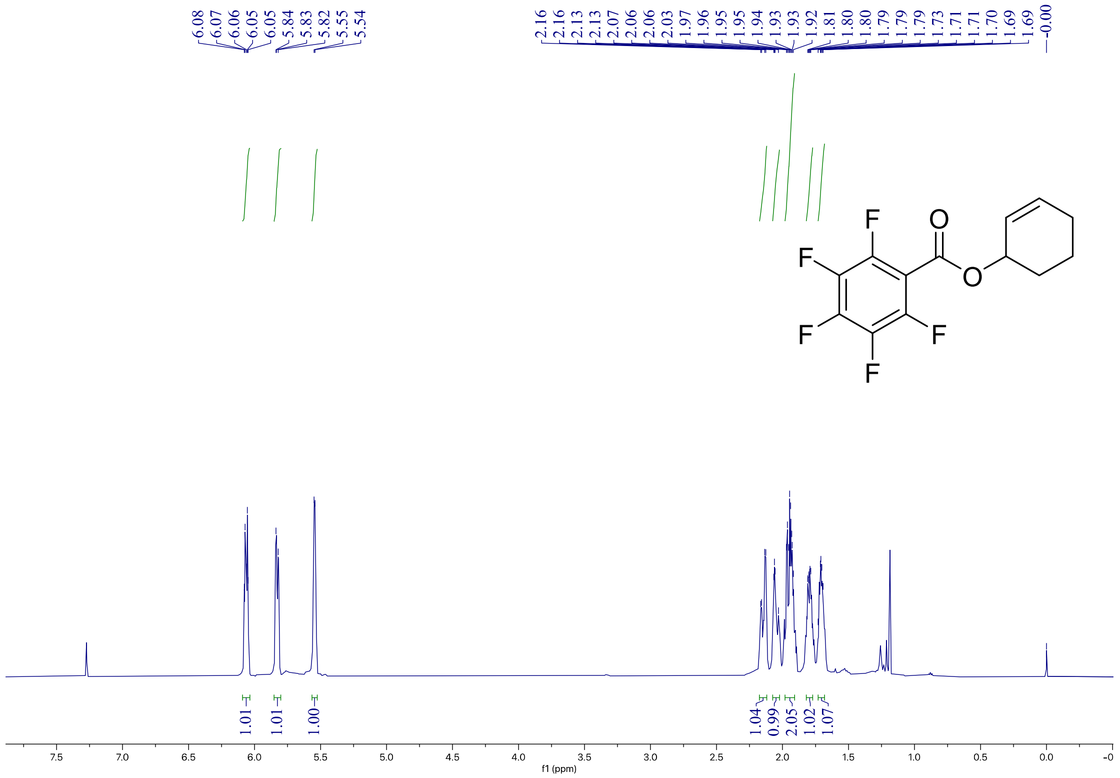

^{13}C NMR of cyclohex-2-en-1-yl 2,3,4,5,6-pentafluorobenzoate (**24a**)

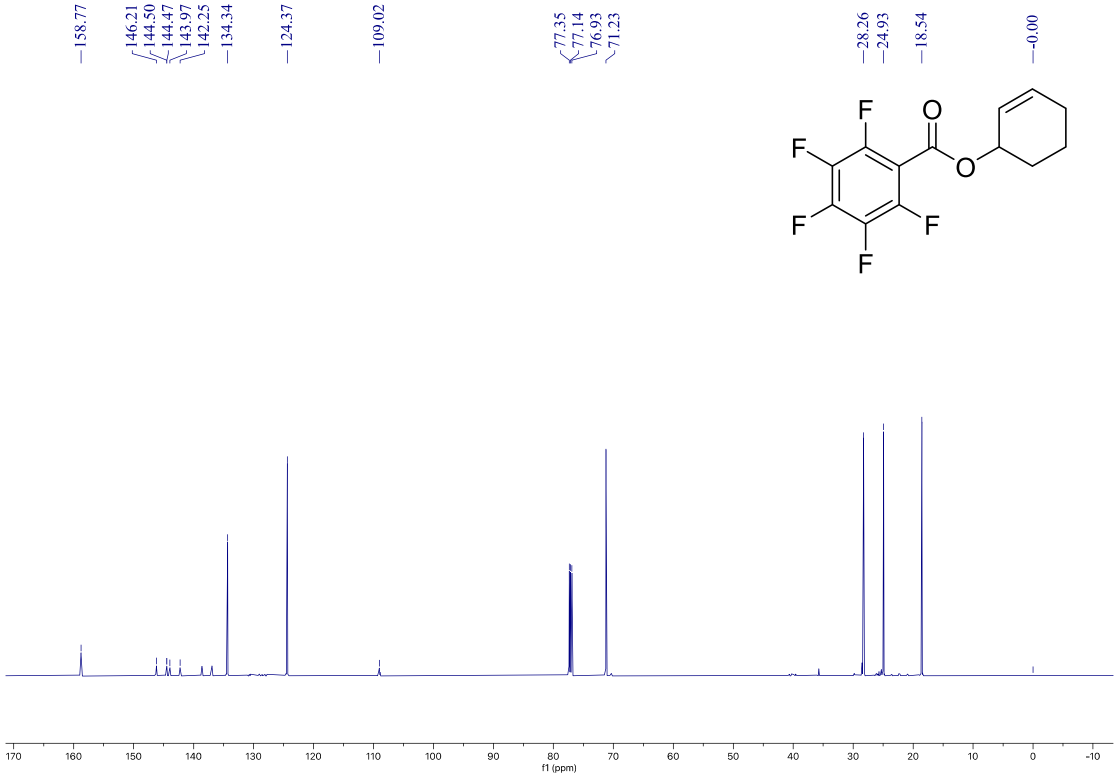

^{1}H NMR of cyclohex-2-en-1-yl 2-naphthoate (**25a**)

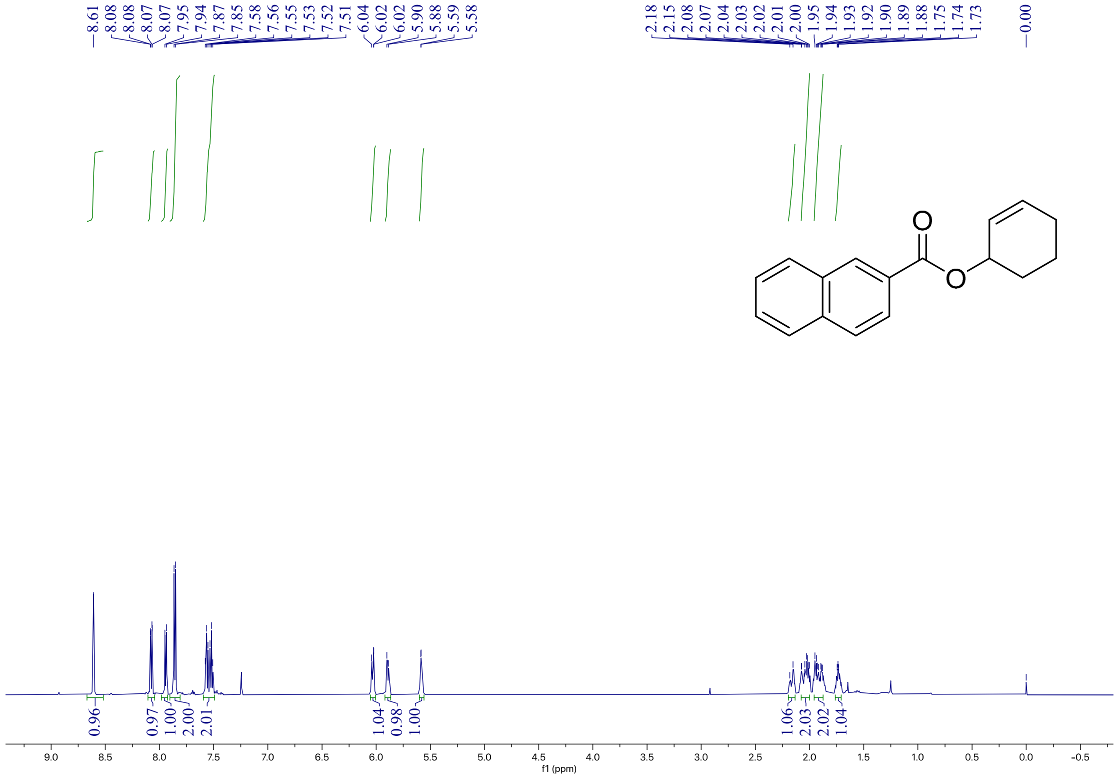

^{13}C NMR of cyclohex-2-en-1-yl 2-naphthoate (**25a**)

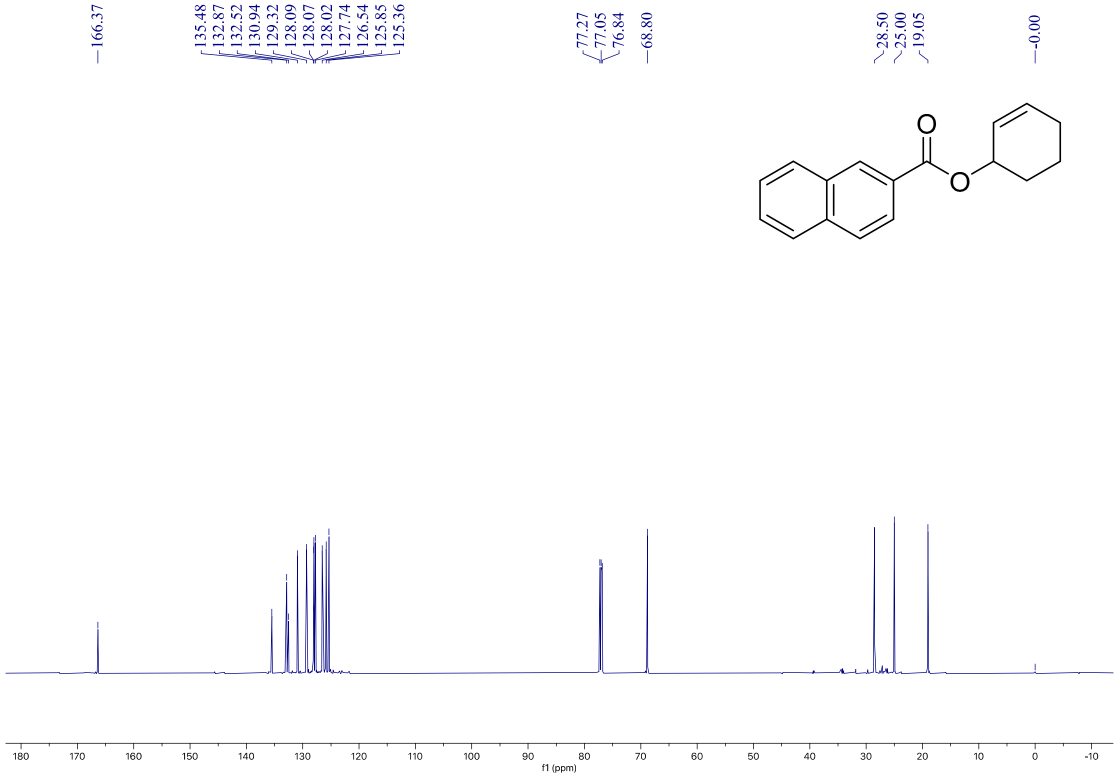

^{1}H NMR of cyclohex-2-en-1-yl ethyl 4-cyanobenzoate (**26a**)

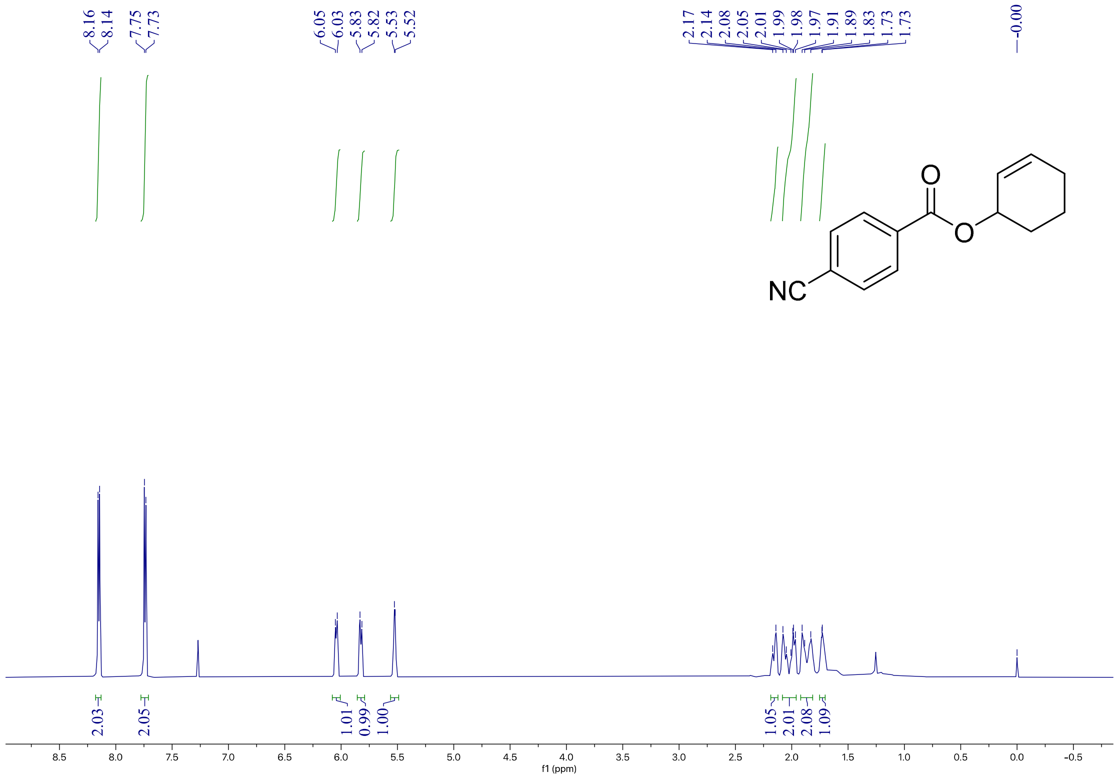

^{13}C NMR of cyclohex-2-en-1-yl ethyl 4-cyanobenzoate (**26a**)

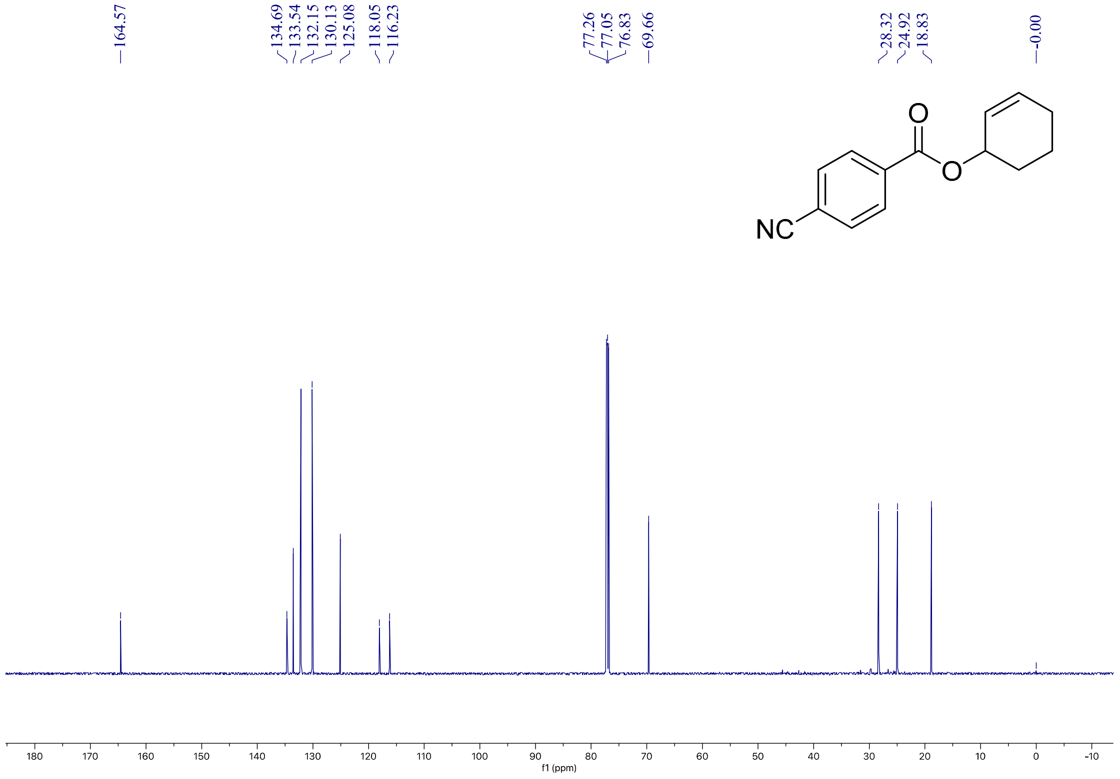

^{1}H NMR of cyclohex-2-en-1-yl furan-2-carboxylate (**29a**)

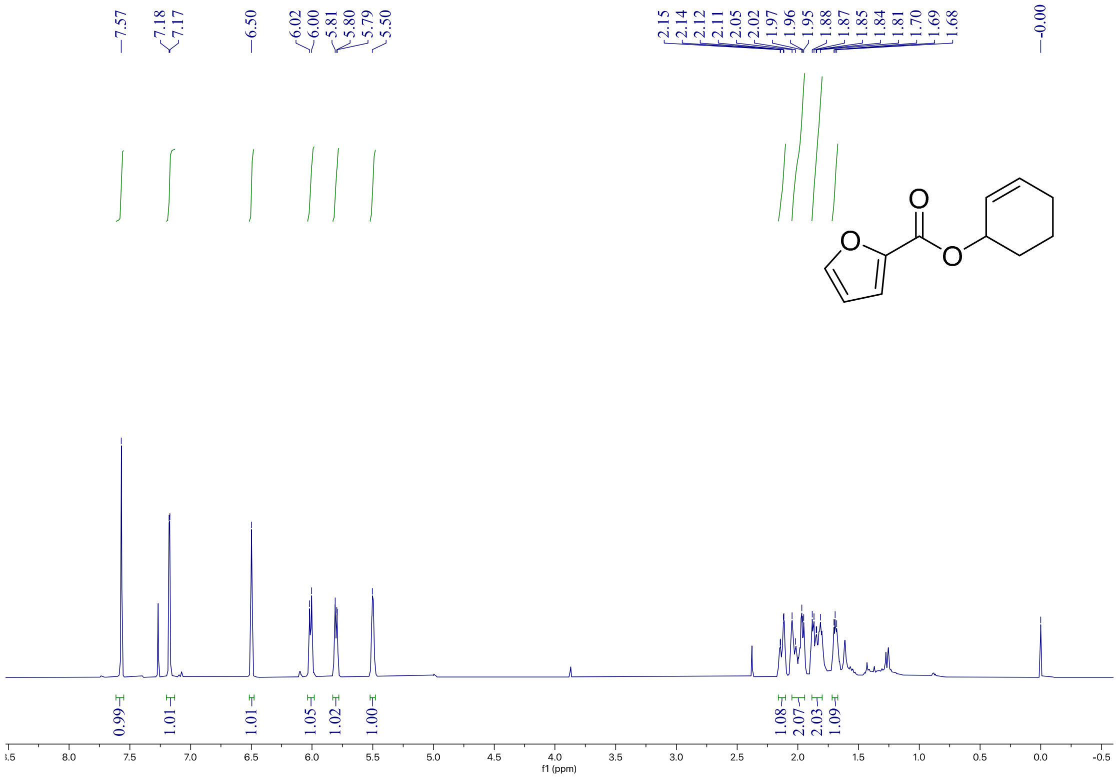

^{13}C NMR of cyclohex-2-en-1-yl furan-2-carboxylate (**29a**)

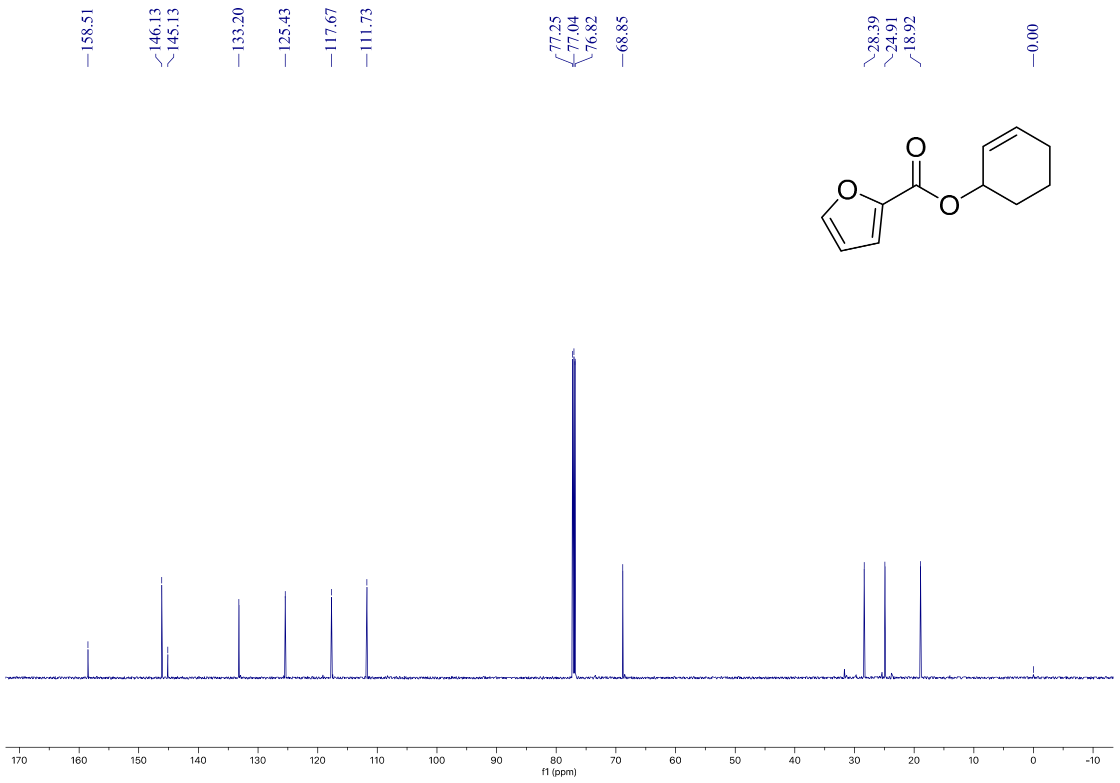

^{1}H NMR of cyclohex-2-en-1-yl thiophene-2-carboxylate (**30a**)

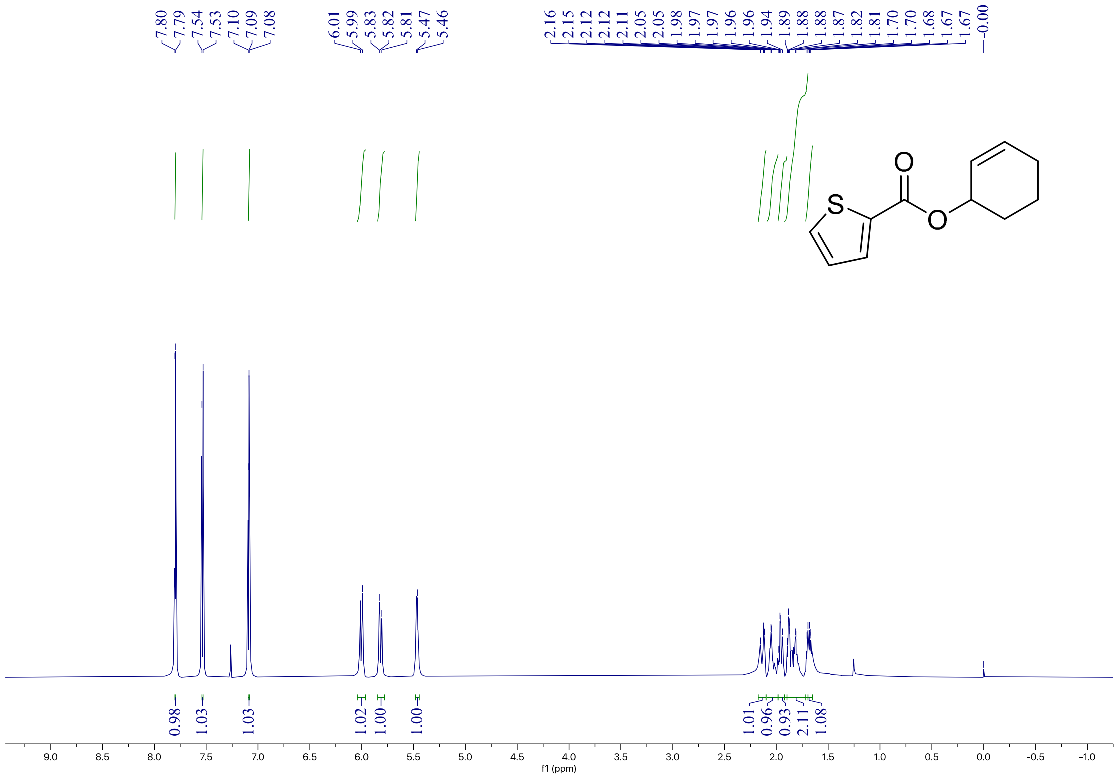

^{13}C NMR of cyclohex-2-en-1-yl thiophene-2-carboxylate (**30a**)

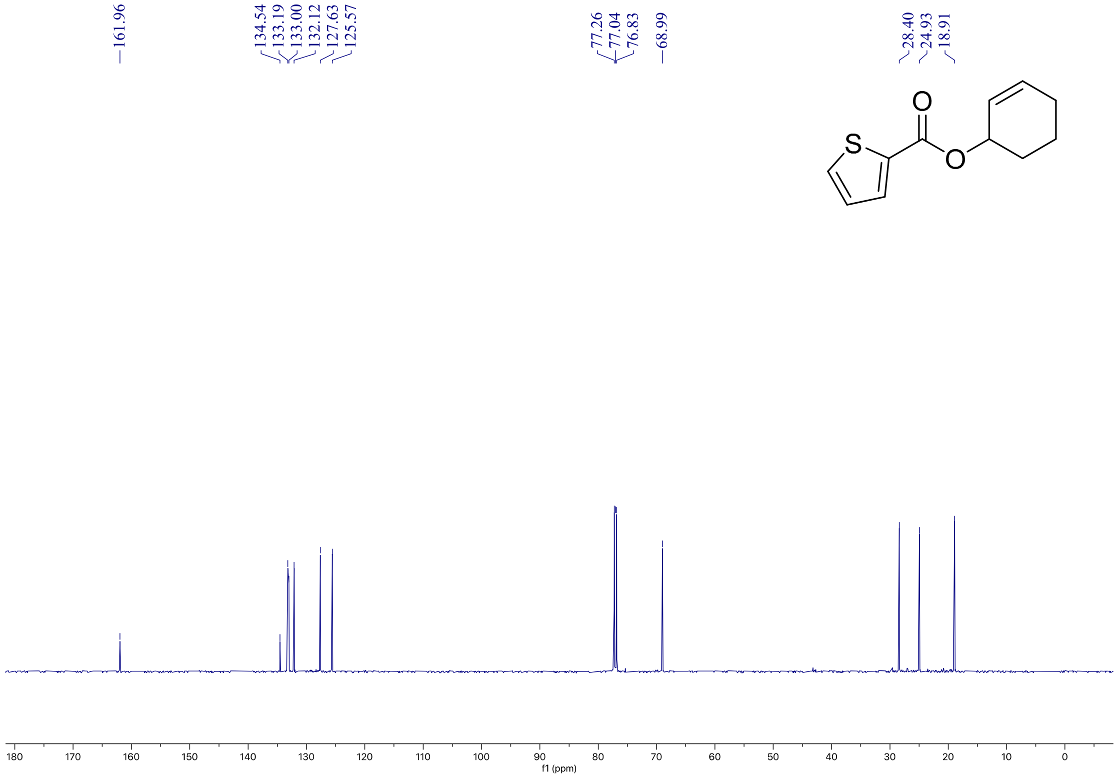

^{1}H NMR of cyclopent-2-en-1-yl benzoate (**1b**)

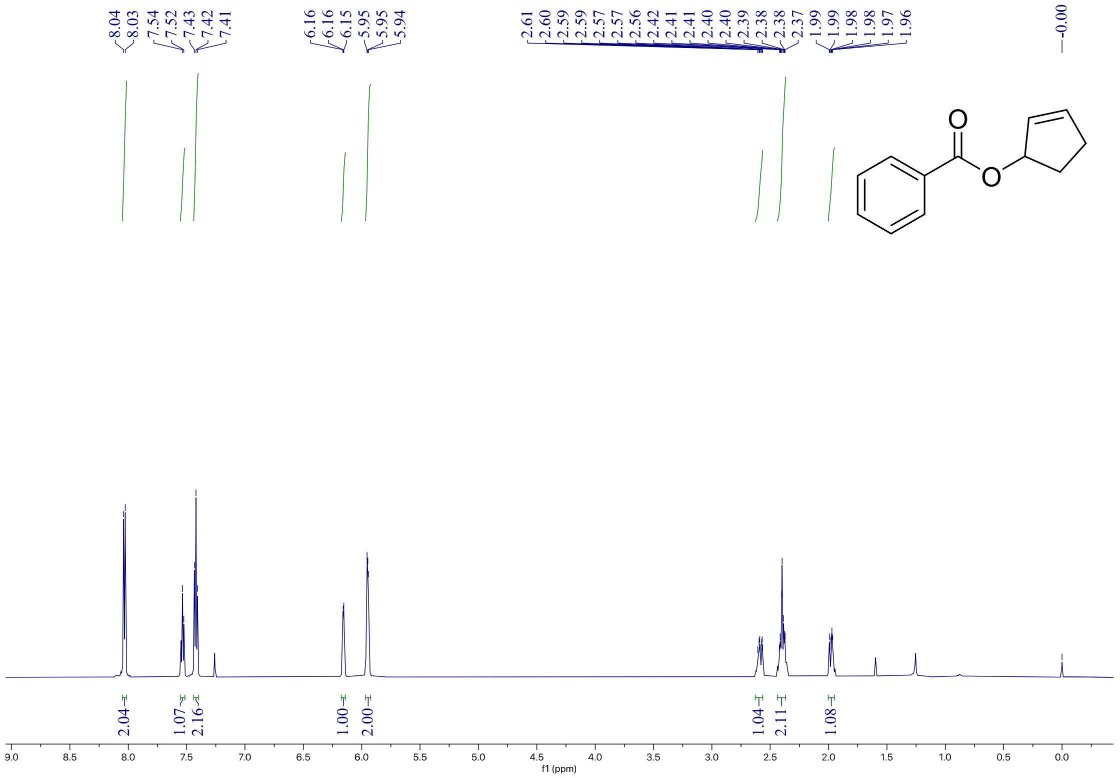

^{13}C NMR of cyclopent-2-en-1-yl benzoate (**1b**)

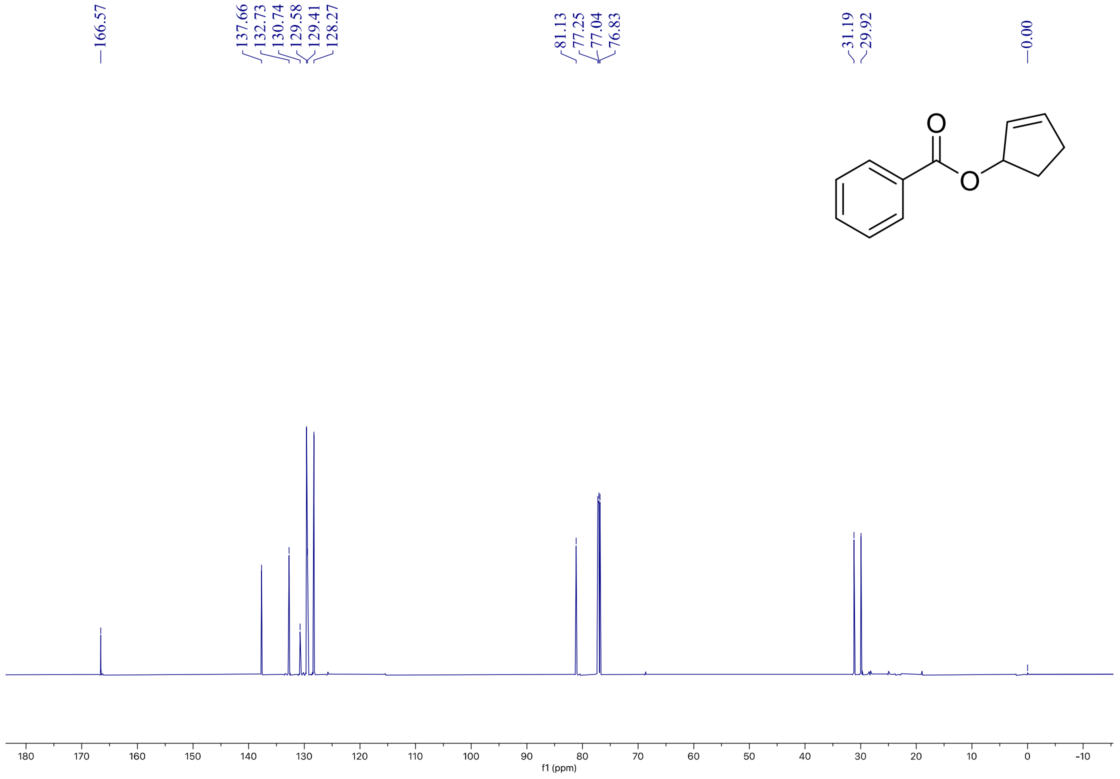

^{1}H NMR of cyclooct-2-en-1-yl benzoate (**1c**)

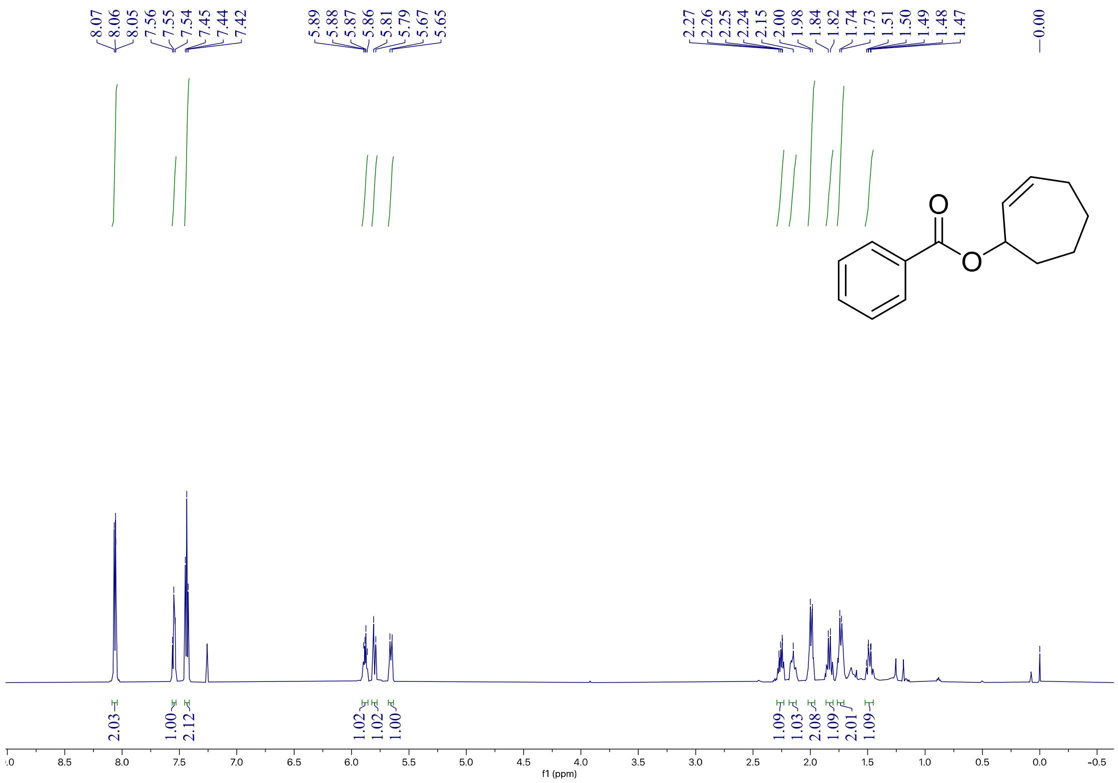

^{13}C NMR of cyclooct-2-en-1-yl benzoate (**1c**)

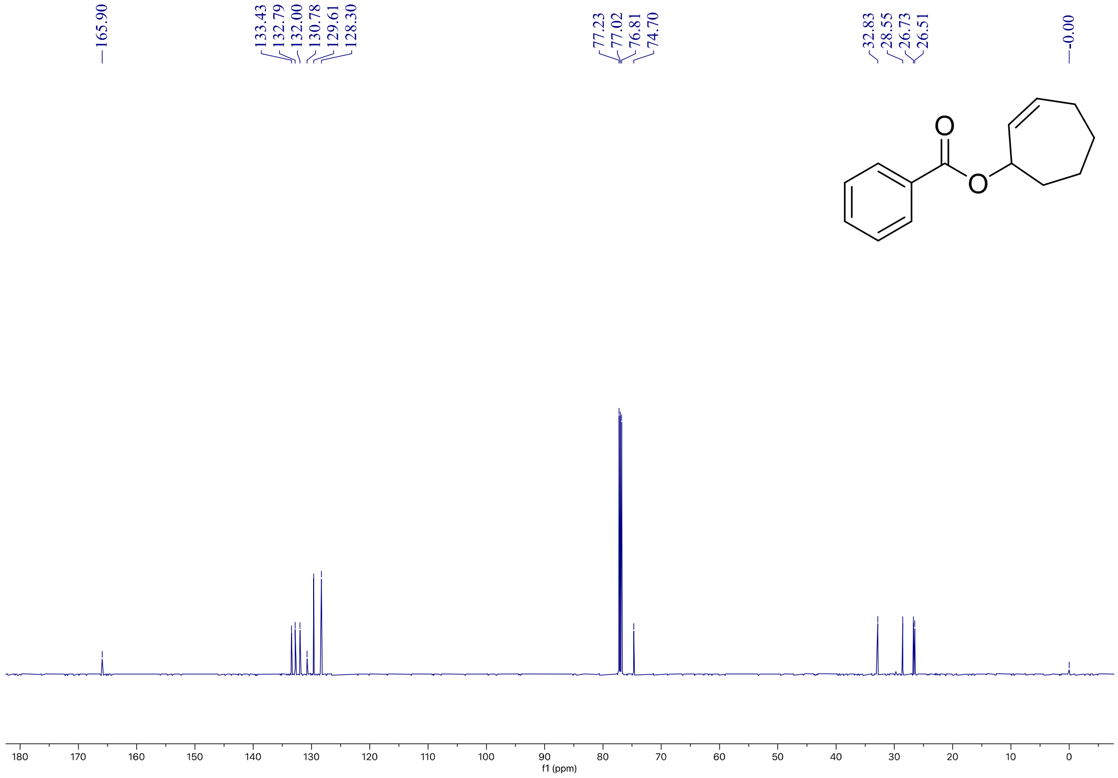

Supplement: Supplementary file 1 [file molecules-30-02232-s001.zip › molecules-3623551-supplementary.pdf]
